# Peripheral and central employment of acid-sensing ion channels during early bilaterian evolution

**DOI:** 10.7554/eLife.81613

**Published:** 2023-02-23

**Authors:** Josep Martí-Solans, Aina Børve, Paul Bump, Andreas Hejnol, Timothy Lynagh

**Affiliations:** 1 Michael Sars Centre, University of Bergen Bergen Norway; 2 Department of Biological Sciences, University of Bergen Bergen Norway; 3 Hopkins Marine Station, Department of Biology, Stanford University Pacific Grove United States; University of Vienna Austria; The University of Texas at Austin United States

**Keywords:** ion channels, invertebrates, spiralian, xenacoelomorph, hemichordate, Other

## Abstract

Nervous systems are endowed with rapid chemosensation and intercellular signaling by ligand-gated ion channels (LGICs). While a complex, bilaterally symmetrical nervous system is a major innovation of bilaterian animals, the employment of specific LGICs during early bilaterian evolution is poorly understood. We therefore questioned bilaterian animals’ employment of acid-sensing ion channels (ASICs), LGICs that mediate fast excitatory responses to decreases in extracellular pH in vertebrate neurons. Our phylogenetic analysis identified an earlier emergence of ASICs from the overarching DEG/ENaC (degenerin/epithelial sodium channel) superfamily than previously thought and suggests that ASICs were a bilaterian innovation. Our broad examination of ASIC gene expression and biophysical function in each major bilaterian lineage of Xenacoelomorpha, Protostomia, and Deuterostomia suggests that the earliest bilaterian ASICs were probably expressed in the periphery, before being incorporated into the brain as it emerged independently in certain deuterostomes and xenacoelomorphs. The loss of certain peripheral cells from Ecdysozoa after they separated from other protostomes likely explains their loss of ASICs, and thus the absence of ASICs from model organisms *Drosophila* and *Caenorhabditis elegans*. Thus, our use of diverse bilaterians in the investigation of LGIC expression and function offers a unique hypothesis on the employment of LGICs in early bilaterian evolution.

## eLife digest

Most animals on Earth, from worms to chimpanzees, belong to a group known as the bilaterians. Despite their rich variety of shapes and lifestyles, all these creatures share similarities – in particular, a complex nervous system where neurons can quickly relay electric signals. This is made possible by a class of proteins, known as ligand-gated ion channels, which are studded through the membrane of cells. There, they help neurons efficiently communicate with each other by converting external chemical information into internal electrical signals. Yet despite their importance, how and when these proteins have evolved remains poorly understood.

Marti-Solans et al. decided to explore this question by focusing on acid-sensing ion channels, a family which often forms the linchpin of bilaterian neural networks. They examined when these proteins first evolved (that is, in which putative ancestral animals) and where in the body. To do so, they combed through genetic data from all major bilaterian lineages as well as from non-biletarian groups; this included previously unexplored datasets that give insight into the type of cells in which a particular gene is active.

The analyses revealed that the channels are specific to bilaterians, but that they appeared earlier than previously thought, being present in the very first members of this group. However, at this stage, the proteins were mainly located in cells at the periphery of the body rather than in those from emerging neural circuits. This suggests that the channels were co-opted by nerve cells later on, when the nervous systems became more complex. The proteins being initially located in cells at the outer edge of the body could also explain why they are absent in bilaterian creatures such as fruit flies and nematode worms; these animals all belong to a lineage where growth takes place by shedding their external layers.

Acid-sensing ion channels are an important group of potential drug targets, often being implicated in pain and diseases of the nervous system. The work of Marti-Solans et al. offers an insight into the diversity of roles these proteins can play in the body, demonstrating once again how evolution can repurpose the same biophysical functions to serve a range of needs inside an organism.

## Introduction

Morphological and behavioral complexity of animals is facilitated by nervous systems in which peripheral neurons sense and convey information from the environment, and more centralized neurons integrate and dispatch information to effectors (Arendt, 2021; Liebeskind et al., 2016). Such sensory (environment → cell) and synaptic (cell → cell) signals rely on ligand-gated ion channels (LGICs), membrane proteins that convert chemical messages into transmembrane ionic currents within milliseconds (Pattison et al., 2019; Smart and Paoletti, 2012). LGICs are found in all multicellular animals (Metazoa) and even in outgroup lineages such as bacteria and plants (Chen et al., 1999; Chiu et al., 1999; Tasneem et al., 2005). But compared to animals without nervous systems (Porifera and Placozoa), animals with nervous systems (Ctenophora, Cnidaria, and Bilateria) present a larger and more diverse LGIC gene content, that is, more Cys-loop receptors, ionotropic glutamate receptors, and degenerin/epithelial sodium channel (DEG/ENaC) genes (Moroz et al., 2014). Although the evolution of the original nervous system(s) did not necessarily involve a rapid expansion of LGICs, the elaboration and refinement of nervous systems within particular lineages did, and this likely endowed bilaterian neurons with a sophisticated chemo-electric toolbox in the ancestors of today’s complex bilaterian animals (Arendt, 2021; Liebeskind et al., 2015; Moroz et al., 2014). Unfortunately, studies addressing the functional contribution of LGICs to early bilaterians are lacking (Heger et al., 2020), and we therefore lack crucial insight into evolution of the nervous system.

**Figure 1.**
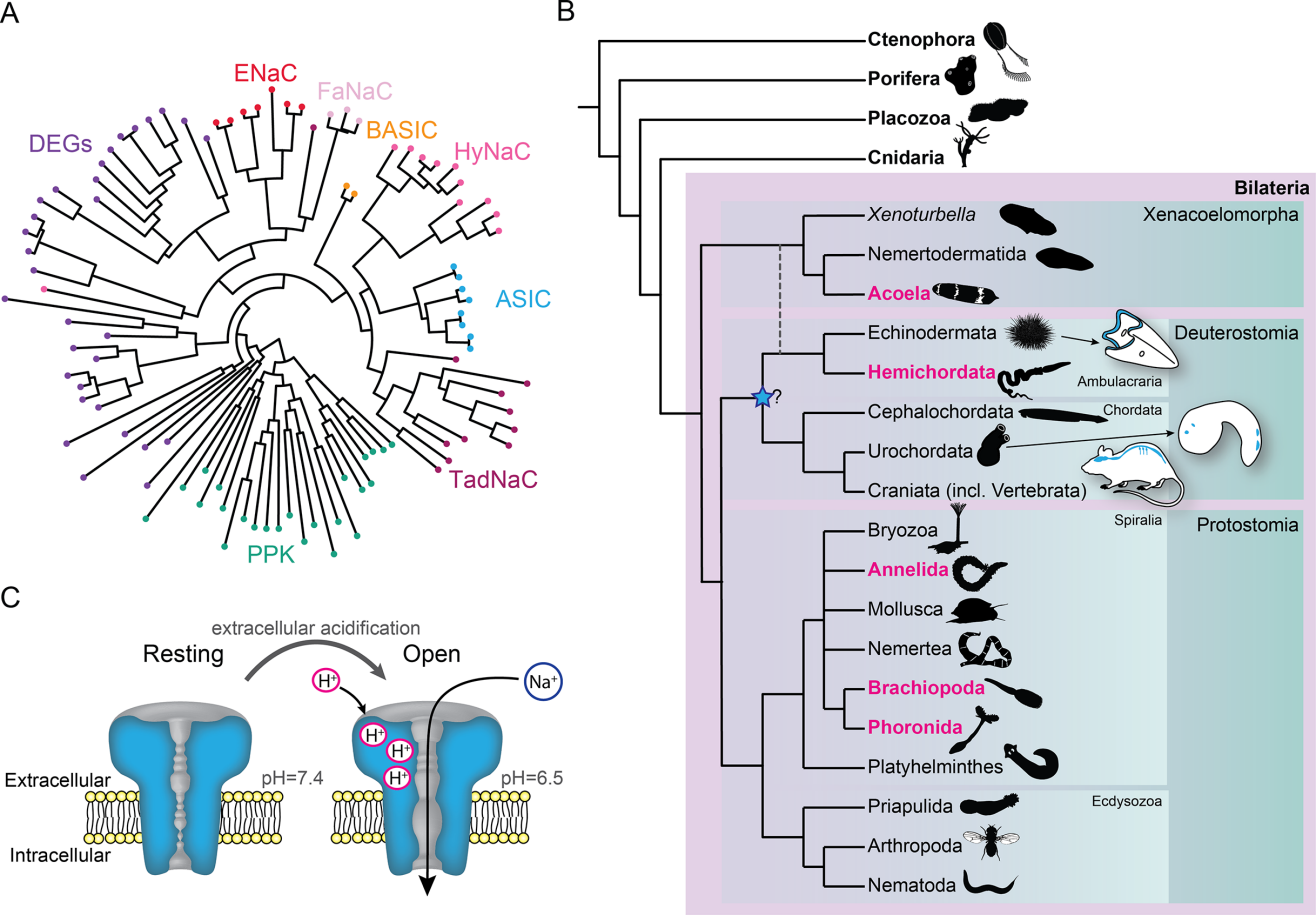
Overview of acid-sensing ion channels (ASICs) in Metazoa. (**A**) Abridged phylogenetic tree of the degenerin/epithelial sodium channel (DEG/ENaC) family showing major expansions of previously studied members (after Assmann et al., 2014; Elkhatib et al., 2019). (**B**) Phylogenetic tree of main animal groups highlighting groups studied in this work (magenta), known ASIC expression (blue), and previously suggested ASIC emergence (blue star). Dotted lines show alternative positions for Xenacoelomorpha. Cartoons show previously described and well-established ASIC protein and/or RNA expression in Chordata (rodent brain, spinal cord, and sensory ganglia, Foster et al., 2021; urochordate larva sensory vesicle and bipolar tail neurons, Coric et al., 2008) and Ambulacraria (echinoderm larva ciliary band, Slota et al., 2020). (**C**) ASIC function. At rest, the channel is closed and impermeable. Upon extracellular acidification, certain amino acid side chains in the ASIC extracellular domain are protonated, causing conformational changes that open the channel, allowing sodium ions to flow down their chemo-electric gradient across the membrane.

Diversity within the DEG/ENaC superfamily of channels exemplifies the novel tools that LGICs can offer an evolving nervous system. Several independent expansions of DEG/ENaC subfamilies have occurred, including degenerin channels (DEGs) in nematodes, pickpocket channels (PPKs) in arthropods, peptide-gated channels (*Hydra vulgaris* Na^+^ channels [HyNaCs]) in cnidarians, and acid-sensing ion channels (ASICs) in vertebrates (Assmann et al., 2014; Lynagh et al., 2018; Matthews et al., 2019; Ng et al., 2019; Tavernarakis et al., 1997; Figure 1A). ASICs are excitatory sodium channels gated by increased proton concentrations (drops in pH) and are widely expressed in the nervous system of rodents, with scattered expression in other cells, such as epithelia (Deval and Lingueglia, 2015; Figure 1B and C). ASICs in sensory neurons of skin, joints, and the gastrointestinal tract of rodents and humans contribute to pain, hyperalgesia, and touch (Holzer, 2015; Ikeuchi et al., 2008; Jones et al., 2004; Price et al., 2001), typifying a chemosensory role, whereas ASICs expressed postsynaptically in central neurons of rodents mediate depolarization in response to brief drops in synaptic pH during neurotransmission (Du et al., 2014; González-Inchauspe et al., 2017), indicating an additional, central and synaptic role. ASICs occur in all deuterostomes (Lynagh et al., 2018), one of the three major lineages within Bilateria (Figure 1B). But given the central or peripheral expression of ASICs in different deuterostomes (Coric et al., 2008; Lynagh et al., 2018; Slota et al., 2020) and uncertainty over the lineage in which ASICs emerged (Figure 1B), we lack an accurate assessment of how ASICs were deployed by diversifying bilaterians and thus the chance of better understanding how complex bilaterian nervous systems evolved. This would require a broad and comprehensive assessment of ASICs throughout the major bilaterian lineages.

We therefore performed a thorough phylogenetic investigation of metazoan DEG/ENaC genes, with a focus on ASICs, using unexplored transcriptomes and genomes, revealing ASICs throughout the Bilateria. Moreover, we analyzed gene expression using in situ hybridization and determined the electrophysiological properties of diverse ASICs from each major bilaterian lineage. Results from these experiments enable a new hypothesis on the evolution and function of ASICs during bilaterian evolution.

## Results

### Broader phylogenetic study identifies ASICs in Protostomia and Xenacoelomorpha

**Figure 2.**
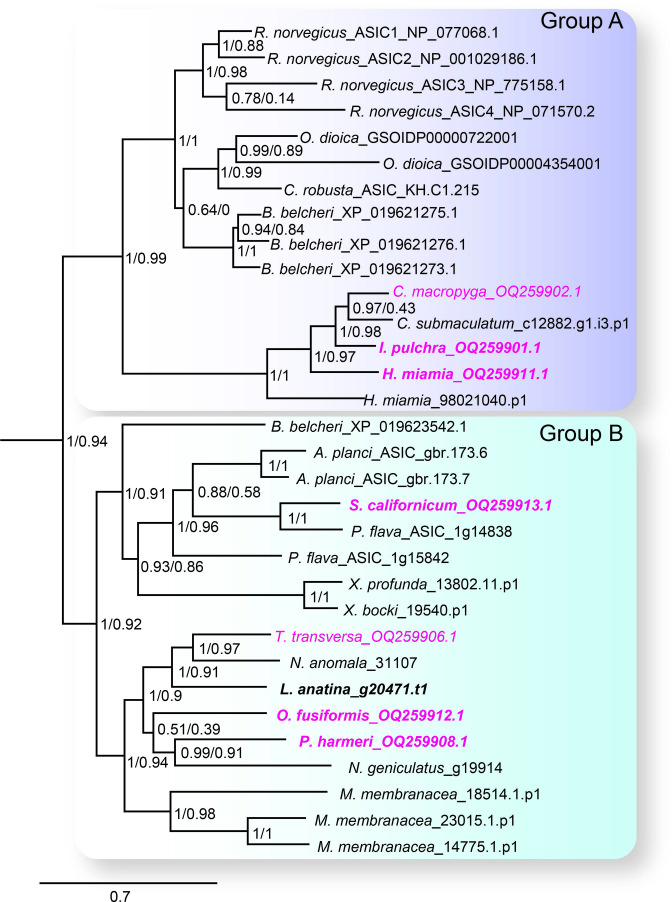
The acid-sensing ion channel (ASIC) branch of the degenerin/epithelial sodium channel (DEG/ENaC) family includes only bilaterian genes. ASIC branch from phylogenetic tree of DEG/ENaC family including 700 amino acid sequences from 47 metazoans (Figure 2—figure supplement 1). Genes analyzed experimentally in this study are in magenta (gene expression) and/or bold (electrophysiology). Scale bar: amino acid substitutions per site. aBayes (left) and aLRT SH-like (right) likelihood-based support values indicated.

We first sought a definitive picture of how broadly ASIC genes are conserved throughout the five metazoan lineages of Bilateria, Cnidaria, Porifera, Placozoa, and Ctenophora (see Figure 1B for phylogenetic relationship of these lineages). To this end we utilized previously unexplored transcriptomes combined with canonical resources to search for DEG/ENaC genes from all lineages. The phylogenetic analysis of these 700 sequences from 47 species shows a well-supported clade of ASICs (Figure 2 and Figure 2—figure supplement 1). The ASIC branch consists of two sub-clades ‘A’ and ‘B’, both of which include bona fide ASICs from deuterostome bilaterians (Lynagh et al., 2018). No ASICs were identified in Cnidaria, Porifera, Placozoa, and Ctenophora.

**Figure 2—figure supplement 1.**
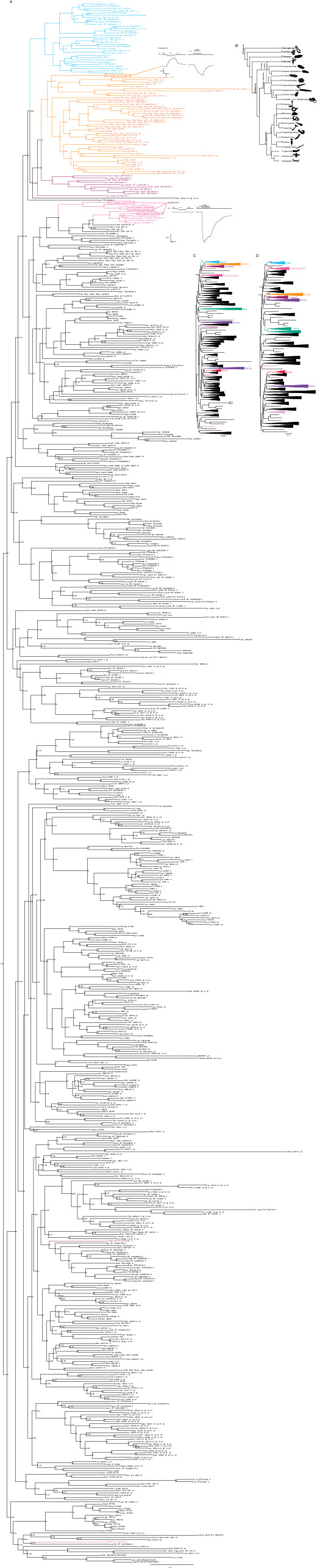
Degenerin/epithelial sodium channel (DEG/ENaC) gene tree. Maximum likelihood trees of 700 DEG/ENaC amino acid sequences from 47 animals (using LG + G substitution model; see Materials and methods, Survey and phylogenetic analysis). Branch support inferred by fast likelihood-based methods aBayes (**A and C**) and aLRT SH-like (**D**). Aaur, *Aurelia aurita* (Cnidaria); Agra, *Acanthopleura granulata* (Mollusca); Apla, *Acanthaster planci* (Echinodermata); Aque, *Amphimedon queenslandica* (Porifera); Bbel, *Branchiostoma belcheri* (Chordata); Bpli, *Brachionus plicatilis* (Rotifera); Cele, *Caenorhabditis elegans* (Nematodea); Cgig, *Crassostrea gigas* (Mollusca); Cmac, *Convolutriloba macropyga* (Xenacoelomorpha); Crob, *Ciona robusta* (Chordata); Cscu, *Centruroides sculpturatus* (Arthropoda); Csub, *Childia submaculatum* (Xenacoelomorpha); Dgig, *Dendronephthya gigantea* (Cnidaria); Dgyr, *Dinophilus gyrociliatus* (Annelida); Dmel, *Drosophila melanogaster* (Arthropoda); Dpul, *Daphnia pulex* (Arthropoda); Esen, *Epiphanes senta* (Rotifera); Hmia, *Hofstenia miamia* (Xenacoelomorpha); Hspi, *Halicryptus spinulosus* (Priapulida); Hvul, *Hydra vulgaris* (Cnidaria); Ipul, *Isodiametra pulchra* (Xenacoelomorpha); Lana, *Lingula anatina* (Brachiopoda); Llon, *Lineus longissimus* (Nemertea); Lsqu, *Lepidodermella squamata* (Gastrotricha); Lvir, *Lineus viridis* (Nemertea); Mlei, *Mnemiopsis leidyi* (Ctenophora); Mmem, *Membranipora membranacea* (Bryozoa); Msti, *Meara stichopi* (Xenacoelomorpha); Nano, *Novocrania anomala* (Brachiopoda); Ngen, *Notospermus geniculatus* (Nemertea); Nvec, *Nematostella vectensis* (Cnidaria); Nwes, *Nemertoderma westbladi* (Xenacoelomorpha); Odio, *Oikopleura dioica* (Chordata); Ofus, *Owenia fusiformis* (Annelida); Pbac, *Pleurobranchia bachei* (Ctenophora); Pcau, *Priapulus caudatus* (Priapulida); Pfla, *Ptychodera flava* (Hemichordata); Phar, *Phoronopsis harmeri* (Phoronida); Pvit, *Prostheceraeus vittatus* (Platyhelminthes); Pvul, *Pontonema vulgare* (Nematoda); Rnor, *Rattus norvegicus* (Chordata); Scal, *Schizocardium californicum* (Hemichordata); Smed, *Schmidtea mediterranea* (Platyhelminthes); Spad, *Spadella sp* (Chaetognatha); Tadh, *Trichoplax adhaerens* (Placozoan); Ttra, *Terebratalia transversa* (Brachiopoda); Xboc, *Xenoturbella bocki* (Xenacoelomorpha); Xpro, *Xenoturbella profunda* (Xenacoelomorpha). Reponses to increased proton concentrations at oocytes expressing channels closely related to acid-sensing ion channels (ASICs) are shown: heterotrimeric cnidarian *Hydra vulgaris* Na^+^ channel (HyNaC) (pink); rat bile acid-sensitive ion channel (BASIC) (orange). Other ligands are the peptide Hydra-RFamide-I ((pE)WLGGRFamide; pE; pyroglutamate) and the bile acid sodium ursodeoxycholic acid (NaUDCA). Schematic phylogenetic tree of main animal groups (**B**).

**Figure 2—figure supplement 2.**
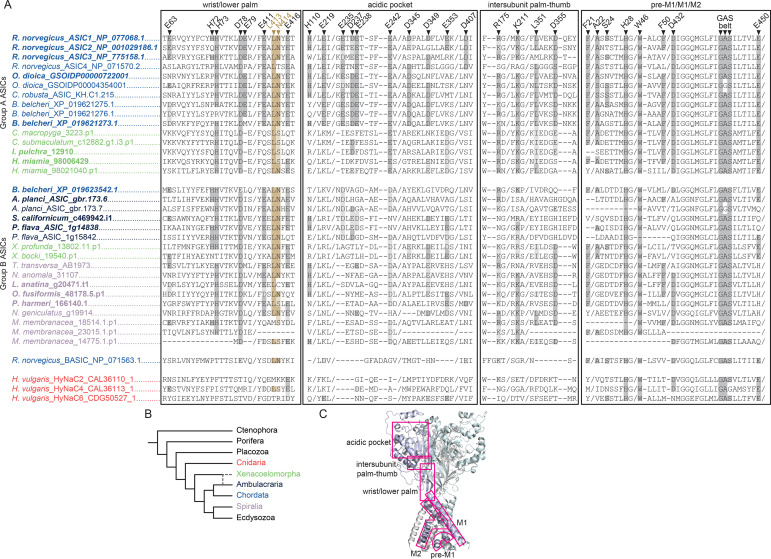
Sequence alignment of relevant domains in acid-sensing ion channels (ASICs). (**A**) Protein sequence alignment of the wrist/lower palm, the acidic pocket, intersubunit palm-thumb, and pre-M1/M1/M2 of Group A and B ASICs and selected bile acid-sensitive ion channels (BASICs) and *Hydra vulgaris* Na^+^ channels (HyNaCs). Selected rat ASIC1a residues implicated in proton sensitivity and channel function are numbered above (see references in the main text). Verified proton-activated channels in bold. Colors refer to B. (**B**) Schematic phylogenetic tree of main animal groups used in A. (**C**) Cartoon diagram of the chick ASIC1 homotrimeric channel crystal structure, PDB number 6VTL. Regions compared in A in pink boxes.

Bilateria is divided into three major groups, Deuterostomia (above), Protostomia (e.g. arthropods and molluscs), and Xenacoelomorpha (acoelomorph flatworms without nephridia). In contrast to previous studies, we detected ASICs in protostomes and xenacoelomorphs (Figure 2). These include putative ASICs from seven protostome species—the annelid *Owenia fusiformis*, the nemertean *Notospermus geniculatus*, the brachiopods *Terebratalia transversa*, *Novocrania anomala* and *Lingula anatina*, the phoronid *Phoronopsis harmeri*, the bryozoan *Membranipora membranacea*, and six xenacolomorph species, the acoels *Hofstenia miamia*, *Isodiametra pulchra*, *Convolutriloba macropyga*, and *Childia submaculatum*, and the xenoturbellans *Xenoturbella bocki* and *Xenoturbella profunda*. Our analysis also included the hemichordate *Schizocardium californicum*, whose ASIC groups with previously reported hemichordate and echinoderm ASICs (Lynagh et al., 2018), indicative of broad conservation of ASICs in Ambulacraria, the deuterostome group including hemichordates and echinoderms. This shows that ASIC genes are present in the three major bilaterian groups of deuterostomes, protostomes, and xenacoelomorphs and absent from all other lineages, suggesting that ASICs diverged from other DEG/ENaC genes after the Cnidaria/Bilateria split and before the Bilateria diversified.

Protostomes are divided into Spiralia (such as annelids, molluscs, brachiopods, and phoronids), and Ecdysozoa (such as the nematode *Caenorhabditis elegans* and arthropod *Drosophila melanogaster*) (Figure 1B). Notably, the ASIC clade includes no ecdysozoan genes, although our analysis included DEG/ENaC genes from the nematode *Pontonema vulgare*, pan-arthropods *Centruroides sculpturatus* and *Daphnia pulex*, and priapulids *Priapulus caudatus* and *Halicryptus spinulosus*. We also see no putative ASICs in certain spiralians, including the molluscs *Acanthopleura granulata* and *Crassostrea gigas*, the platyhelminths *Prostheceraeus vittatus* and *Schmidtea mediterranea* and in certain xenacoelomorphs, including the nemertodermatids *Meara stichopi* and *Nemertoderma westbladi*. This analysis suggests that ASICs, although found throughout Bilateria, were lost in the lineage to the Ecdysozoa shortly after the Ecdysozoa/Spiralia split and were also lost in certain other bilaterian lineages such as molluscs and nemertodermatids.

Amino acid sequence comparison suggests that diverse ASIC protomers share a conserved structure of a large extracellular domain of ~300 residues between two transmembrane helices, and 30–100 residue intracellular N and C terminal tails. Newly described ASICs are 24–35% identical to rat ASIC1-3, the latter of which share 48–51% identity with each other. The second transmembrane helix is the most conserved segment (38–62%), followed by the extracellular domain (31–39%), with the first transmembrane helix being the most diverse (14–44%). Two clusters of residues in the extracellular domain, one in the acidic pocket and one in the palm/wrist domain, contain protonatable residues that are required for channel activation (Jasti et al., 2007; Paukert et al., 2008; Rook et al., 2021; Vullo et al., 2017). Residues H73, D78, E411, and E416 (rat ASIC1a numbering) from the palm/wrist domain and K211 located in an intersubunit palm-thumb interaction region are fairly conserved throughout all ASICs, however conservation of the acidic pocket was only obvious in chordate Group A ASICs, suggesting that this became important for the gating of, for example, rat ASIC1a more recently (Figure 2—figure supplement 2).

### ASICs are expressed in two domains in Xenacoelomorpha

The results from the phylogenetic analyses suggest that ASICs emerged during early bilaterian evolution, and we next sought evidence for potential biological roles by investigating the expression of ASIC genes throughout the major bilaterian lineages. The precise relationships between these lineages are under debate, but Xenacoelomorpha forms a putative sister group to all remaining Bilateria (Cannon et al., 2016; Kapli et al., 2021; Laumer et al., 2019; Philippe et al., 2019). Therefore, we investigated ASIC expression in Xenacoelomorpha, utilizing the acoels *I. pulchra*, *H. miamia*, and *C. macropyga*. Xenacoelomorphs show a large variation in nervous system architecture, but all possess a basal epidermal nerve plexus and some species, more internally, an anterior condensation of neurons or ‘brain’ (Hejnol and Pang, 2016). *I. pulchra* and *C. macropyga* also possess longitudinal bundles of neurons (Achatz and Martinez, 2012; Martín-Durán et al., 2018; Sikes and Bely, 2008). External to this nervous system in acoels is generally a sheet of longitudinal and ring muscles and, more externally, ciliated epithelial cells mediating locomotion (Haszprunar, 2016; Martin, 1978; Raikova et al., 2016).

**Figure 3.**
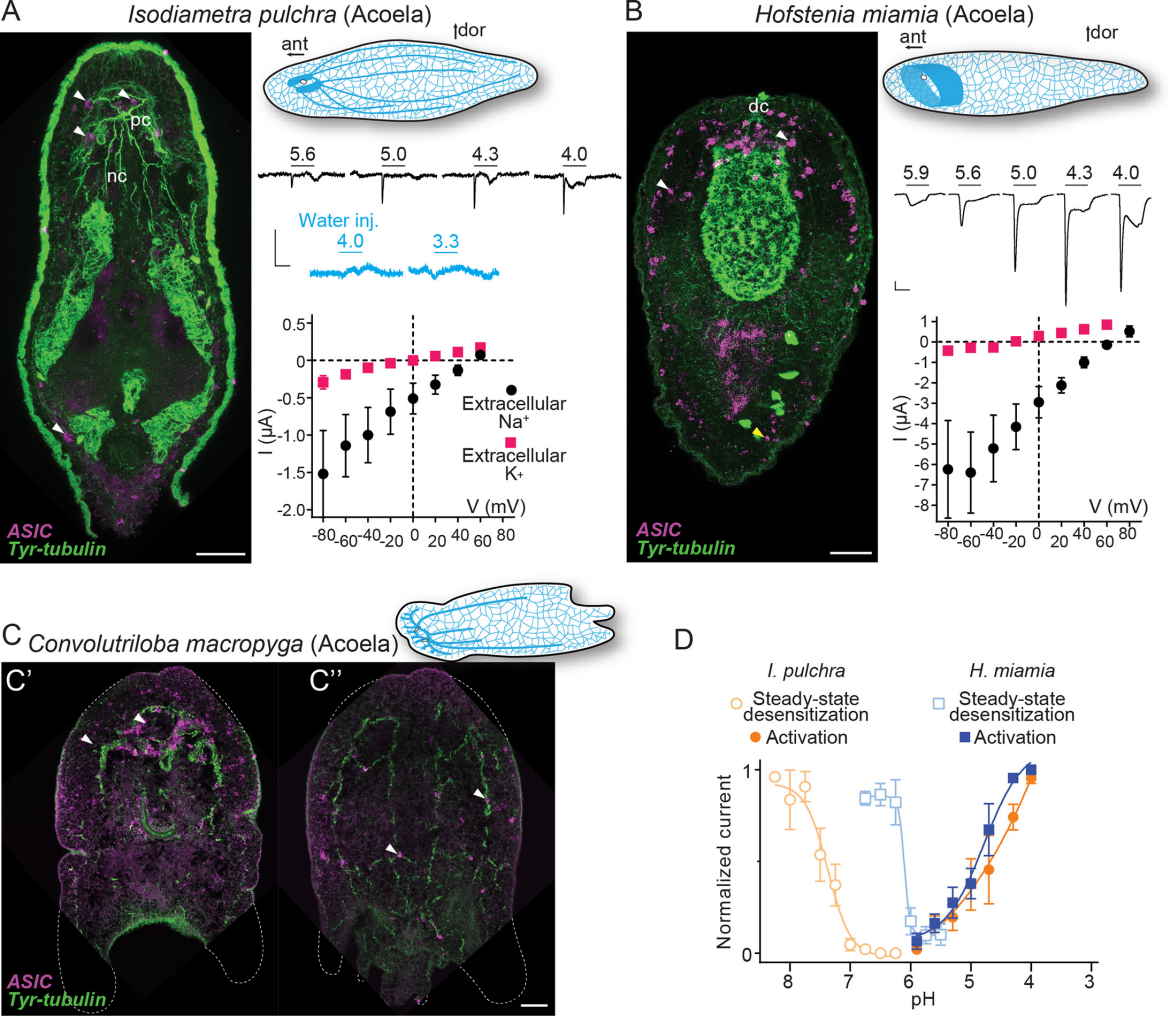
Expression and function of acid-sensing ion channels (ASICs) in Xenacoelomorpha. (**A,B**) Left: Fluorescent confocal micrograph showing ASIC mRNA expression (magenta) and tyrosinated tubulin immunoreactivity (green), scale bar: 40 µm. Upper right: Cartoons illustrating morphology and nervous system (blue) after Martín-Durán et al., 2018. ant, anterior; dor, dorsal; dc, dorsal commissure; nb, neurite bundles; pc, posterior commissure. Mid-right: Proton-gated currents in xenacoelomorph ASIC-expressing or water-injected *Xenopus laevis* oocytes (scale bars: x, 5 s; y, 0.5 μA). Lower right: Mean (± SEM) pH 4-gated current (I, μA) at different membrane potentials (V, mV) in the presence of 96 mM extracellular NaCl or KCl (n=5–7). Reversal potential (V_rev_) was read off these plots and the difference between V_rev,NaCl_ and V_rev,KCl_ was used to calculate relative ion permeability (P_Na+_/P_K+_, *Materials and methods*). (**C**) ASIC mRNA expression (magenta) and tyrosinated tubulin immunoreactivity (green) in *Convolutriloba macropyga*. As *C. macropyga* is larger, images of slightly ventral (**C'**) and dorsal (**C''**) planes are shown. (**D**) Filled symbols: Mean (± SEM) normalized current amplitude in response to increasing proton concentrations (activation, n=6–8). Open symbols: Mean (± SEM) normalized current amplitude in response to pH 4 following pre-incubation in decreasing pH (steady-state desensitization, n=5–6). Figure 3—source data 1.Numerical data contributing to Figure 3.

From a dorsal view, expression of *I. pulchra* ASIC mRNA can be detected in several cells across the central anterior of the adult animal (Figure 3A). This is the location of the *I. pulchra* brain, where, for example, serotonergic and peptidergic neurons form lateral lobes, connected by a frontal ring and a posterior commissure, and from which four pairs of nerve cords extend posteriorly (Figure 3A; Achatz and Martinez, 2012). Fluorescent confocal micrographs show that the ASIC-expressing cells are associated with the posterior commissure of the brain and the dorsal and lateral neurite bundles, including very posterior positions (Figure 3A, white arrowheads). Double fluorescent in situ hybridization of ASIC with *choline acetyltransferase* (*ChAT*) probes revealed that part of ASIC-expressing cells overlap with few cholinergic neurons of the brain (Figure 3—figure supplement 1A). In *H. miamia*, we observed high ASIC expression in cells throughout the anterior third of the animal (Figure 3B), correlating with the brain-like anterior condensation of neurons in this acoel (Hulett et al., 2020). Double fluorescent in situ hybridization showed ASIC localization in two anterior cell populations: GABAergic neurons expressing glutamate decarboxylase and directly adjacent cells (Figure 3—figure supplement 1B). This might reflect emerging single-cell RNA sequencing data on *H. miamia* identifying three distinct ASIC-expressing cell types: two muscle-like and one neuron-like (Hulett et al., 2022). Similar to *I. pulchra*, the ASIC signal is strongest near the dorsal commissure but, in contrast, also in scattered cells across the animal, including in the very periphery (Figure 3B, yellow arrowhead). *C. macropyga* ASIC expression was high and broad. In the anterior, ASIC-expressing cells are clearly associated with the brain (Figure 3C', white arrowheads) and potentially in the same cells expressing the neuronal marker *sine-oculis like* 3/6 (*Six3/6*) (Figure 3—figure supplement 1C). Throughout the animal, including the very periphery, there is medium to high ASIC expression, perhaps associated with locomotory ciliated cells covering the animal or the extensive nerve plexus (Figure 3C''). In summary, all Xenacoelomorpha species investigated here exhibit expression in two domains, in the brain, as well as dispersed, peripheral expression. Thus, ASICs may have had central, integrative, and peripheral sensory functions in early acoels.

**Figure 3—figure supplement 1.**
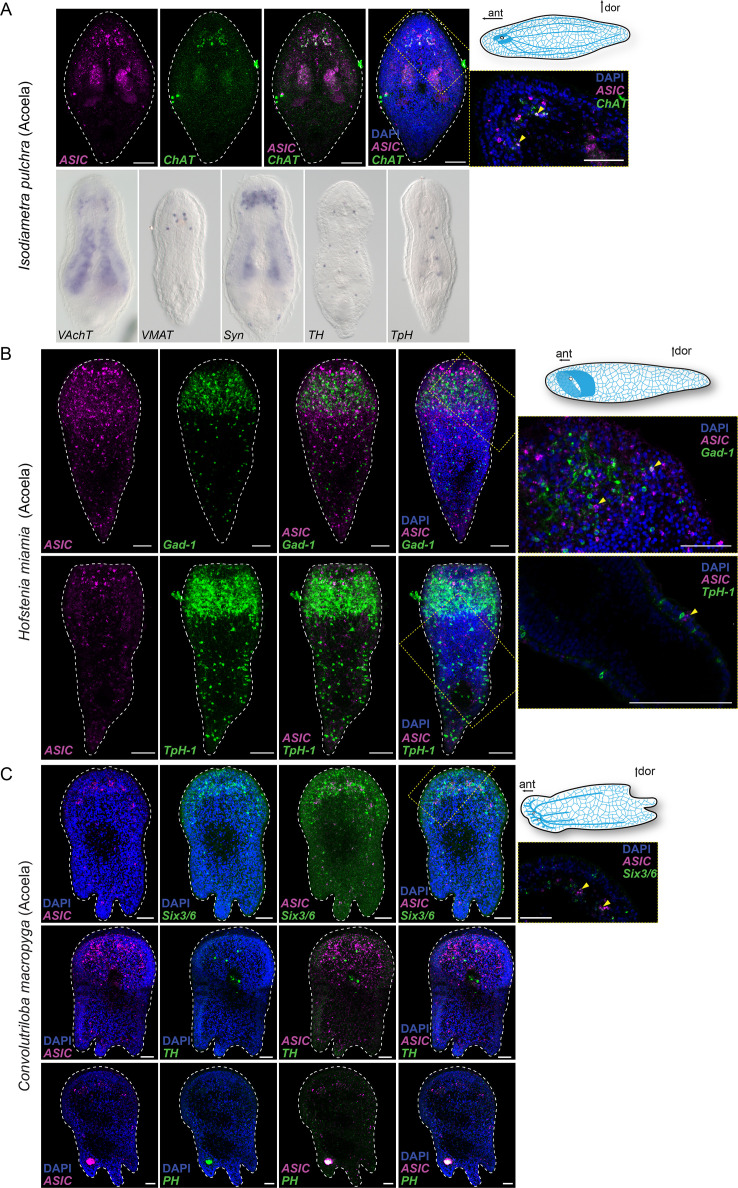
Expression of neuronal markers in Xenacoelomorpha: *Isodiametra pulchra* (**A**), *Hofstenia miamia* (**B**), and *Convolutriloba macropyga* (**C**). The neuronal markers *choline acetyltransferase* (*ChAT*), *vesicular acetylcholine transporter* (*VAchT*), *vesicular monoamine transporter* (*VMAT*), *synaptotagmin* (*Syn*), *tyrosine hydroxylase* (*TH*), *tryptophan hydroxylase* (*TpH*), *glutamate decarboxylase* (*Gad-1*), *phenylalanine hydroxylase* (*PH*), and the transcription factor *sine-oculis like 3/6* (*Six3/6*) are mostly expressed in the neuronal ganglia and nerve cords comparable to *ASIC* expression in these species. Arrowheads (black in A, yellow in B) highlight *acid-sensing ion channel* (*ASIC)* expression. Nuclei are stained blue with DAPI. Yellow dashed frame images are magnifications of the yellow dashed line boxes. Scale bar: 50 µm. ant, anterior; dor, dorsal.

### Xenacoelomorph ASICs mediate excitatory currents gated by high proton concentrations

When ASICs are exposed to drops in extracellular pH they typically show a transient depolarizing current (inward flow of Na^+^ ions), rapidly followed by either desensitization—a non-ion-conducting state in the presence of agonist—or a smaller sustained current (Krishtal, 2015). To test the function of xenacoelomorph ASICs, we injected the ASIC cRNAs into *Xenopus laevis* oocytes and measured membrane current in response to decreasing pH using two-electrode voltage clamp. At oocytes expressing *I. pulchra* ASIC, drops to pH 5.6 and lower rapidly activated a transient current (Figure 3A, mid-right). However, compared to other ASICs, responses to increased proton concentrations were relatively inconsistent at *I. pulchra* ASIC: although transient currents were never activated by pH higher than 5.9, the concentration dependence of the transient current between pH 5.6 and 4.0 was inconsistent and at lower pH was usually followed by a sustained current ~25% the amplitude of the transient current (Table 1). To verify that these variable currents were indeed specific to the heterologous *I. pulchra* channels, we also applied pH 4.0 to oocytes from the same batch injected with water (Figure 3A, mid-right) or with a proton-insensitive channel (HyNaC2/4/6; Figure 2—figure supplement 1), and we did not observe pH 4.0-gated currents (Figure 2—figure supplement 1). Thus, *I. pulchra* ASIC is indeed gated by protons, but with relatively low potency (pH for half-maximal activation of transient current (pH_50_)=4.9 ± 0.2, Table 1). The *H. miamia* ASIC OQ259911.1 also formed homomeric proton-gated channels, with rapid transient currents followed by smaller sustained currents (18% the amplitude of transient current) in response to drops to pH 5.6 through 4.0 (pH_50_=4.8 ± 0.1, Figure 3B and D, Table 1). The presence of another ASIC gene in this species, 9021040 in Figure 2, raises the possibility of heteromeric channels in Xenacoelomorpha, but we have not pursued that here. Finally, we observed no proton-gated currents in *Xenopus* oocytes injected with *C. macropyga* ASIC cRNA (n=6), suggesting either low heterologous expression or proton insensitivity of this channel.

**Table 1.** Biophysical characteristics of bilaterian acid-sensing ion channels (ASICs) (mean ± SEM). Table 1—source data 1.Numerical data contributing to Table 1 that is not already in Figure Source Data files.

					Proton sensitivity										Permeability	
Animal	I_max_ (Low-Ca^2+^; µA)	n	I_max_ (µA)	n	pH50_a_ (Low-Ca^2+^)	n	pH50_a_	n	pH50_SSD_	n	T_50%_(s)*	n	I_sus_/I_tra_†	n	P_Na+_/P_K+_	n
Xenacoelomorph																
*Isodiametra pulchra*	3.6±0.8	5	0.8±0.2	7	5.3±0.2	5	4.9±0.2	8	7.4±0.1	5	0.09±0.02	6	0.25±0.04	6	8.3±2.0 ≥8‡^,§^	7 6‡
*Hofstenia miamia*	46.1±11.6	5	7.3±1.5	6	5.6±0.1	4	4.8±0.1	6	6.1±0.1	6	0.18±0.02	6	0.18±0.03	6	30.1±5.9 27.7±6.2‡	5 4‡
Spiralian																
*Lingula anatina*	32.1±4.8	6	11.5±1.9	5	7.5±0.01	6	7.3±0.2	6	7.7±0.03	4	8.96±0.42	5	0.02±0.01	5	9.0±1.7	8
*Phoronopsis harmeri*	10.4±0.6	5	7.0±1.3	10	5.7±0.3	5	5.2±0.1	10	6.5±0.05	5	0.22±0.02	4	0.03±0.01	4	3.0±1.0	5
*Owenia fusiformis*	56.6±11.1	6	21.3±2.7	7	8.3±0.1	6	8.1±0.1	5	8.6±0.02	5	0.15±0.01	4	0.00±0.00	4	12.0±0.3	6
Hemichordate																
*Schizocardium californicum*	25.8±3.3	6	7.9±2.0	3	6.05±0.3	6	5.3±0.1	3	7.4±0.17	4	0.18±0.02	6	0.01±0.00	6	2.9±0.5	5

* Time to half-maximal desensitization (half the time from peak transient current to plateaued, sustained current).

† Relation between current amplitudes for sustained and transient peaks (I_sus_/I_tra_).

‡ Values for the sustained current.

§ For *I. pulchra* ASIC, sustained current with extracellular NaCl clearly reversed at 60 mV, and sustained currents with extracellular KCl were small, generally outward, and difficult to quantify. Together this leads us to conclude that P_Na+_/P_K+_ is high.

Like vertebrate ASICs, *I. pulchra* and *H. miamia* ASICs showed decreased responses to activating pH (4.0) after pre-incubation in slightly decreased pH (Figure 3D, open symbols, Table 1), indicating that steady-state desensitization is a broadly conserved phenomenon. Similarly, xenacoelomorph ASICs share the property of inhibition by extracellular calcium with vertebrate ASICs. *I. pulchra* and *H. miamia* ASICs showed 0.4 and 0.8 unit increases in pH_50_ (increased potency) and four- to sixfold increases in current amplitude when the extracellular calcium concentration was reduced from 1.8 to 0.1 mM (Table 1).

Most ASICs so far studied have a slight preference for sodium over potassium ions, with a relative sodium/potassium ion permeability (P_Na+_/P_K+_) of ~10, and thus activation of ASICs leads to depolarization and generation of action potentials in mouse neurons (Gruol et al., 1980). *I. pulchra* ASIC (P_Na+_/P_K+_=8.3 ± 2.0) showed similar Na^+^ selectivity to most ASICs (Figure 3A, lower right and Table 1), whereas *H. miamia* Na^+^ selectivity (P_Na+_/P_K+_=30 ± 5.9; Figure 3B) is remarkably high compared to most ASICs and is more reminiscent of the high preference for sodium seen in ENaCs (Gründer and Pusch, 2015). For both acoel ASICs, the relative ion permeability was similar for transient and sustained currents (Table 1). Since pore-lining and other amino acid residues implicated in ion selectivity in vertebrate ASICs are conserved in xenacoelomorph ASICs, the higher Na^+^ preference of *H. miamia* ASIC must derive from yet unidentified determinants (Figure 2—figure supplement 2).We conclude, however, that both in xenacoelomorphs, which belong to the putative sister group to all other bilaterians, and in vertebrates such as rodents and humans, ASICs are excitatory receptors for protons that are expressed highly in both the brain and in the periphery.

### ASICs are expressed in peripheral neurons and the digestive system in Spiralia

Excitatory proton-gated currents and combined central and peripheral expression of xenacoelomorph ASICs is reminiscent of vertebrate ASICs. However, whether the ancestral bilaterian ASIC performed similar roles remains unclear, as the position of Xenacoelomorpha as sister taxon of all other bilaterians or as part of a group with Ambulacraria within Deuterostomia is debated (Cannon et al., 2016; Kapli et al., 2021; Laumer et al., 2019; Philippe et al., 2019; Figure 1B). We therefore turned to the third major lineage of bilaterians and investigated previously unidentified ASICs in Protostomia. We investigated the expression and function of ASICs in brachiopod, phoronid, and annelid larvae, allowing us to study whole-animal gene expression in animals with typical spiralian features such as an anterior brain or apical organ and ventrolateral or peripherally extending nerve cords.

**Figure 4.**
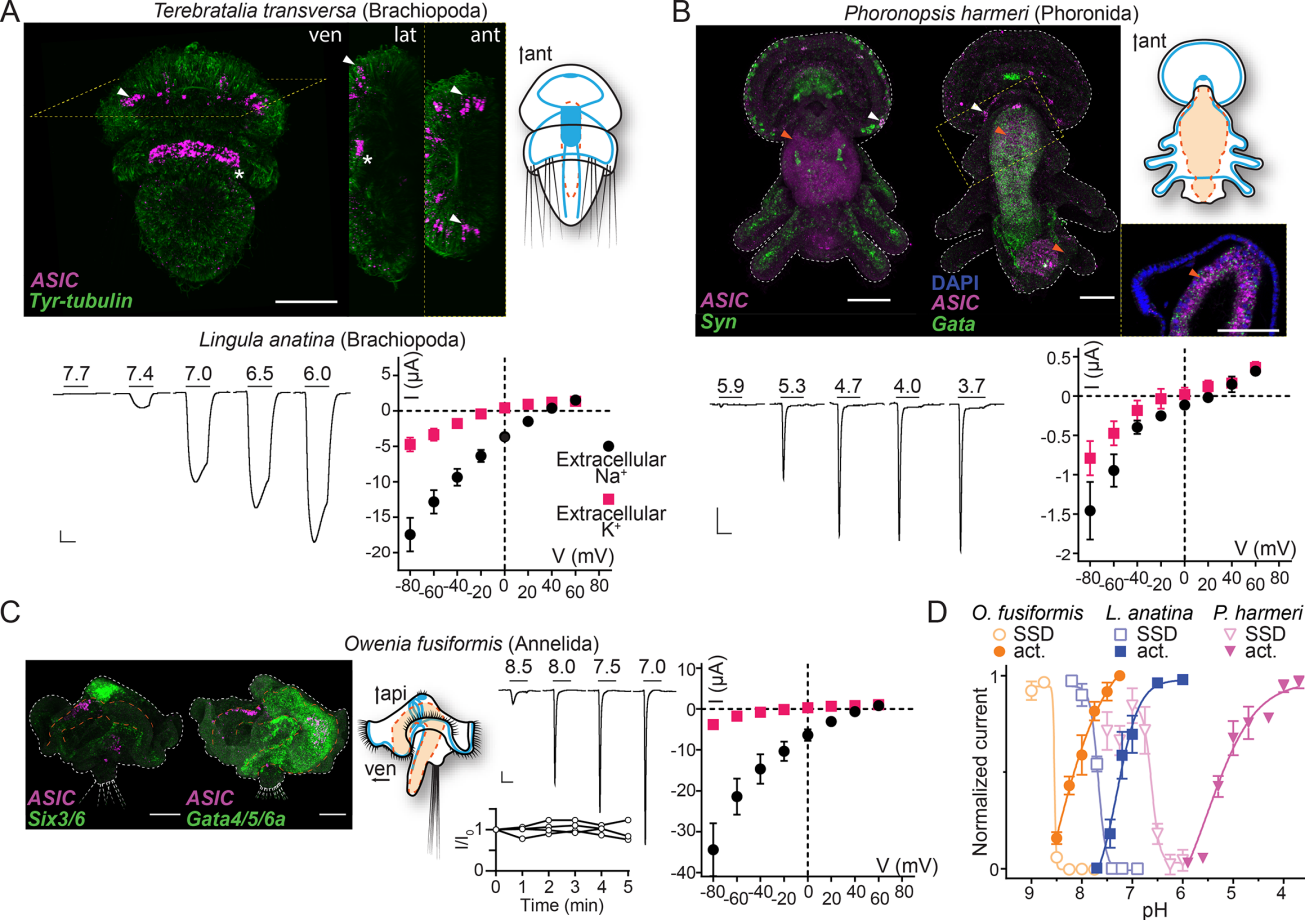
Expression and function of acid-sensing ion channels (ASICs) in Spiralia. (**A,B**) Upper left: ASIC mRNA expression (magenta) and the neuronal (*synaptotagmin*, *Syn; sine-oculis like 3/6, Six3/6*; in green) and endodermal markers (*Gata*, in green). Tyrosinated tubulin immunoreactivity (green) (ant, anterior; api, apical; lat, lateral; ven, ventral; scale bar: 40 µm). Arrowheads, ASIC expression; asterisks, unspecific staining common in *Terebratalia transversa* (Martín-Durán et al., 2018). Yellow dashed frame images are magnifications of the yellow dashed line boxes. Upper right: Cartoons illustrating nervous system (blue) and digestive system (orange; Anlage only in *T. transversa*) after Gąsiorowski et al., 2021; Helm et al., 2016. Lower left: Proton-gated currents in *Xenopus laevis* oocytes expressing indicated spiralian ASICs (scale bars: x, 5 s; y, 1 μA). Lower right: Mean (± SEM) pH 6.5- (*Lingula anatina*) or 4- (*Phoronopsis harmeri* ASIC) gated current (I, μA) at different membrane potentials (V, mV) in the presence of 96 mM extracellular NaCl or KCl (n=5–8). Reversal potential (V_rev_) was read off these plots and the difference between V_rev,NaCl_ and V_rev,KCl_ was used to calculate relative ion permeability (P_Na+_/P_K+_). (**C**) Left to right: ASIC expression, animal morphology, proton-gated currents (top), and normalized current amplitude, that is, amplitude of the current at certain time (-I) divided by the current amplitude at the start of the experiment (I_0_), in response to same proton concentration (pH 7.0) in oocytes perfused with pH 9.0 solution for 5 min (bottom), lines connect data from individual oocytes, and pH 6.5-gated current at different membrane potentials, as in (**A,B**) at *Owenia fusiformis* ASIC. (**D**) Filled symbols: Mean (± SEM) normalized current amplitude in response to increasing proton concentrations (activation, ‘act.’, n=6–10). Open symbols: Mean (± SEM) normalized current amplitude in response to pH 7 for *O. fusiformis*, 6.5 for *L. anatina*, and 4 for *P. harmeri* ASIC following pre-incubation in decreasing pH (steady-state desensitization, ‘SSD’, n=4–5). Figure 4—source data 1.Numerical data contributing to Figure 4.

In the brachiopod *T. transversa*, ASIC was expressed most highly in cells at the lateral edge of the central neuropil in late larvae (Figure 4A). These cells are slightly lateral/caudal to previously identified sensory neurons in the *T. transversa* apical organ (Santagata et al., 2012), and additional in situ hybridization targeting distinct neuronal types showed that the ASIC-expressing cells are close to or overlap with cholinergic neurons at the lateral edge of the central neuropil (Figure 4—figure supplement 1A). Confocal images suggest that these ASIC-expressing cells project latero-anteriorly (Figure 4A, lateral view), perhaps indicative of an anterior sensory role. In the phoronid *P. harmeri*, ASIC was expressed in two principal domains. There was a clear signal colocalizing with *synaptotagmin*-expressing cells in the perimeter of the hood (Figure 4B, white arrowheads), a domain innervated by the peripheral nerve ring and controlling swimming by ciliary beating (Marinković et al., 2020; Temereva and Tsitrin, 2014a). In stark contrast to *synaptotagmin*, ASIC was not expressed in the more central parts of the nervous system, including both the apical organ, an anterior group of sensory neurons that gives way to the developing brain, and the main nerve ring that runs caudally from the apical organ through the trunk and innervates the tentacles (Figure 4—figure supplement 1B; Nielsen, 2015; Temereva and Tsitrin, 2014a; Temereva and Tsitrin, 2014b). The second domain, in which expression was even more prominent, was the digestive system, where ASIC colocalized with the endodermal marker *Gata*. Here, the ASIC expression was high in numerous cells around the digestive tract (comprising a stomach in the trunk and intestine in the pedicle at this stage), most noticeably just below the pharynx and around the lower half of the stomach (Figure 4B, orange arrowheads and inset, Andrikou et al., 2019a). We also detected digestive system expression in the more distantly related spiralian, the annelid *O. fusiformis*. In 19 days post fertilization *O. fusiformis*, we observed no ASIC expression in the young nervous system consisting of a dorsal apical organ already expressing the neuronal marker *six3/6* (Figure 4C), ventral prototroch (or ciliary band) neurons, and anterior and lateral connecting neurons (Carrillo-Baltodano et al., 2021; Helm et al., 2016). Instead, ASIC was clearly expressed in cells around the oesophagus (Figure 4C). In the lumen of the midgut some extracellular signal appeared repeatedly (Figure 4C). These results show that in various spiralians, ASICs are found in the periphery and the digestive system, not in the brain or apical organ.

**Figure 4—figure supplement 1.**
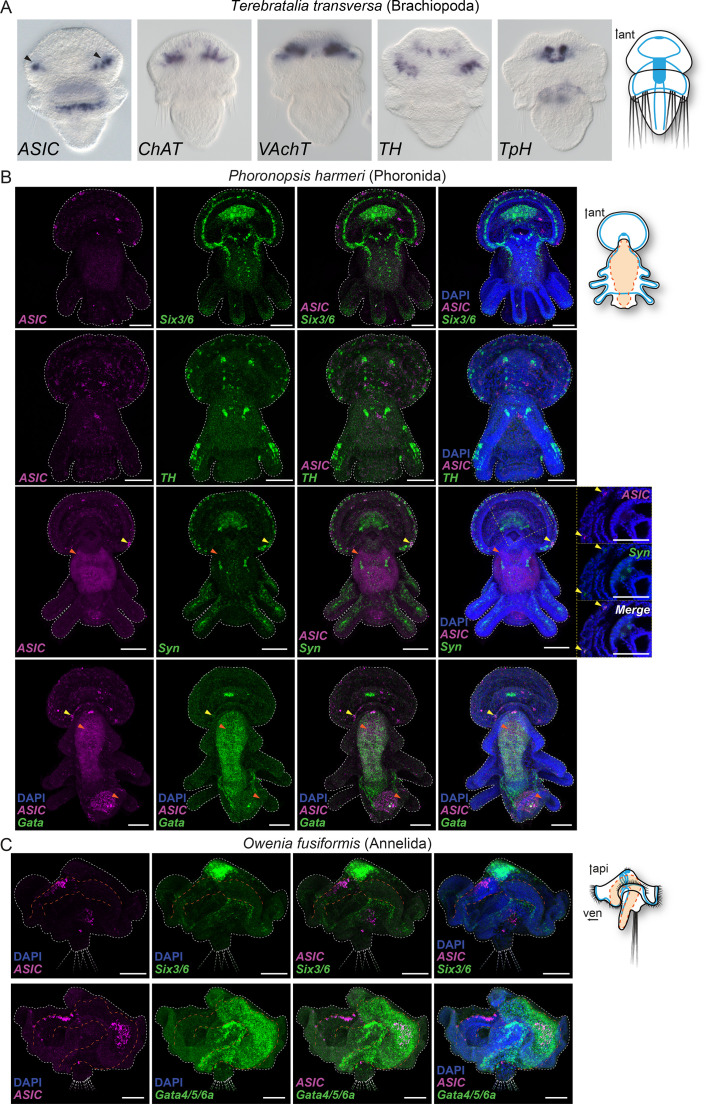
Expression of neuronal and endodermal markers in Spiralia: *Terebratalia transversa* (**A**), *Phoronopsis harmeri* (**B**), and *Owenia fusiformis* (**C**). The neuronal markers *choline acetyltransferase* (*ChAT*), *vesicular acetylcholine transporter* (*VAchT*), *tyrosine hydroxylase* (*TH*), *tryptophan hydroxylase* (*TpH*), *synaptotagmin* (*Syn*), and the transcription factor *sine-oculis like 3/6* (*Six3/6*) are mostly expressed in the neuronal ganglia and nerve cords comparable to *ASIC* expression in these species, whereas the transcription factor *Gata* is expressed in the digestive system. Colocalization of two genes expression is visually identified in the regions of the image that appear white. Arrowheads highlight ASIC expression in nervous system (black in A, yellow in B and C) and in the digestive system (orange). ant. anterior; api, apical; ven, ventral. Yellow dashed frame images are magnifications of the yellow dashed line boxes.

### Spiralian ASICs show a wide range of proton sensitivity and ion selectivity

We next tested the electrophysiological properties of spiralian ASICs by expressing them heterologously in *Xenopus* oocytes. We observed no proton-activated currents in *Xenopus* oocytes injected with *T. transversa* (brachiopod) ASIC RNA (n=10), and we cannot conclude if this is due to low heterologous expression or unknown function of this channel. However, we identified an ASIC transcript in another brachiopod, *L. anatina* (Luo et al., 2015), and observed functional expression of this channel. *L. anatina* ASIC was sensitive to relatively low proton concentrations, with large currents in response to pH 7.4 and lower that enter desensitization slower than most ASICs (Figure 4A, pH_50_=7.3 ± 0.2, time to 50% current amplitude (T_50%_)=8.96 ± 0.42 s, Table 1) and showed typical Na^+^/K^+^ selectivity (Figure 4A, P_Na+_/P_K+_=9.0 ± 1.7). *L. anatina* ASIC has a cysteine residue in contrast to an asparagine residue at position 414 (rat ASIC1a numbering) of vertebrate ASICs (brown in Figure 2—figure supplement 2A). This cysteine residue might contribute to the slow desensitization of *L. anatina* ASIC, as the N414C mutation in human ASIC1a (rat ASIC1a numbering) slows desensitization slightly and its chemical modification slows desensitization greatly (Roy et al., 2013).

The phoronid *P. harmeri* ASIC showed much lower proton sensitivity, activated by pH in the range of 5.9–3.7, with rapidly desensitizing currents (Figure 4B, pH_50_=5.2 ± 0.1, Table 1) and almost no preference for sodium over potassium ions (Figure 4B, P_Na+_/P_K+_=3.0 ± 0.9, Table 1). We next tested the function of ASIC from the annelid *O. fusiformis*. This channel was extremely sensitive to low proton concentrations. When held at pH 7.5 and exposed to lower pH, *O. fusiformis* ASIC showed very small current responses, but when held at pH 9.0 and exposed to small drops in pH, large inward currents were activated that rapidly desensitized (Figure 4C, pH_50_=8.1 ± 0.1, Table 1). We measured repeated responses of *O. fusiformis* ASIC to pH 7.0 over the course of 5 min in pH 9.0 and observed no decrease in current amplitude (Figure 4C), verifying that the relatively basic resting pH of 9.0 was not harming oocytes and skewing results with this channel. *O. fusiformis* ASIC also showed ion selectivity typical of most ASICs (Figure 4C, P_Na+_/P_K+_=12.0 ± 0.3). We also found that reducing the extracellular calcium concentration from 1.8 to 0.1 mM increased the pH_50_ and increased current amplitude at *O. fusiformis* ASIC (Table 1), indicating the extreme proton sensitivity of this channel is not due to an absence of inhibition by extracellular calcium. *P. harmeri* and *L. anatina* ASICs were also inhibited by extracellular calcium, with 0.2 to 0.5 unit increases in pH_50_ and two- to threefold increases in current amplitude in 0.1 mM extracellular calcium relative to 1.8 mM (Table 1).

Spiralian ASICs seem to vary in apparent proton affinity and in ion selectivity among the lineages, from low proton sensitivity (pH ~5) and essentially non-selective cation currents in phoronid ASIC to high proton sensitivity (pH 7–8) and ~10-fold selective sodium permeability in brachiopod and annelid ASICs. All showed canonical steady-state desensitization, occurring in pH ranges higher than activating pH (Figure 4D and Table 1). Spiralian ASIC genes thus encode proton-gated cation channels, and ASICs are present in all major groups of bilaterians: Xenacoelomorpha, Protostomia, and Deuterostomia.

### Hemichordate ASIC is expressed in peripheral cells and pharynx and mediates rapidly desensitizing excitatory currents in response to protons

**Figure 5.**
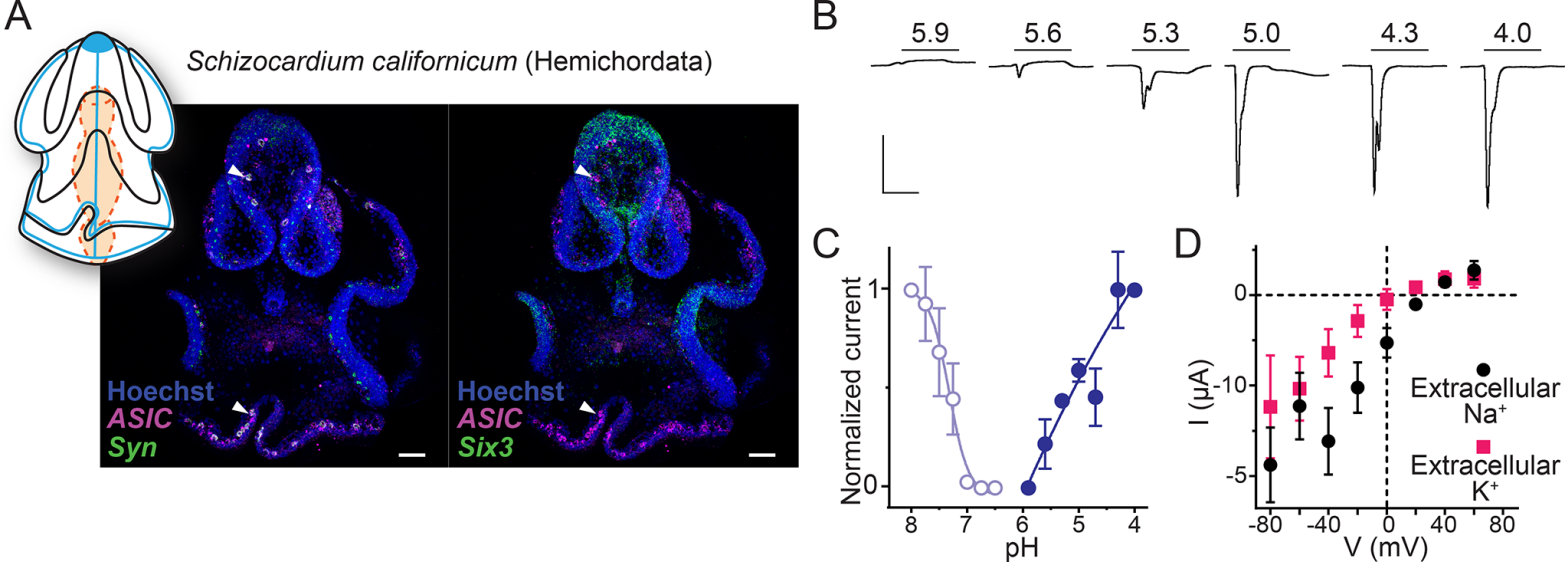
Expression and function of hemichordate acid-sensing ion channel (ASIC). (**A**) Cartoon illustrating nervous system (blue) and digestive system (orange) in *Schizocardium californicum*, after Gonzalez et al., 2017, and *hybridization chain reaction* (HCR) showing expression of *S. californicum* ASIC and the neuronal markers *synaptotagmin* (Syn) or *sine-oculis like* (*Six3*). White arrowheads highlight ASIC expression. Colocalization of two genes’ expression is visually identified in the regions of the image that appear white. Nuclei are stained blue with Hoescht. Scale bar: 50 μm. (**B**) Proton-gated currents in *Xenopus laevis* oocytes expressing *S. californicum* ASIC. Scale bars: x, 5 s; y, 2 μA. (**C**) Filled symbols: Mean (± SEM) normalized current amplitude in response to increasing proton concentrations (activation, ‘act.’, n=3). Open symbols: Mean (± SEM) normalized current amplitude in response to pH 4 following pre-incubation in decreasing pH (steady-state desensitization, n=4). (**D**) Mean (± SEM) pH 4-gated current (I, μA) at different membrane potentials (V, mV) in the presence of 96 mM extracellular NaCl or KCl (n=5). Figure 5—source data 1.Numerical data contributing to Figure 5.

Regarding Deuterostomia, combined expression and function of ASICs have so far only been characterized in selected chordates (urochordates and vertebrates) (Coric et al., 2008; Kellenberger et al., 2015; Lynagh et al., 2018), and relatively little is known about ASICs in the ambulacrarian lineage (echinoderms and hemichordates). Recent studies on neural development in sea urchin larvae (*Lytechinus variegatus*, an echinoderm) showed that ASIC is expressed diffusely throughout the ciliary band (Slota et al., 2020). This transcript (MH996684) is closely related to the Group B ASICs from echinoderms and hemichordates that showed proton-gated currents when expressed heterologously (Lynagh et al., 2018). Here, we used the hemichordate *S. californicum* to characterize the combined expression and function of an ambulacrarian ASIC. *S. californicum* ASIC expression was visible in late larval stages when structures such as the nervous system and the ciliary bands become more intricate, like sea urchin ASIC (Slota et al., 2020). The tornaria larva of *S. californicum* has two main ciliary bands: the circumoral band for feeding, and the telotroch, innervated by serotonergic neurites, for locomotion (Gonzalez et al., 2017). We performed numerous ASIC in situs in *S. californicum* larvae and observed variable expression patterns between the colorimetric and fluorescent in situ (Figure 5—figure supplement 1). Expression in the ciliary band was consistent but there was also signal in other tissues such as the pharynx that varied across samples (Figure 5—figure supplement 1A). To resolve these inconsistencies, we turned to HCR (*hybridization chain reaction*, Choi et al., 2018) to better resolve the expression pattern. By doing this we were able to resolve the variability of the ASIC in situ across experiments as well as specificity of the expression pattern by including the markers synapotagmin which marks neural cells (Nakajima et al., 2004) and *Six3* which marks the most anterior territory including the apical organ in *S. californicum* (Gonzalez et al., 2017). With these triple HCRs, we found ASIC expressed in some of the *synaptotagmin* cells (Figure 5A, white arrowhead, and Figure 5—figure supplement 1B–E) but excluded from the most anterior, apical organ, *Six3*-positive territory.

**Figure 5—figure supplement 1.**
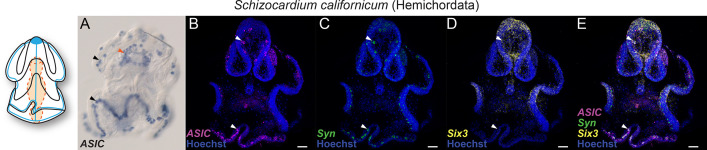
Expression of acid-sensing ion channel (ASIC) and neuronal markers in Hemichordata: Colorimetric in situ hybridization for ASIC (**A**) and *hybridization chain reactions* (HCRs) for *ASIC* (**B**), *synaptotagmin* (Syn) (**C**), *sine-oculis like* (*Six3*) (**D**), and the merge (**E**). Arrowheads (black in A, white in B to E) highlight ASIC expression. Colocalization of two genes expression is visually identified in the regions of the image that appear white. Nuclei are stained blue with Hoescht. Scale bar: 50 µm.

When heterologously expressed, *S. californicum* ASIC showed rapidly activating and desensitizing currents in response to extracellular acidification with proton sensitivity comparable to a previously described hemichordate ASIC and lower than echinoderm ASICs (Figure 5B, pH_50_=5.3 ± 0.1) (Lynagh et al., 2018). The *S. californicum* channel showed relatively weak ion selectivity, with P_Na+_/P_K+_ values slightly above unity (2.9±0.5; Figure 5D and Table 1), similar to ion selectivity in previously described ambulacrarian ASICs (Lynagh et al., 2018). Finally, current amplitude and proton potency were both inhibited by extracellular calcium, with 0.7 unit increased pH_50_ and fourfold larger currents in 0.1 mM calcium compared to 1.8 mM calcium (Table 1). *S. californicum* thus presents a functional ASIC that is expressed in the peripheral nervous system and the digestive system, and it appears that such peripheral expression of ASICs is a conserved feature of Deuterostomia, Protostomia, and Xenacoelomorpha.

## Discussion

### The emergence of ASICs

Our results show that ASICs—of the same family as the prototypical rat ASIC1a—are present in Xenacoelomorpha and Protostomia in addition to Deuterostomia and are thus conserved in the three major groups of Bilateria. We also find, consistent with previous studies, that ASICs of this family are absent from the other major lineages of Cnidaria, Placozoa, Porifera, and Ctenophora, indicating that ASICs emerged in the lineage to the Bilateria, soon after the Cnidaria/Bilateria split ~680 Mya (Kumar et al., 2017). The clades most closely related to ASICs within the DEG/ENaC superfamily tree include three from which several channels have been characterized: mammalian bile acid-sensitive ion channels (BASICs), *Trichoplax adhaerans* Na^+^ channels (TadNaCs); and HyNaC and *Nematostella vectensis* Na^+^ channels (NeNaCs). We cannot establish phylogenetically the identity of the ancestral gene from which ASICs emerged, as branch support toward the base of the ASIC + BASIC + TadNaC + HyNaC/NeNaC clade is relatively low: our maximum likelihood (ML) trees inferred with aLRT SH-like and aBayes statistics yielded slightly different topologies within this clade (Figure 2—figure supplement 1C and D), and previous studies using similar methods to each other also inferred slightly different topologies within this clade (Aguilar-Camacho et al., 2022; Elkhatib et al., 2022). Statistical support for the distinct ASIC clade is strong, however (Figure 2—figure supplement 1).

We are thus left to consider the *functional* relationships among these cousins. BASICs, highly expressed in mammal intestines, are activated by bile acids or are constitutively active, and show inhibition by protons (Wiemuth et al., 2014; Wiemuth and Gründer, 2010). Non-mammalian genes from the BASIC clade have not been characterized. HyNaCs are neuropeptide-gated channels from the medusozoan cnidarian *Hydra* (Assmann et al., 2014), and when NeNaCs from the anthozoan cnidarian *Nematostella* were recently reported, surprisingly, NeNaC2 and NeNaC14 were activated by protons (pH_50_ values of 5.8 and <4.0), not by cnidarian neuropeptides (Aguilar-Camacho et al., 2022). The TadNaC clade also includes a proton-activated channel, TadNaC2 (pH_50_ 5.1), in addition to a proton-inhibited channel, TadNaC6 (Elkhatib et al., 2019; Elkhatib et al., 2022). Thus, proton-activated and -inhibited channels occur sporadically throughout the ASIC + BASIC + TadNaC + HyNaC/NeNaC clade, and only in the ASIC clade is proton-induced activation the defining feature. ASICs could thus have emerged from a proton-activated ancestor, after which this function was selected for; or from a peptide- or bile acid-activated ancestor, in which a novel mechanism of activation evolved. Certain functional and phylogenetic data have led others to favor the latter possibility (Elkhatib et al., 2022; Golubovic et al., 2007), and indeed structural features important for channel gating throughout the prototypical ASIC family, such as highly conserved histidine residues and/or aromatic interactions, are absent from proton-gated NeNaCs and TadNaCs (Elkhatib et al., 2022; Lynagh et al., 2018). The fact that proton-induced activation has emerged in numerous DEG/ENaC channels, even outside the ASIC + BASIC + TadNaC + HyNaC/NeNaC clade, as shown for a *Drosophila* PPK channel and three *C. elegans* degenerin-like channels (Jang et al., 2019; Kaulich et al., 2022), suggests a propensity for this function in the DEG/ENaC superfamily.

**Figure 6.**
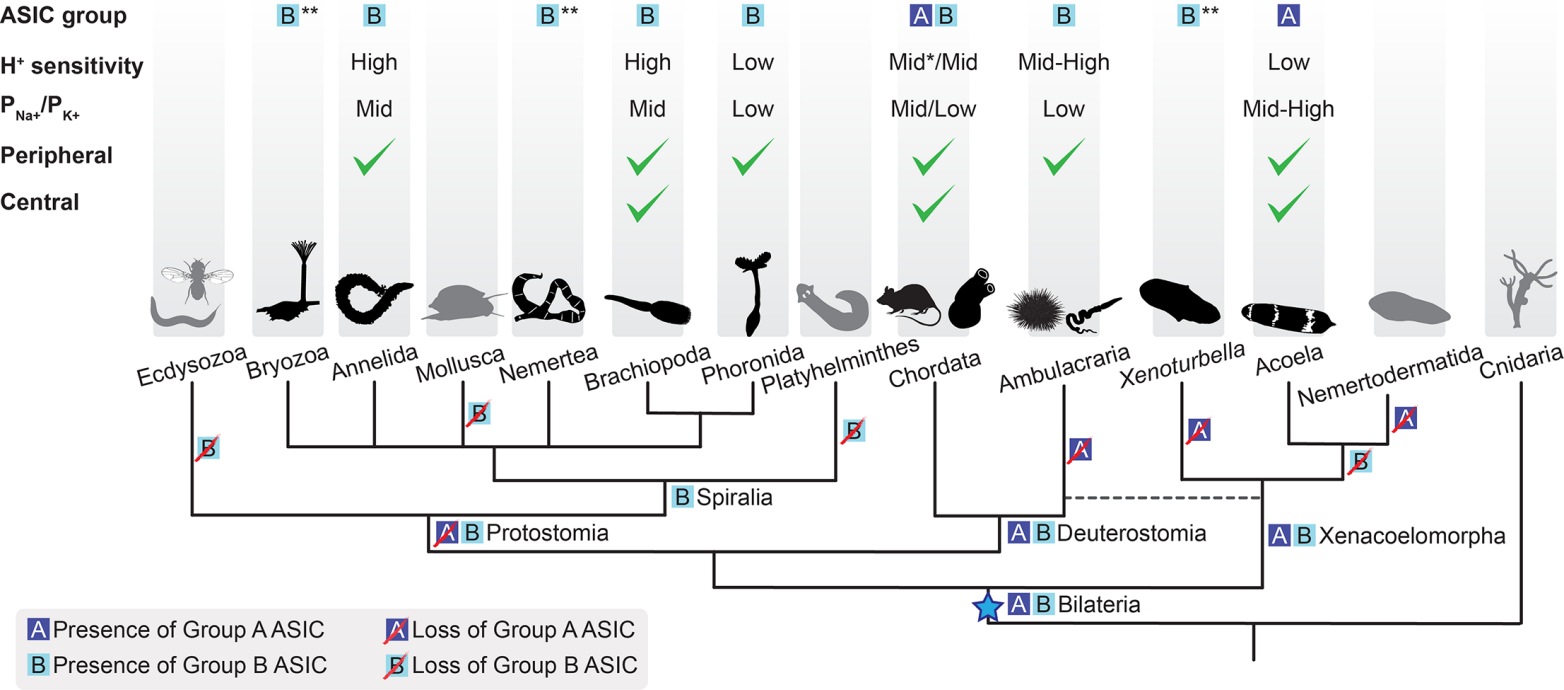
Evolutionary history of acid-sensing ion channel (ASIC) function in Metazoa. Upper half of diagram indicates characteristic properties of ASIC in different lineages: degenerin/epithelial sodium channel (DEG/ENaC) gene tree position (‘ASIC group A or B’); proton (H^+^) sensitivity; relative ion permeability (‘P_Na+_/P_K+_’); native expression pattern (‘Peripheral’, ciliary or gastrointestinal epithelia and/or peripheral neurons; ‘Central’, brain and/or nerve cords). Lower half shows phylogenetic relationships of the different animal phyla studied here. Blue star, putative emergence of ASICs in the last common ancestor of all bilaterians after Cnidaria/Bilateria split. For H^+^ sensitivity, ‘low’, ‘mid’, and ‘high’ correspond to pH_50_ <5.3, 5.3–7, and >7, respectively. *, a minority of characterized chordate ASICs have low H^+^ sensitivity (Gründer and Pusch, 2015; Paukert et al., 2004). For relative ion permeability, ‘low’, ‘mid’, and ‘high’ correspond to P_Na+_/P_K+_≤3, 3–15, and >15, respectively. **, channel function not experimentally tested.

Soon after its emergence in an early bilaterian, we infer that ASIC duplicated, giving rise to two similar proton-gated channels, based on Group A and Group B ASICs in our phylogeny. Subsequently, and at different times, most descendants lost one of these (Figure 6). Acoels lost Group B ASIC and xenoturbellans lost Group A ASIC soon after this early split within the Xenacoelomorpha. In contrast, the first deuterostomes and, even more recently, the first chordates likely retained both ASICs, reflected in the continued presence of both in Cephalochordata (Figure 2, Figure 6). Subsequently, in Olfactores (Craniata + Urochordata), Group B ASIC was lost and Group A ASIC underwent independent radiations, leading to, for example, ASIC1-ASIC4 paralogues in vertebrates (Paukert et al., 2004), two Group A ASICs in urochordates (Lynagh et al., 2018), and multiple Group A ASICs in cephalochordates (Figure 2).

### Peripheral and central roles for ASICs

By investigating ASIC gene expression in each of the three major bilaterian lineages, we see that ASICs are principally found: centrally, in the brain; and/or peripherally, in the digestive system, peripheral nerve rings, and/or scattered peripheral cells. This, together with previous research on ASICs in rodents, points to at least two functions of ASICs: a central signaling role, where ASICs mediate rapid excitatory signals between neurons in the brain (Du et al., 2014) and a peripheral sensory/modulatory role, where ASICs convert increased proton concentrations in the environment—extra-organismal or in the gut—into excitatory neuronal signals (Krishtal and Pidoplichko, 1981). Brain expression of mouse, zebrafish, and urochordate ASICs suggests conservation of the synaptic role in various chordates (Du et al., 2014; González-Inchauspe et al., 2017; Lynagh et al., 2018; Paukert et al., 2004), whereas in the other deuterostome lineage of Ambulacraria (Figure 1B), nervous systems are less centralized and ASIC expression appears more peripheral (Slota et al., 2020 and Figure 6). In Xenacoelomorpha, based on the markers we used, we could not assign ASIC expression to a particular type of neuron, except in *I. pulchra and H. miamia*, where some of the ASIC-expressing cells were cholinergic and GABAergic, respectively (Figure 3—figure supplement 1A and B). In contrast, central ASICs were observed in only one of the three Spiralia tested, and even these ASIC-expressing cells showed projections toward the very anterior of the animal, perhaps indicative of a less integrative and more sensory role (Figure 4A). Brain ASICs are thus common to Xenacoelomorpha and Deuterostomia and are less prevalent in Protostomia, and this central role is more commonly played by Group A ASICs than Group B ASICs (Figure 6).

The second, peripheral domain, observed in the seven species of Xenacoelomorpha, Spiralia, and Deuterostomia studied here, largely corresponds to the ciliation in the species. While xenacoelomorphs are completely covered with ciliated epithelial cells, in many other Bilateria the ciliation is restricted to ciliary bands or the ciliary ventral region, both used for locomotion. These ASICs are thus expressed in a location to sense external changes in pH and directly modulate ciliary movement via excitatory current or, via depolarization and synaptic release onto more central neurons, send the information centrally. Conversely, if these ASICs are expressed on the proximal side of these peripheral cells, their role may be modulation of ciliary function in response to efferent modulatory signals. Although activation of postsynaptic ASICs has so far been linked to only glutamatergic and GABAergic synapses (Du et al., 2014; Storozhuk et al., 2016) and innervation of ciliary cells appears monoaminergic in Spiralia (Marinković et al., 2020), monoaminergic vesicles are also acidic and could thus foreseeably release protons to activate ASICs here (Onoa et al., 2010).

We were surprised to see expression of ASIC predominantly in the digestive system of the spiralians *O. fusiformis* and *P. harmeri*. In mammals, chemosensors such as ASIC and transient receptor potential channels (e.g. TRPV1) are expressed in sensory neurons innervating the gastrointestinal tract (Holzer, 2015), but oesophageal, intestinal, and lung epithelial cells also express ASICs, potentially contributing to acid-induced secretions, transport, and inflammation (Dong et al., 2011; Su et al., 2006; Ustaoglu et al., 2021). The gastrointestinal expression of ASICs that we observed may reflect a chemosensory role, like that mediated by ASICs in neurons innervating the lungs and skin of mammals (Diochot et al., 2012; Gu and Lee, 2006). Similar functions are tentatively suggested by ASIC expression in the peripheral nervous system of Ambulacraria (Slota et al., 2020; Figure 5). Again, we can’t exclude the possibility that these peripheral ASICs are modulating ciliary function, either directly via acid-induced activation or indirectly, via efferent signals from central neurons. Nonetheless, these expression patterns seem to suggest that ASICs play a sensory role in the periphery of Deuterostomia, Protostomia, and Xenacoelomorpha via expression in cells that correlate with ciliation, indicating that such a role was probably present in the ancestral bilaterian. Consistent with the early appearance of this role, it is played by both Group A and Group B ASICs, which both emerged in early bilaterians. Subsequently, ASICs—particularly Group A—were likely deployed to the other, more central role for inter-neuronal communication in lineages such as Acoela and Chordata.

### Loss of ASICs

The conservation and high expression of ASICs in bilaterians begs the question as to why they were lost in certain lineages. The reason for the loss of ASICs in selected Spiralia, such as molluscs and platyhelminthes is unclear, especially when one considers that numerous molluscs are susceptible to the tide and thus vast fluctuations in pH. Presumably, gene radiations that increased sensory ion channel or transporter diversity in such animals have compensated for the loss of ASICs (Albertin et al., 2015; Fu et al., 2021; Simakov et al., 2013; Xun et al., 2020; Zhang et al., 2012), and indeed, certain members of various channel families are capable of mediating excitation (or inhibition) in response to decreased pH (Pattison et al., 2019). Perhaps most noticeable, however, is the absence of ASICs in Ecdysozoa, a broad lineage including pan-arthropods, nematodes, and priapulids, each of which we considered in our phylogenetic analysis. Ecdysozoa split from Spiralia ~650 Mya (Kumar et al., 2017), adopting a chitinous cuticle utilizing rigid locomotory structures and requiring periodic molting for growth (Howard et al., 2020; Schmidt-Rhaesa et al., 1998). Concomitantly, Ecdysozoa lost the ectodermal, motile ciliated cells inherited from the last common ancestor of Cnidaria and Bilateria that mediate locomotory, feeding, secretory, and sensory functions in most other bilaterians (Ringers et al., 2020; Valentine and Collins, 2000). The correlation between loss of ASICs and loss of motile ciliated cells, together with the conservation of other ciliated and sensory cells in Ecdysozoa, indicates that the role of ASICs in early Protostomia was primarily associated with ectodermal ciliated cells rather than more central sensory cells associated with, for example, mouth, eyes, and antennae. This, together with the presence of ASICs in the periphery of Spiralia, Xenacoelomorpha, and Deuterostomia, suggests that the role of the earliest ASIC was likely local conversion of external chemical stimuli into modulation of locomotion, conversion of central signals into modulation of locomotion, or both. Indeed, motile ciliated epithelia in human lungs are modulated by both external and central stimuli (Shah et al., 2009), although the precise contribution of ASICs in these cells is unclear (Su et al., 2006).

### Outlook

We acknowledge that future experiments might benefit from greater genomic resources for non-bilaterian animals, but our comprehensive survey of DEG/ENaC genes from Ctenophora, Porifera, Placozoa, and Cnidaria finds no ASICs in those lineages. Our study suggests that ASICs emerged in an early bilaterian, most likely in peripheral cells or epithelia, and were gradually adopted into the central nervous system of certain complex animals. This offers a unique insight into the employment of LGICs during early bilaterian evolution. Functional characterization of diverse ASICs shows a considerable breadth of pH sensitivity and ion selectivity throughout the family, offering new tools for probing the biophysical mechanisms of function. The combined use of gene expression and experimental analysis is thus a useful tool in understanding protein evolution and function.

## Materials and methods

**Key resources table:** 

Reagent type (species) or resource	Designation	Source or reference	Identifiers	Additional information
gene (*Isodiametra pulchra*)	ASIC	NCBI	OQ259901.1	
gene (*Isodiametra pulchra*)	ChAT	NCBI	KY709762.1	
gene (*Isodiametra pulchra*)	VAchT	NCBI	KY709763.1	
gene (*Isodiametra pulchra*)	VMAT	NCBI	KY709764.1	
gene (*Isodiametra pulchra*)	Syn	NCBI	OQ259900.1	
gene (*Isodiametra pulchra*)	TH	NCBI	KY709765.1	
gene (*Isodiametra pulchra*)	TpH	NCBI	KY709766.1	
gene (*Hofstenia miamia*)	ASIC	NCBI	OQ259911.1	
gene (*Hofstenia miamia*)	Gad-1	NCBI	MT657938.1	
gene (*Hofstenia miamia*)	TpH-1	NCBI	MT657942.1	
gene (*Convolutriloba macropyga*)	ASIC	NCBI	OQ259902.1	
gene (*Convolutriloba macropyga*)	Six3/6	NCBI	OQ259903.1	
gene (*Convolutriloba macropyga*)	TH	NCBI	OQ259904.1	
gene (*Convolutriloba macropyga*)	PH	NCBI	OQ259905.1	
gene (*Terebratalia transversa*)	ASIC	NCBI	OQ259906.1	
gene (*Terebratalia transversa*)	ChAT	NCBI	KY809754.1	
gene (*Terebratalia transversa*)	VAchT	NCBI	KY809753.1	
gene (*Terebratalia transversa*)	TH	NCBI	OQ259907.1	
gene (*Terebratalia transversa*)	TpH	NCBI	KY809752.1	
gene (*Lingula anatina*)	ASIC	OIST	g20471.1	
gene (*Phoronopsis harmeri*)	ASIC	NCBI	OQ259908.1	
gene (*Phoronopsis harmeri*)	Six3/6	NCBI	MN431430.1	
gene (*Phoronopsis harmeri*)	TH	NCBI	OQ259909.1	
gene (*Phoronopsis harmeri*)	Syn	NCBI	OQ259910.1	
gene (*Phoronopsis harmeri*)	Gata4/5/6	NCBI	MN431425.1	
gene (*Owenia fusiformis*)	ASIC	NCBI	OQ259912.1	
gene (*Owenia fusiformis*)	Six3/6	NCBI	KR232531	
gene (*Owenia fusiformis*)	Gata4/5/6 a	NCBI	KR232537	
gene (*Schizocardium californicum*)	ASIC	NCBI	OQ259913.1	
gene (*Schizocardium californicum*)	Syn	NCBI	OQ259914.1	
gene (*Schizocardium californicum*)	Six3	NCBI	KX845335.1	
commercial assay or kit	pGEM-T Easy vector	Promega	A1360	
commercial assay or kit	Ambion Megascript T7	Invitrogen	AM1334	
commercial assay or kit	Ambion Megascript SP6	Invitrogen	AM1330	
commercial assay or kit	TSA Cy3 kit	PerkinElmer	NEL744001KT	
commercial assay or kit	TSA Cy5 kit	PerkinElmer	NEL745001KT	
antibody	Monoclonal Anti-Tubulin, Tyrosine antibody produced in mouse	Sigma-Aldrich	T9028	
antibody	Alexa Fluor 488 goat anti-mouse IgG	Life Technologies	A-11029	
antibody	Anti-Digoxigenin-AP, Fab fragments	Roche	11093274910	
antibody	Anti-Digoxigenin-POD, Fab fragments	Roche	11207733910	
antibody	Anti-DNP HRP Conjugate	Akoya	TS-0004000	
other	DAPI	Molecular Probes	D1306	
sequence-based reagent	Scal_ASIC	Molecular Instruments, Inc	HCR probe	
sequence-based reagent	Scal_Six3	Molecular Instruments, Inc	HCR probe	
sequence-based reagent	Scal_Syn	Integrated DNA Technologies	DNA oligo pool	
other	HCR amplifiers with fluorophores B1-Alexa Fluor-647	Molecular Instruments, Inc		
other	HCR amplifiers with fluorophores B2-Alexa Fluor-488	Molecular Instruments, Inc		
other	HCR amplifiers with fluorophores B3-Alexa Fluor-546	Molecular Instruments, Inc		
recombinant DNA reagent	modified pSP64poly(A) (plasmid)	This paper		contains 5′- and 3′-UTR sequences of the *X. laevis* β-globin gene, and a C-terminal Myc tag
commercial assay or kit	SP6 Polymerase mMessage mMachine kit, Ambion	Fisher	10391175	
biological sample (*Xenopus laevis*)	Oocytes	Ecocyte Bioscience		
other	OC-725C amplifier	Warner Instruments		
other	LIH 8+8 digitizer	HEKA		
software	Patchmaster	HEKA		
software	pClamp v10.7	Molecular Devices		
chemical compound, drug	Sodium ursodeoxycholic acid	Santa Cruz Biotechnology	sc-222407	
peptide, recombinant protein	(pyroE)WLGGRFamide	Genscript		

### Survey and phylogenetic analysis

Mouse ASIC1a was used as a query in tBLASTn searches of DEG/ENaC genes in xenacoelomorphs (*C. macropyga, C. submaculatum, H. miamia, I. pulchra, X. bocki, X. profunda, N. westbladi,* and *M. stichopi* from transcriptomes published in Andrikou et al., 2019b), spiralians (*Spadella* spp*., Dimorphilus gyrociliatus, Epiphanes senta, Lepidodermella squamata, Lineus longissimus, Lineus ruber, M. membranacea, N. anomala, O. fusiformis, P. harmeri, P. vittatus,* and *T. transversa* from our transcriptomes in preparation; *A. granulata, C. gigas*, and *Brachionus plicatilis* from NCBI; *L. anatina* and *N. geniculatus* from OIST; and *S. mediterranea* from SmedGD), ecdysozoans (*H. spinulosus, P. caudatus, P. vulgare* from our transcriptomes; *D. melanogaster, D. pulex, C. sculpturatus,* and *C. elegans* from NCBI) and a hemichordate (*S. californicum,* our transcriptome). Other DEG/ENaC genes were retrieved via BlastP at public databases NCBI, Compagen, JGI, OIST, OikoBase, Aniseed, or UniProt targeting cnidarians (hexacorallian *N. vectensis*, octacorallian *Dendronephthya gigantea*, scyphozoan *Aurelia aurita*, hydrozoan *H. vulgaris*), poriferan (*Amphimedon queenslandica*), the placazoan *Trichoplax adhaerens*, ctenophores (cydippid *Pleurobrachia bachei* and lobate *Mnemiopsis leidyi*), and deuterostomes (chordates *Rattus norvegicus, Ciona robusta, Oikopleura dioica,* and *Branchiostoma belcheri*; hemichordate *Ptychodera flava*; and echinoderm *Acanthaster planci*). Amino acid sequences were aligned using MAFFT (Katoh and Standley, 2013), variable N- and C-termini were removed and highly similar sequences were not considered (Supplementary file 1), and homologies were assigned by phylogenetic tree analyses based on ML inferences calculated with PhyML v3.0 (Guindon et al., 2010). Robustness of tree topologies was assessed under automatic model selection based on Akaike information criteria. Due to computational load of bootstrap performance, trees were inferred using the fast likelihood-based methods: aLRT SH-like; and aBayes (Anisimova et al., 2011). Cell marker genes (Supplementary file 2) were identified similarly, but whereas homology of synaptotagmin, Gata, and Six3 was obvious, homology of PH, TH, and TpH genes was confirmed phylogenetically with reference to earlier work (Siltberg-Liberles et al., 2008).

### Animal collection and fixation

Stable cultures of acoels were maintained in the laboratory. *C. macropyga* (Shannon and Achatz, 2007) were reared in a tropical aquarium system with salinity 34±1 ppt at a constant temperature of 25°C. The aquariums were illuminated (Pacific LED lamp WT470C LED64S/840 PSU WB L1600, Philips) on a day/night cycle of 12/12 hr. The worms were fed with freshly hatched brine shrimp *Artemia* twice per week. *I. pulchra* (Smith and Bush, 1991) were cultured as described by De Mulder et al., 2009, and *H. miamia* (Correa, 1960) as described by Srivastava et al., 2014. For the remaining species, adult gravid animals were collected from Bodega Bay, California, USA (*P. harmeri* Pixell, 1912), San Juan Island, Washington, USA (*T. transversa* Sowerby, 1846), Station Biologique de Roscoff, France (*O. fusiformis* Delle Chiaje, 1841), and Kanangra Boyd National Park and Morro Bay State Park, California, USA (*S. californicum* Cameron and Perez, 2012). Animals were spawned and larvae obtained as described in Freeman, 1993; Gonzalez et al., 2018; Rattenbury, 1954; Wilson, 1932. Adult and larval specimens were starved for 2–7 days prior to fixation. Samples were relaxed in 7.4% magnesium chloride and fixed in 4% paraformaldehyde in culture medium for 1 hr at room temperature and washed several times in 0.1% Tween 20 phosphate buffered saline (PBS), dehydrated through a graded series of methanol, and stored in pure methanol or ethanol at −20°C.

### Cloning

The full-length coding sequences of identified ASIC genes were amplified from cDNA of *C. macropyga*, *I. pulchra*, *H. miamia*, *P. harmeri*, *O. fusiformis*, *T. transversa*, and *S. californicum* by PCR using gene-specific primers. PCR products were purified and cloned into a pGEM-T Easy vector (Promega, A1360) according to the manufacturer’s instructions and the identity of inserts confirmed by sequencing. Riboprobes were synthesized with Ambion Megascript T7 (AM1334) and SP6 (AM1330) kit following the manufacturer’s instruction for subsequent in situ hybridization. Additional cell markers (Supplementary file 2) were cloned similarly, whether full-length or shorter, as desired for in situ hybridization probes (below).

### Immunohistochemistry and situ hybridization

Single whole-mount colorimetric and fluorescent in situ hybridization was performed following an established protocol (Martindale et al., 2004) with probe concentration of 0.1 ng/μl (*I. pulchra*) or 1 ng/μl (the remaining species) and hybridization temperature of 67°C. Proteinase K treatment time was adjusted for each species and ranged from 2 min (*P. harmeri, O. fusiformis, S. californicum*) to 10 min (*T. transversa*). Post-hybridization low salt washes were performed with 0.05× saline sodium citrate (SSC; *H. miamia*) or 0.2× SSC (the remaining species). Fluorescent in situ hybridization was visualized with TSA Cy3 kit (PerkinElmer, NEL752001KT). Samples were mounted in 70% glycerol or subjected to immunohistochemistry for visualization of neural structures: samples were permeabilized in 0.2% Triton X in PBS (PTx) and blocked in 1% bovine serum albumin in PTx (PBT) and incubated with antibodies against tyrosinated tubulin (Sigma, T9028) at a concentration of 1:250 in PTx with 5% normal goat serum, and incubated for 16–18 hr at 4°C. After several washes in PBT the samples were incubated with secondary goat anti-mouse antibodies conjugated with Alexa Fluor 488 (Life Technologies), at a concentration 1:200 in PTx with 5% normal goat serum for 16–18 hr at 4°C, and samples washed extensively before mounting in 70% glycerol and imaging. Nuclei were stained with DAPI (Molecular Probes), unless otherwise indicated. When results were consistent (most animals), in situ hybridization was performed at least twice. For ambiguous results (*S. californicum*), several more rounds were performed, and the most representative results were chosen for display in figures.

### Hybridization chain reaction

HCR in situ hybridization was performed in *Schizocardium* following an established protocol (Bump et al., 2022). For HCR probe design, complementary DNA sequences for *ASIC* and *Six3* were submitted to Molecular Instruments, Inc and for *synaptotagmin*, the Ozpolat Lab HCR probe generator (Kuehn et al., 2022) was used and sequences were ordered as a DNA oligo pool from Integrated DNA Technologies. HCR amplifiers with fluorophores B1-Alexa Fluor-647, B2-Alexa Fluor-488, and B3-Alexa Fluor-546 were ordered from Molecular Instruments, Inc.

### Imaging

Representative specimens from colorimetric in situ hybridization experiments were imaged with a Zeiss Axiocam 503 color connected to a Zeiss Axioscope 5 using bright-field Nomarski optics. Fluorescently labeled samples were scanned on an Olympus FV3000 confocal laser-scanning microscope and Leica SP8 confocal laser-scanning microscope. Colorimetric in situs stained with antibodies were scanned in a Leica SP5 confocal laser-scanning microscope using reflection microscopy protocol as described by Jékely and Arendt, 2007. Images were analyzed with Imaris 9.8.0 and Photoshop CS6 (Adobe), and figure plates were assembled with Illustrator CC. Brightness/contrast and color balance adjustments were applied to the whole image, not parts.

### Electrophysiological recordings and data analysis

For expression in *X. laevis* oocytes and electrophysiological experiments, coding sequences were mutated synonymously to remove internal restriction sites if necessary and subcloned into SalI and XbaI sites of a modified pSP64poly(A) vector (ProMega), containing 5’ SP6 sequence, 5′- and 3′-UTR sequences of the *X. laevis* β-globin gene, and a C-terminal Myc tag, with an EcoRI restriction site after the poly(A) tail (Supplementary file 3). *L. anatina* ASIC (g20471.t1 from *L. anatina* Ver 2.0, OIST Marine Genomics Unit), synonymously mutated to remove internal restriction sites and including a C-terminal myc tag before the stop codon, was commercially synthesized and subcloned (Genscript) into HindIII and BamHI sites of pSP64poly(A) (Supplementary file 3). Plasmids were linearized with EcoRI, and cRNA was synthesized in vitro with SP6 Polymerase (mMessage mMachine kit, Ambion). Stage V/VI *X. laevis* oocytes, purchased from Ecocyte Bioscience (Dortmund, Germany), were injected with 3–90 ng cRNA. After injection, oocytes were incubated for 1–3 days at 19°C in 50% Leibowitz medium (Merck) supplemented with 0.25 mg/ml gentamicin, 1 mM L-glutamine, and 15 mM HEPES (pH 7.6). Whole-cell currents were recorded from oocytes by two-electrode voltage clamp using an OC-725C amplifier (Warner Instruments) and an LIH 8+8 digitizer with Patchmaster software (HEKA), acquired at 1 kHz and filtered at 200 Hz. Currents were also analyzed in pClamp v10.7 software (Molecular Devices) and additionally filtered at 10 Hz (*I. pulchra* ASIC, which has faster kinetics) or 1 Hz (for all other ASICs) for display in figures. Oocytes were clamped at –60 mV, unless otherwise indicated, and continuously perfused with a bath solution containing (in mM): 96 NaCl, 2 KCl, 1.8 CaCl_2_, 1 MgCl_2_, and 5 HEPES (for pH >6.0) or 5 MES (for pH ≤6.0). pH was adjusted with NaOH, HCl, or KOH, as appropriate. Low-Ca^2+^ bath solution contained (in mM): 96 NaCl, 2 KCl, 0.1 CaCl_2_, and 5 HEPES. In most experiments, activating/desensitizing pH was applied to oocytes in-between resting periods (at pH 7.5 for most ASICS, at pH 9.0, 8.6, and 8.0 for *O. fusiformis, L. anatina, S. californicum* ASICs, respectively, unless otherwise indicated) of at least 30 s. After retrieving current amplitude from pClamp, all data analyses were performed in Prism v9 (GraphPad Software). In concentration-response graphs, currents are normalized to maximum proton-gated current. For ion selectivity experiments, IV relationships were measured in regular bath solution and that in which extracellular NaCl was replaced with KCl. IV relationships were obtained by activating the channels at different membrane potentials from –80 to 60 mV, with 20 mV increments, unless otherwise indicated. Reversal potentials (V_rev,Na+_ and V_rev,K+_) were taken from the intersection of the IV curve with the voltage axis. These values were used to calculate relative permeability P_Na+_/P_K+_ with the Goldman-Hodgkin-Katz equation, P_Na+_/P_K+_=exp(F(V_rev,Na+_ – V_rev,K+_)/RT), where F=Faraday constant, R=gas constant, and T=293 K. Standard chemicals were purchased from Merck. Specialist chemicals (Figure 2—figure supplement 1) were purchased from Santa Cruz Biotechnology (item sc-222407, sodium ursodeoxycholic acid, ≥98% purity) or synthesized by Genscript ((pyroE)WLGGRFamide, ≥97% purity—‘Hydra RFamide I’ from Assmann et al., 2014).

## Data Availability

The amino acid sequence alignment used to generate the phylogenetic tree is available in Supplementary file 1. All DNA sequences used for in situ hybridization and hybridization chain reaction are available in Supplementary file 2 or Supplementary file 3. All DNA sequences used for electrophysiological experiments are available in Supplementary file 3. Figure 3—source data 1, Figure 4—source data 1, and Figure 5—source data 1 contain the numerical data used to generate the respective figures. Table 1—source data 1 contains much of the numerical data used to generate Table 1 (the rest is already included in the other source data files).

## Additional files

Supplementary file 1. Amino acid sequence alignment used for acid-sensing ion channel (ASIC) phylogenies.

Supplementary file 2. DNA sequences of neuronal and endodermal genes utilized in in situ hybridization or *hybridization chain reaction* gene expression studies. Aromatic amino acid hydroxylase gene tree.

Supplementary file 3. Modified pSP64 vector and inserts used for *Xenopus laevis* expression and electrophysiology.

MDAR checklist

## References

[bib1] Achatz JG, Martinez P (2012). The nervous system of isodiametra pulchra (acoela) with a discussion on the neuroanatomy of the xenacoelomorpha and its evolutionary implications. Frontiers in Zoology.

[bib2] Aguilar-Camacho JM, Foreman K, Aharoni R, Gründer S, Moran Y (2022). Functional Analysis in a Model Sea Anemone Reveals Phylogenetic Complexity and a Role in Cnidocyte Discharge of DEG/ENaC Ion Channels. bioRxiv.

[bib3] Albertin CB, Simakov O, Mitros T, Wang ZY, Pungor JR, Edsinger-Gonzales E, Brenner S, Ragsdale CW, Rokhsar DS (2015). The octopus genome and the evolution of cephalopod neural and morphological novelties. Nature.

[bib4] Andrikou C, Passamaneck YJ, Lowe CJ, Martindale MQ, Hejnol A (2019a). Molecular patterning during the development of phoronopsis harmeri reveals similarities to rhynchonelliform brachiopods. EvoDevo.

[bib5] Andrikou C, Thiel D, Ruiz-Santiesteban JA, Hejnol A (2019b). Active mode of excretion across digestive tissues predates the origin of excretory organs. PLOS Biology.

[bib6] Anisimova M, Gil M, Dufayard JF, Dessimoz C, Gascuel O (2011). Survey of branch support methods demonstrates accuracy, power, and robustness of fast likelihood-based approximation schemes. Systematic Biology.

[bib7] Arendt D (2021). Elementary nervous systems. Philosophical Transactions of the Royal Society of London. Series B, Biological Sciences.

[bib8] Assmann M, Kuhn A, Dürrnagel S, Holstein TW, Gründer S (2014). The comprehensive analysis of DEG/enac subunits in hydra reveals a large variety of peptide-gated channels, potentially involved in neuromuscular transmission. BMC Biology.

[bib9] Bump P, Khariton M, Stubbert C, Moyen NE, Yan J, Wang B, Lowe CJ (2022). Comparisons of cell proliferation and cell death from tornaria larva to juvenile worm in the hemichordate schizocardium californicum. EvoDevo.

[bib10] Cameron CB, Perez M (2012). Spengelidae (hemichordata: enteropneusta) from the eastern pacific including a new species, schizocardium californicum, from california. Zootaxa.

[bib11] Cannon JT, Vellutini BC, Smith J, Ronquist F, Jondelius U, Hejnol A (2016). Xenacoelomorpha is the sister group to nephrozoa. Nature.

[bib12] Carrillo-Baltodano AM, Seudre O, Guynes K, Martín-Durán JM (2021). Early embryogenesis and organogenesis in the annelid owenia fusiformis. EvoDevo.

[bib13] Chen GQ, Cui C, Mayer ML, Gouaux E (1999). Functional characterization of a potassium-selective prokaryotic glutamate receptor. Nature.

[bib14] Chiu J, DeSalle R, Lam HM, Meisel L, Coruzzi G (1999). Molecular evolution of glutamate receptors: a primitive signaling mechanism that existed before plants and animals diverged. Molecular Biology and Evolution.

[bib15] Choi HMT, Schwarzkopf M, Fornace ME, Acharya A, Artavanis G, Stegmaier J, Cunha A, Pierce NA (2018). Third-generation in situ hybridization chain reaction: multiplexed, quantitative, sensitive, versatile, robust. Development.

[bib16] Coric T, Passamaneck YJ, Zhang P, Di Gregorio A, Canessa CM (2008). Simple chordates exhibit a proton-independent function of acid-sensing ion channels. FASEB Journal.

[bib17] Correa D (1960). Two new marine turbellaria from Florida. Bulletin of Marine Science.

[bib18] Delle Chiaje S (1841). Descrizione e Notomia Degli Animali Invertebrati Della Sicilia Citeriore: Osservati Vivi Negli Anni 1822-1830.

[bib19] De Mulder K, Kuales G, Pfister D, Willems M, Egger B, Salvenmoser W, Thaler M, Gorny AK, Hrouda M, Borgonie G, Ladurner P (2009). Characterization of the stem cell system of the acoel isodiametra pulchra. BMC Developmental Biology.

[bib20] Deval E, Lingueglia E (2015). Acid-sensing ion channels and nociception in the peripheral and central nervous systems. Neuropharmacology.

[bib21] Diochot S, Baron A, Salinas M, Douguet D, Scarzello S, Dabert-Gay AS, Debayle D, Friend V, Alloui A, Lazdunski M, Lingueglia E (2012). Black mamba venom peptides target acid-sensing ion channels to abolish pain. Nature.

[bib22] Dong X, Ko KH, Chow J, Tuo B, Barrett KE, Dong H (2011). Expression of acid-sensing ion channels in intestinal epithelial cells and their role in the regulation of duodenal mucosal bicarbonate secretion. Acta Physiologica.

[bib23] Du J, Reznikov LR, Price MP, Zha X, Lu Y, Moninger TO, Wemmie JA, Welsh MJ (2014). Protons are a neurotransmitter that regulates synaptic plasticity in the lateral amygdala. PNAS.

[bib24] Elkhatib W, Smith CL, Senatore A (2019). A na+ leak channel cloned from trichoplax adhaerens extends extracellular ph and ca2+ sensing for the DEG/enac family close to the base of metazoa. Journal of Biological Chemistry.

[bib25] Elkhatib W, Yanez-Guerra L, Mayorova TD, Currie MA, Perera M, Singh A, Gauberg J, Senatore A (2022). Function and Phylogeny Support the Independent Evolution of Acid-Sensing Ion Channels in the Placozoa. bioRxiv.

[bib26] Foster VS, Rash LD, King GF, Rank MM (2021). Acid-sensing ion channels: expression and function in resident and infiltrating immune cells in the central nervous system. Frontiers in Cellular Neuroscience.

[bib27] Freeman G (1993). Metamorphosis in the brachiopod terebratalia: evidence for a role of calcium channel function and the dissociation of shell formation from settlement. The Biological Bulletin.

[bib28] Fu H, Jiao Z, Li Y, Tian J, Ren L, Zhang F, Li Q, Liu S (2021). Transient receptor potential (trp) channels in the pacific oyster (*crassostrea gigas*): genome-wide identification and expression profiling after heat stress between *c. gigas* and *c. angulata*. International Journal of Molecular Sciences.

[bib29] Gąsiorowski L, Andrikou C, Janssen R, Bump P, Budd GE, Lowe CJ, Hejnol A (2021). Molecular evidence for a single origin of ultrafiltration-based excretory organs. Current Biology.

[bib30] Golubovic A, Kuhn A, Williamson M, Kalbacher H, Holstein TW, Grimmelikhuijzen CJP, Gründer S (2007). A peptide-gated ion channel from the freshwater polyp hydra. The Journal of Biological Chemistry.

[bib31] Gonzalez P, Uhlinger KR, Lowe CJ (2017). The adult body plan of indirect developing hemichordates develops by adding a hox-patterned trunk to an anterior larval territory. Current Biology.

[bib32] Gonzalez P, Jiang JZ, Lowe CJ (2018). The development and metamorphosis of the indirect developing acorn worm *schizocardium californicum* (enteropneusta: spengelidae). Frontiers in Zoology.

[bib33] González-Inchauspe C, Urbano FJ, Di Guilmi MN, Uchitel OD (2017). Acid-sensing ion channels activated by evoked released protons modulate synaptic transmission at the mouse calyx of held synapse. The Journal of Neuroscience.

[bib34] Gründer S, Pusch M (2015). Biophysical properties of acid-sensing ion channels (asics). Neuropharmacology.

[bib35] Gruol DL, Barker JL, Huang LY, MacDonald JF, Smith TG (1980). Hydrogen ions have multiple effects on the excitability of cultured mammalian neurons. Brain Research.

[bib36] Gu Q, Lee LY (2006). Characterization of acid signaling in rat vagal pulmonary sensory neurons. American Journal of Physiology. Lung Cellular and Molecular Physiology.

[bib37] Guindon S, Dufayard JF, Lefort V, Anisimova M, Hordijk W, Gascuel O (2010). New algorithms and methods to estimate maximum-likelihood phylogenies: assessing the performance of phyml 3.0. Systematic Biology.

[bib38] Haszprunar G (2016). Review of data for a morphological look on xenacoelomorpha (bilateria incertae sedis). Organisms Diversity & Evolution.

[bib39] Heger P, Zheng W, Rottmann A, Panfilio KA, Wiehe T (2020). The genetic factors of bilaterian evolution. eLife.

[bib40] Hejnol A, Pang K (2016). Xenacoelomorpha’s significance for understanding bilaterian evolution. Current Opinion in Genetics & Development.

[bib41] Helm C, Vöcking O, Kourtesis I, Hausen H (2016). Owenia fusiformis-a basally branching annelid suitable for studying ancestral features of annelid neural development. BMC Evolutionary Biology.

[bib42] Holzer P (2015). Acid-sensing ion channels in gastrointestinal function. Neuropharmacology.

[bib43] Howard RJ, Edgecombe GD, Shi X, Hou X, Ma X (2020). Ancestral morphology of ecdysozoa constrained by an early cambrian stem group ecdysozoan. BMC Evolutionary Biology.

[bib44] Hulett RE, Potter D, Srivastava M (2020). Neural architecture and regeneration in the acoel *hofstenia miamia*. Proceedings. Biological Sciences.

[bib45] Hulett RE, Kimura JO, Bolaños DM, Luo YJ, Ricci L, Srivastava M (2022). Acoel Single-Cell Atlas Reveals Expression Dynamics and Heterogeneity of a Pluripotent Stem Cell Population. bioRxiv.

[bib46] Ikeuchi M, Kolker SJ, Burnes LA, Walder RY, Sluka KA (2008). Role of ASIC3 in the primary and secondary hyperalgesia produced by joint inflammation in mice. Pain.

[bib47] Jang W, Lee S, Choi SI, Chae HS, Han J, Jo H, Hwang SW, Park CS, Kim C (2019). Impairment of proprioceptive movement and mechanical nociception in *Drosophila melanogaster* larvae lacking ppk30, a *Drosophila* member of the degenerin/epithelial sodium channel family. Genes, Brain, and Behavior.

[bib48] Jasti J, Furukawa H, Gonzales EB, Gouaux E (2007). Structure of acid-sensing ion channel 1 at 1.9 A resolution and low ph. Nature.

[bib49] Jékely G, Arendt D (2007). Cellular resolution expression profiling using confocal detection of NBT/BCIP precipitate by reflection microscopy. BioTechniques.

[bib50] Jones NG, Slater R, Cadiou H, McNaughton P, McMahon SB (2004). Acid-induced pain and its modulation in humans. The Journal of Neuroscience.

[bib51] Kapli P, Natsidis P, Leite DJ, Fursman M, Jeffrie N, Rahman IA, Philippe H, Copley RR, Telford MJ (2021). Lack of support for deuterostomia prompts reinterpretation of the first bilateria. Science Advances.

[bib52] Katoh K, Standley DM (2013). MAFFT multiple sequence alignment software version 7: improvements in performance and usability. Molecular Biology and Evolution.

[bib53] Kaulich E, McCubbin PTN, Schafer WR, Walker DS (2022). Physiological Insight into the Conserved Properties of *Caenorhabditis elegans* Acid-Sensing DEG/ENaCs. bioRxiv.

[bib54] Kellenberger S, Schild L, Ohlstein EH (2015). International union of basic and clinical pharmacology: XCI structure, function, and pharmacology of acid-sensing ion channels and the epithelial na + channel. Pharmacological Reviews.

[bib55] Krishtal OA, Pidoplichko VI (1981). Receptor for protons in the membrane of sensory neurons. Brain Research.

[bib56] Krishtal O (2015). Receptor for protons: first observations on acid sensing ion channels. Neuropharmacology.

[bib57] Kuehn E, Clausen DS, Null RW, Metzger BM, Willis AD, Özpolat BD (2022). Segment number threshold determines juvenile onset of germline cluster expansion in *Platynereis dumerilii*. Journal of Experimental Zoology. Part B, Molecular and Developmental Evolution.

[bib58] Kumar S, Stecher G, Suleski M, Hedges SB (2017). TimeTree: a resource for timelines, timetrees, and divergence times. Molecular Biology and Evolution.

[bib59] Laumer CE, Fernández R, Lemer S, Combosch D, Kocot KM, Riesgo A, Andrade SCS, Sterrer W, Sørensen MV, Giribet G (2019). Revisiting metazoan phylogeny with genomic sampling of all phyla. Proceedings. Biological Sciences.

[bib60] Liebeskind BJ, Hillis DM, Zakon HH (2015). Convergence of ion channel genome content in early animal evolution. PNAS.

[bib61] Liebeskind BJ, Hillis DM, Zakon HH, Hofmann HA (2016). Complex homology and the evolution of nervous systems. Trends in Ecology & Evolution.

[bib62] Luo YJ, Takeuchi T, Koyanagi R, Yamada L, Kanda M, Khalturina M, Fujie M, Yamasaki SI, Endo K, Satoh N (2015). The lingula genome provides insights into brachiopod evolution and the origin of phosphate biomineralization. Nature Communications.

[bib63] Lynagh T, Mikhaleva Y, Colding JM, Glover JC, Pless SA (2018). Acid-sensing ion channels emerged over 600 mya and are conserved throughout the deuterostomes. PNAS.

[bib64] Marinković M, Berger J, Jékely G (2020). Neuronal coordination of motile cilia in locomotion and feeding. Philosophical Transactions of the Royal Society of London. Series B, Biological Sciences.

[bib65] Martin GG (1978). Ciliary gliding in lower invertebrates. Zoomorphologie.

[bib66] Martín-Durán JM, Pang K, Børve A, Lê HS, Furu A, Cannon JT, Jondelius U, Hejnol A (2018). Convergent evolution of bilaterian nerve cords. Nature.

[bib67] Martindale MQ, Pang K, Finnerty JR (2004). Investigating the origins of triploblasty: “mesodermal” gene expression in a diploblastic animal, the sea anemone *Nematostella vectensis* (phylum, cnidaria; class, anthozoa). Development.

[bib68] Matthews BJ, Younger MA, Vosshall LB (2019). The ion channel ppk301 controls freshwater egg-laying in the mosquito aedes aegypti. eLife.

[bib69] Moroz LL, Kocot KM, Citarella MR, Dosung S, Norekian TP, Povolotskaya IS, Grigorenko AP, Dailey C, Berezikov E, Buckley KM, Ptitsyn A, Reshetov D, Mukherjee K, Moroz TP, Bobkova Y, Yu F, Kapitonov VV, Jurka J, Bobkov YV, Swore JJ, Girardo DO, Fodor A, Gusev F, Sanford R, Bruders R, Kittler E, Mills CE, Rast JP, Derelle R, Solovyev VV, Kondrashov FA, Swalla BJ, Sweedler JV, Rogaev EI, Halanych KM, Kohn AB (2014). The ctenophore genome and the evolutionary origins of neural systems. Nature.

[bib70] Nakajima Y, Humphreys T, Kaneko H, Tagawa K (2004). Development and neural organization of the tornaria larva of the hawaiian hemichordate, ptychodera flava. Zoological Science.

[bib71] Ng R, Salem SS, Wu ST, Wu M, Lin HH, Shepherd AK, Joiner WJ, Wang JW, Su CY (2019). Amplification of *Drosophila* olfactory responses by a DEG/enac channel. Neuron.

[bib72] Nielsen C (2015). Larval nervous systems: true larval and precocious adult. The Journal of Experimental Biology.

[bib73] Onoa B, Li H, Gagnon-Bartsch JA, Elias LAB, Edwards RH (2010). Vesicular monoamine and glutamate transporters select distinct synaptic vesicle recycling pathways. The Journal of Neuroscience.

[bib74] Pattison LA, Callejo G, St John Smith E (2019). Evolution of acid nociception: ion channels and receptors for detecting acid. Philosophical Transactions of the Royal Society of London. Series B, Biological Sciences.

[bib75] Paukert M, Sidi S, Russell C, Siba M, Wilson SW, Nicolson T, Gründer S (2004). A family of acid-sensing ion channels from the zebrafish: widespread expression in the central nervous system suggests A conserved role in neuronal communication. The Journal of Biological Chemistry.

[bib76] Paukert M, Chen X, Polleichtner G, Schindelin H, Gründer S (2008). Candidate amino acids involved in H+ gating of acid-sensing ion channel 1a. The Journal of Biological Chemistry.

[bib77] Philippe H, Poustka AJ, Chiodin M, Hoff KJ, Dessimoz C, Tomiczek B, Schiffer PH, Müller S, Domman D, Horn M, Kuhl H, Timmermann B, Satoh N, Hikosaka-Katayama T, Nakano H, Rowe ML, Elphick MR, Thomas-Chollier M, Hankeln T, Mertes F, Wallberg A, Rast JP, Copley RR, Martinez P, Telford MJ (2019). Mitigating anticipated effects of systematic errors supports sister-group relationship between xenacoelomorpha and ambulacraria. Current Biology.

[bib78] Pixell HLM (1912). Memoirs: two new species of the phoronidea from vancouver island. Journal of Cell Science.

[bib79] Price MP, McIlwrath SL, Xie J, Cheng C, Qiao J, Tarr DE, Sluka KA, Brennan TJ, Lewin GR, Welsh MJ (2001). The DRASIC cation channel contributes to the detection of cutaneous touch and acid stimuli in mice. Neuron.

[bib80] Raikova OI, Meyer-Wachsmuth I, Jondelius U (2016). The plastic nervous system of nemertodermatida. Organisms Diversity & Evolution.

[bib81] Rattenbury JC (1954). The embryology of phoronopsis viridis. Journal of Morphology.

[bib82] Ringers C, Olstad EW, Jurisch-Yaksi N (2020). The role of motile cilia in the development and physiology of the nervous system. Philosophical Transactions of the Royal Society of London. Series B, Biological Sciences.

[bib83] Rook ML, Musgaard M, MacLean DM (2021). Coupling structure with function in acid-sensing ion channels: challenges in pursuit of proton sensors. The Journal of Physiology.

[bib84] Roy S, Boiteux C, Alijevic O, Liang C, Bernèche S, Kellenberger S (2013). Molecular determinants of desensitization in an enac/degenerin channel. FASEB Journal.

[bib85] Santagata S, Resh C, Hejnol A, Martindale MQ, Passamaneck YJ (2012). Development of the larval anterior neurogenic domains of terebratalia transversa (brachiopoda) provides insights into the diversification of larval apical organs and the spiralian nervous system. EvoDevo.

[bib86] Schmidt-Rhaesa A, Bartolomaeus T, Lemburg C, Ehlers U, Garey JR (1998). The position of the arthropoda in the phylogenetic system. Journal of Morphology.

[bib87] Shah AS, Ben-Shahar Y, Moninger TO, Kline JN, Welsh MJ (2009). Motile cilia of human airway epithelia are chemosensory. Science.

[bib88] Shannon TR, Achatz JG (2007). Convolutriloba macropyga sp. nov., an uncommonly fecund acoel (acoelomorpha) discovered in tropical aquaria. Zootaxa.

[bib89] Sikes JM, Bely AE (2008). Radical modification of the A-P axis and the evolution of asexual reproduction in convolutriloba acoels. Evolution & Development.

[bib90] Siltberg-Liberles J, Steen IH, Svebak RM, Martinez A (2008). The phylogeny of the aromatic amino acid hydroxylases revisited by characterizing phenylalanine hydroxylase from *Dictyostelium* discoideum. Gene.

[bib91] Simakov O, Marletaz F, Cho SJ, Edsinger-Gonzales E, Havlak P, Hellsten U, Kuo DH, Larsson T, Lv J, Arendt D, Savage R, Osoegawa K, de Jong P, Grimwood J, Chapman JA, Shapiro H, Aerts A, Otillar RP, Terry AY, Boore JL, Grigoriev IV, Lindberg DR, Seaver EC, Weisblat DA, Putnam NH, Rokhsar DS (2013). Insights into bilaterian evolution from three spiralian genomes. Nature.

[bib92] Slota LA, Miranda E, Peskin B, McClay DR (2020). Developmental origin of peripheral ciliary band neurons in the sea urchin embryo. Developmental Biology.

[bib93] Smart TG, Paoletti P (2012). Synaptic neurotransmitter-gated receptors. Cold Spring Harbor Perspectives in Biology.

[bib94] Smith J, Bush L, Smith J (1991). Transactions of the American Microscopical Society.

[bib95] Sowerby GB (1846). Descriptions of thirteen new species of brachiopods. Proceedings of the Zoological Society of London.

[bib96] Srivastava M, Mazza-Curll KL, van Wolfswinkel JC, Reddien PW (2014). Whole-body acoel regeneration is controlled by wnt and bmp-admp signaling. Current Biology.

[bib97] Storozhuk M, Kondratskaya E, Nikolaenko L, Krishtal O (2016). A modulatory role of asics on gabaergic synapses in rat hippocampal cell cultures. Molecular Brain.

[bib98] Su X, Li Q, Shrestha K, Cormet-Boyaka E, Chen L, Smith PR, Sorscher EJ, Benos DJ, Matalon S, Ji HL (2006). Interregulation of proton-gated na (+) channel 3 and cystic fibrosis transmembrane conductance regulator. The Journal of Biological Chemistry.

[bib99] Tasneem A, Iyer LM, Jakobsson E, Aravind L (2005). Identification of the prokaryotic ligand-gated ion channels and their implications for the mechanisms and origins of animal cys-loop ion channels. Genome Biology.

[bib100] Tavernarakis N, Shreffler W, Wang S, Driscoll M (1997). Unc-8, a DEG/enac family member, encodes a subunit of a candidate mechanically gated channel that modulates *C. elegans* locomotion. Neuron.

[bib101] Temereva EN, Tsitrin EB (2014a). Development and organization of the larval nervous system in phoronopsis harmeri: new insights into phoronid phylogeny. Frontiers in Zoology.

[bib102] Temereva EN, Tsitrin EB (2014b). Organization and metamorphic remodeling of the nervous system in juveniles of phoronopsis harmeri (phoronida): insights into evolution of the bilaterian nervous system. Frontiers in Zoology.

[bib103] Ustaoglu A, Sawada A, Lee C, Lei WY, Chen CL, Hackett R, Sifrim D, Peiris M, Woodland P (2021). Heartburn sensation in nonerosive reflux disease: pattern of superficial sensory nerves expressing TRPV1 and epithelial cells expressing ASIC3 receptors. American Journal of Physiology. Gastrointestinal and Liver Physiology.

[bib104] Valentine JW, Collins AG (2000). The significance of moulting in ecdysozoan evolution. Evolution & Development.

[bib105] Vullo S, Bonifacio G, Roy S, Johner N, Bernèche S, Kellenberger S (2017). Conformational dynamics and role of the acidic pocket in ASIC ph-dependent gating. PNAS.

[bib106] Wiemuth D, Gründer S (2010). A single amino acid tunes ca2+ inhibition of brain liver intestine na+ channel (blinac). The Journal of Biological Chemistry.

[bib107] Wiemuth D, Assmann M, Gründer S (2014). The bile acid-sensitive ion channel (basic), the ignored cousin of asics and enac. Channels.

[bib108] Wilson DP (1932). IV on the mitraria larva of owenia fusiformis delle chiaje. Philos Trans R Soc Lond B Biol Sci.

[bib109] Xun X, Cheng J, Wang J, Li Y, Li X, Li M, Lou J, Kong Y, Bao Z, Hu X (2020). Solute carriers in scallop genome: gene expansion and expression regulation after exposure to toxic dinoflagellate. Chemosphere.

[bib110] Zhang G, Fang X, Guo X, Li L, Luo R, Xu F, Yang P, Zhang L, Wang X, Qi H, Xiong Z, Que H, Xie Y, Holland PWH, Paps J, Zhu Y, Wu F, Chen Y, Wang J, Peng C, Meng J, Yang L, Liu J, Wen B, Zhang N, Huang Z, Zhu Q, Feng Y, Mount A, Hedgecock D, Xu Z, Liu Y, Domazet-Lošo T, Du Y, Sun X, Zhang S, Liu B, Cheng P, Jiang X, Li J, Fan D, Wang W, Fu W, Wang T, Wang B, Zhang J, Peng Z, Li Y, Li N, Wang J, Chen M, He Y, Tan F, Song X, Zheng Q, Huang R, Yang H, Du X, Chen L, Yang M, Gaffney PM, Wang S, Luo L, She Z, Ming Y, Huang W, Zhang S, Huang B, Zhang Y, Qu T, Ni P, Miao G, Wang J, Wang Q, Steinberg CEW, Wang H, Li N, Qian L, Zhang G, Li Y, Yang H, Liu X, Wang J, Yin Y, Wang J (2012). The oyster genome reveals stress adaptation and complexity of shell formation. Nature.

