# Supplementary material for: Peripheral and central employment of acid-sensing ion channels during early bilaterian evolution
Source: eLife. 2023 Feb 23;12:e81613. doi: 10.7554/eLife.81613 (PMC9949801; doi:10.7554/eLife.81613)
Supplement: Supplementary file 2. — Aromatic amino acid hydroxylase gene tree. [file elife-81613-supp2.docx]

**Supplementary File 2**

- DNA sequences of neuronal and endodermal genes utilized in *in situ hybridization* or *hybridization chain reaction* gene expression studies
- Aromatic amino acid hydroxylase gene tree

>IpulchraChAT_KY709762.1

TATTACTTTTAAATAGATTAATGAGCAAAGAGAACATCGGAGACCAAATCTGCGACTATGACGATCATCAGAACTTTAGCACGACCAATCACCTGTACCAGCTACGTCTGGGGAAGCAGCCAGTGCCAGATCTGGCGTCTTCAATCGAGGACTTCTTGCTTCACAGCGGGGCGGTGCTCAGCCAATCGGATCAAAGCACGCTGACCCAACTGGCACATGAATTCGTCGCCACTAGAGGGCCTTCTCTCGTGGAGAAACTGGCCAACTCGACCCACGGACCAACAGCCAACTGGAGTTACGGGCCGTGGGTGCGGGACATGTACCTGCGGTGCCGGAAGCCGCTGCCGGTGTACTCGAACCCGGCCATGCTGGTGGAGAGGCGGGCGTTCCTCACCGAGAACCACTGGCTCCGGTTCGCCGCTCGGGTGGTGTGCGAGCTGCTCCGCTACCGGCACATGCTCATCACCGGGAAGACGGGTCTTGAGTACGTGCGGAGTCTGGAGTTCGCGGGCGGGAAGCAGCCACTGTGCATGGAGCAGTACCGGTCCCTCTTCACCTCGTGCAGACGGCCGGGCGAGGAGCTCGACTCCCAAGTCAGGTTCCCCGACAACTTCTCGCTCGCCCACCGGCTCCACGTCATCGTCGCCTTCAGGGGCAAGTTGTACACGGTGAGGCTGGAAGCAGCGGATGAGGGAGTGCCGGACTTGGAGCCACTCGCGGCTTCCCTGAGAGAGATTGTCCACGCCGGGCGCAATGCCGCGATGGGTGTCGGCGACGAGCTGGGCCTGCTCACCGGCGCTCCCAGACATGAGTGGGCTCACGTCTACAACTCCCTCTGCAAGGTGGAGAAGAATGCGTGCAATATCCGGGAGCTGGAGTCGGCAGTGTTGGTCCTCTGTCTGGACGAGGAGGCGGACGGAAGTCCTGATGACTCACGGGACCTGACCCGACTGCTGACGGGCGGGGAGCACAGTCAGTCCAACCGCTGGTTCGACAAGGCCATCCAGGTGATCGTGGGGTCAGACGGGCTGCTCGGGGTCAACGTGGAGCACTCGACCGCCGAGGGGGTCGTCCTCGTGAGGATCATGCAGCAGGTCCTCGCTAATCTGCAGGAAGAGACTTGCACGGAGACGAACGGTGACTGTAACGGTGTTGACGGAAACAAAGTAATCCCTCTAGACTGGGTTATCAACGCCAAACTAGCGTCCAAGATTAAAGAAACCGCTGCAAATCAGAAACGGCTACTCAACGATCTCAGTATCAGAAAAGTAGTTCTAGATGACATTGGAAAGAGGTGGATCAAGAGCAGAGGCGTCAACCCCGATGTGTTCGTTCAAATCCTGCTGCAGCTCACATATCACGCATGTCACGGGCAGCTGTGTCACACCTACGAGAGCGCCTCCTTGCGGCGGTTTGAGCGCGGCAGGGTTGACAACATCAGGTCGAATAACCCGGCTGTTCTTCGACTGGCCCAGGCAATCAGCCAACACAGAGGCGAGGAGGAGCTCCAGGCTCTCTTCCACGAGGCCTGCAGATTCCAGCAGCGCACCATCGACAAGGTTATCTCGGGGCACGGGTGCGACCTGCTGCTGCAGAGCCTGAGAGAAGAGGTCACGGCCGAAGGAAGGAGAGATGCGTTCCTCCACAGCGATCTGTTCACTCAGTGCTGGTCCTTCGACCTTGGTACCAGTCAGGTGACGATGCCGGAGGGTCCGTTCATGTTCTACGGACCGATGGAGGCGTCGGGGTACGGGGCGTGCTACAACCTGCTGGAGGACCGCGCCGTCCTCTGCCTCTCCGCCCTCCAGTCCTCCCCGAGGAGCAGCCTCAGTCTCTTCTGCACCAACCTCACCAGAGTCAAAGACACCCTTCTCTCTATCACATGACCGTACAGCAATTACCGTCCTCCAATATTGCATTATTGTACCGAATATTGTAATCAATTTATATGTTCGCGCAATCTTGTCAAGCGCAGTCTACTTTGATCTATTTTTAGCAATTGATGTTCAATCTACCGTGCGGAAATTAAG

>IpulchraVAchT_KY709762.1

GTCCGGACTCATCTGGCCGTCTTTCCCCGCTGACAGGAACTCAATGTCGTCATACGAGGGCATACCGGGCGATAGCGGCGGCGGCGGTGGGGCGGGTTCCGCCAATCAGGTGCCGCGGTCGGAGTCGGAGCCGCTGATGGAGGCGTACCGGAAGTTCTCGGCCAAGCTGGAGGAGCCGAAGCTGCAGCGGTTCCTGGTGCTGGTGATCGTGAGCGCGGCGCTGCTGCTGGACAATATGCTGTACATGGTCATCGTGCCCATCATCCCCGACTTCCTCCGCAAGCACGACGCGTGGCACGTGCAGACCGAGTACAGTGTCAAGACCGAGCTGGTGGACGGCCAGTGGCGCAACACCACCTACGCCACCGACGTCAACTACGAGGGCGAGGACACCAGCCTCGGCTTCCTGTTCGCCAGCAAGGCGTTCATCCAGCTGATCGCCAACCCCGTGTCCGGCACCCTGATCGACCGGGTGGGCTACGAGTTCCCGATGGTGTTCGGCCTGTTCGTCATGTTCGTCTCCACCACCATCTTCGCGTTCGGGGAGACTTACACCTTCCTCTTCCTCGCCCGCTGCATGCAGGGCATTGGGTCCGCGTTCGCCGACACCGCCGGGCTCGCGATGATCGCGGACCGGTTCACGGAGGAGCGGGAGCGGAACCGGGCGCTGGGCATCGCCCTGGCCTTCATCAGCTTCGGCTTCCTCTTCGCCCCGCCGTTCGGGGGCGTGCTCTACATGCTGGGCGGGAAGGAGGTGCCCTTCCTCTGCCTCGCCTGCATCGCCCTCCTCGACGCCGGCATGCTCCTCCTCGTGTCCTCCACCGAGACCCACAAGCGCACCCAGTCATCCAAGGGGCACTCGGCGCAGGCGCACGCGCCGATATACAAGCTGCTGCTGGACAAGCACGTCATCGTCATCTCGTGCGCGCTCATCATGGCCAACGTGAGCCTCGCGTTCCTGGAGCCGACCATCGCCACCTGGATGGAGGACTACATGGACGCCGACCAGTGGCAGCAGGGCATCATCTGGCTCCCCGGCTTCTTCCCACACATCCTCGGAGTGTACATTTGCGTGAAGCTGTCCAACAAGTATGCGCGGACACAGTACATATACGCGGGAGTGGGTCTGATCATCATCGGAGTGTCCACCTGCTTCATACCCGCCTGCTCCACCTTCACCCTCCTCATCCTGCCCATCTGCGGCATGTGCTTCGGCATCGCCCTCGTCGACACCGCCCTCATTCCCGCACTCTCATATCTCGTCGACATCCGGTACACGTCAGTGTACGGGAGCGTGTACGCGATC

>IpulchraVMAT_KY709764.1

CTGGCTTTTGTCCCCGCTTTCTCCTCCATTCCAGCTGCACTCCCGACAAAGGCTGGTACCACACAGTGCGCAACGAGTGGCCCGACTCCACTCGTGCATGCCATCATGTATTACCAGTCAACCCACGGTCCCGTCCCCGATACCGACCAACACTTGGATGACTCATCGTTCGTCCACGTCGACTGCGCCCCCTTCCCGCCCCCTCCGGAGTACAGCGGGGGCATCTCGTTCGGGGGGATGCCCAGCCTCGACTGCAGCTGCTGGTCTCCGCAGTTCTGGAGCGAAGTGCGCAGCTCCAAGCGACTGCTCATATTCATCGTTTTCATAGCTCTTCTGTTAGATAACATGTTGCTCACCAGTGTAGTACCGATCATACCGGACTACCTGTACAAGTTGGAGCATCCGGATGGGCCGATCCAGGCACCACAGCCTCCACTTGCCCAACAGCCATTCTACGTGAACGAGAGTGTGGAATACACCGACTACTTCTCCCCGGGCGCCGACCAGTCCAAGCCTGGCCCCCCGTCTGCCCATTACTCCAGCTCGTCCGCCTACGCAGATGAACTGAATAAAGAGAACATCCGGGTCGGGCTGCTGTTCGCTTCCAAGGCCCTAGTCCAGTTGGTCACCAATCCATTCGTCGGCATCCTTACCAACAAGGTTGGGTTCAGTCTGCCGATGTTCGTGGGCTTCGTCATCCTCATCCTGTCGACCGTCATGTTCGCGTTCGGGGAGTCGTACTCGGTGCTGATGGTGGCGCGGAGTCTGCAGGGCATCGGGTCGTCCTGCTCCTCGGTCGCGGGCATGGGCATGCTGGCGGAGCGATTCCCCGACGACGAGGAGCGCGGCAACGCCATGGGGGTCGCCCTCGGGGGCCTCGCCATGGGCGTCCTCATCGGGCCGCCGTTTGGGGGCTTCATGTACCAGTTCGTGGGGAAGCCGGCGCCGTTCCTGGTGCTGGCGGCGGTGGCCCTCCTGGACGGCACCCTCCAGCTCATCGTCCTCCGCCCCGGGATGAAGACCGAGCACCAGCCCCGCGGAACCCCCCTCACCACCCTCCTCAAGGACCCTTACATCCTCATCGCCGCCGGCTCGATAACATTCGCGAACATGGCGATCGCCCTGCTGGAGCCAACACTCCCCATATGGATGATGGAGAACATGAACTCCGAAAAGTGGCAGCTCGGGGCTGCATTCTTGCCAGCCAGTGTTTCCTATCTCATCAGCACCAACATAAACGGGCCTCTGACAACAAAGGTCGGAAGGTGGATCTCGTCTCTCATCGGGATGGTCATCGTCGGAATCTGCATGATGCTGTACCCTCTTGCCTCCACCATGAACGGGCTGATAATCCCGGGCTTCGGCCTGGGCTTCGCCATCGGAATGGTGGACGCGTCGATGATGCCAATGATGGGGTACCTGGTTGACCTCAGGCACGTGTCGGTGTACGGGAGCGTGTACGCGATCGCGGACGTCGCGTTCTGCGTGGGTTACGCCGTCGGGCCCGCCATGAGCGGCTACATCGTCGAGGGCATCGGCTTCCCATGGCTAGTCCGCATCATCGCCATCGTCAACATACTATTCGCACCGCTGCTCTATTATCTGAAGGAACCGCCCACTGGCAAAGAAGAGACCCAGAACCTCATAGAGACCAACGAAGATCAGTATGCGGGAAAGTACCGTCGAGCTGACGGCTACGCAACCCTCGAGGAAGAATAACTGAAGACCCTTTCAATTATATCATAGTTGTCTATCTTGTAATTAACGAATGCATAAAATCCAGGAG

>IpulchraSyn_Ipul.rna.tri.10721.1

GGCATGGATATGTCAGGAACCTCTGACCCTTACGTCAAGGTCTACCTCCTGCCCGACAAGAAGAAGAAACAGGAGACCAAAGTACACCGCAAGACACTCAACCCCATCTTCAACGAAACCTTCAAGTTCAAGATCCCGTTCGCGGAGATCGGGGGCCAGACTCTGGTCCTGTCGGTGTACGACTTTGACCGGTTCTCCAAACACGACATGATCGGAATGCTCAAGATCAACCTCAACTCCATCGACCTCGGCAACACGTACGAGGCCAACAAGGAGCTCAACCCGCCCGACGACGACAAGGAGTATCTGGGTGACCTGTGTTTCTCGCTTCGTTACGTGCCCAAGGCGGGCAAGCTGACCGTCAACGTGCTGGAAGCCAAGAACCTCAAGAAGATGGATGTCGGCGGCCTCTCTGACCCCTTCGTCAAGATCGAGCTCATGCAGGCTGGGAAGAGACTGAAGAAAAAGAAGACGACGATTAAGAAGCGGACTCTCAACCCGTACTTCAACGAAAGCTTCCTATTCGAGGTTCCCTTCGAGCAAATCAGCAAGACAGAACTGAGGATAACAGTGTACGATTACGACAAACTGGGCAGCAACGACGCCATCGGGCTCATCCACGTCGGCTACACGGCCAGCGGGGCCGGATTACGTCACTGGACCGACATGATCAACGCGCCTCGTCGACCAATCGCGCAGTGGCACACGCTTCAGGAG

>IpulchraTH_KY709765.1

AGGTTTGGTACCCGAAGAGCGCCGAGGACCTCAACCGCTGCCGCAACATCCTCAGCTCGGTCGAGCCCGACCTGGACAAGGATCACCCCGGGTTCAGTGACGCGAAATATCGAGCTAGACGATCCGAAATTGCGACTATTGCTCTCAGCTTCAGACATGGCGGGGAGATTCCCCGAGTCGAGTACACAGAGGTGGAGGTGGGGACGTGGCGGGAAGCGTACGAGAACCTCACCGAGCTGCATGAGTCACTGGCGTGCGAGGAGTACAAGACGGCGTTCAAGCGACTTGCACACGACCGGGTCATCAAGCCGGACGAGATTCCGCAGCTTCAGGATTTGTCCGATTATCTGACCGAGAAGACGAACTTTCGGCTGTGTCCAGTGGCGGGGCTGGTGGCTGCCAGAGACTTTCTGGCATGTCTGGCGTTCCGGGTGTTCCCCTGCACCCAGTACATGCGTCACCACGCCGCCTCCATGCACTCCCCTGAACCGGACCTGATCCACGAGGTGCTGGGTCACGTGGTGATGTTCACCAACCCGCTGGTCGCGGACTTCTCCCAGAAGATCAGACTGGCGTCCCTGGCCTTGGTCCCAACTGATATCCAGGGTCTTGAGTGGTGTCTCTCCAGGGACCTGATCCACGAGGTGCTGGGTCACGTGGTGATGTTCACCAACCCGCTGGTCGCGGATTTCTCCCAGAAGATCGGGCTGGCGTCCCTCGGCGCCTCGGACGACTTCATTCAGAAGCTCGCTACTCTATATTGGTTCACGGTGGAATTTGGACTGATCGAAGAATCGAACGGGTTAAAGGCTTTTGGGGCCGGTTTGCTCTCTTCCTACGGAGAACTCGTGTTCGCGGTTTCTGACGAGCCCGAGCATCGTGAGTTCTTCCCAGAAGAGACAGCGGTTCAAGCTTATGACGATTACAACTACCAGACAACTTACTTCGTCGTTAAAAACTTCAATCAGATGACTCAAAAATTCACGAAATACATCGAAGACTACAAGCAATGAATGTTCAAACATAGTCAAAAACCGCATGAGCCAGGCATGGGGCAAGACCATGTATTTCGAAAAACACGCATTCCAATAAAATATTCAATTCGATTGTCC

>IpulchraTpH_KY709766.1

ATTCCCCTTTAAATCCTCGCTCCGGACATTCCCTGTATTTAATAATTTCAAATAGTAATTATTCAGTCCTCCGCTAACCTTCGGCGCTATCGGCCTTTCTTTCTTCTCAATAATTGCAACAATTTGGGAGACTAGCTCAGAATGTATTACGTGAGAAGGAAGTACCTGTTGGATCACTACTCGGCTCCGGAGCACGGCAAGCTTTCGCTCACCAACAACTCCCTGAGACGGAAGCGGGGGGCCTTCCGCTCGGGAGGCACCAAGTCTGGAAGTTTCCAGCTGAGCCTGGAGGCGAACTACGAGGACGCGAATGAGAAGAGCCACTGCGCGGCTGTCATCGTCACCGAAACCTCGGGAGTCGACCAACTCATGGACGTCATGCAGGTCTTCAAGCGCAACGACATAAAGATTAACCACATCGAGTCACGCAAGCCAAAGAACGACGATTCAAAGTCTTCGTCGATTTCTGAGTTCTACGTGGACCTCGAGGGCGAACCAGCAGCTCTTCTGATCGCCATCAACAACCTCAAGAGCAAAGTCAAGGAGTTCACCTTCGGGGAGAAGAGTCCTCTCGTCACTGAGAAGCCTGGAGTGGAGGAGTTGGGAGACGTACCTTGGTTCCCGCGGCGGATCGCCGATCTGGACAAAGTATCCAACCGCGTGTTGATGTACGGAGATGCTCTGGACGCCGATCATCCCGGCTTCAAGGACCCCGTGTACCGGGAACGGAGGAAGATGTACGCAGACATCGCCTACAACTACAAACAGGGGGAGCGGATCCCGCGGATCGAGTACACGGAGCAGGAGAAGGAGACGTGGCGGACGATATACCGGGAGCTGAACCGGCTCTACCCGCTGTACGCGTGTCGCGAGTTCCTGGTCAACCTCCCGCTGCTCCAGCTGTACGCCGGATACAGCGAGCACTGCCTGCCCCAGCTCGAGGACGTCAGCTACTTCCTCAGCGAAAAGACGGGGTTCGTGTTACGGCCGGTGGCGGGCTACCTCTCATCCAGGGACTTCCTGTCGGGGCTGGCGTTCCGTGTCTTCTACTGCACCCAGTACATCCGCCACCACAAGGACCCCTTCTACACACCCGAGCCAGACTGCTGCCACGAACTGCTGGGTCACGTGCCGATGCTGGCGGACCGCAGCTTCGCCGCCTTCTCCCAGGAGATCGGACTGGCCAGCCTCGGCGCCTCCGATGAAGAGGTCGAGCGACTGTCCAAGCTGTATTTCTTCACGGTGGAGTTCGGGCTGTGCAGGCAAGACGGGAAGGTGAAGATCTACGGGGCGGGACTGCTGTCCTCGATCGGGGAGCTGAAGCACGCAATGGAGCACACGGAGAAGCAACGGGACTTCTCCATGAACGCCGTTATGGAAATGGAGTGCAAGATCACCACCTTCCAGGACGGCTACTTCATCAACAACAGCTTCGAGGTCGCCAAGAACGAACTGAGGAACTACGCGGCGTCGATCGACAGACCGTTTCACCTCCGCTACAACCCGAACAGTCGGTGCGTCGAGACCCTCATCACCCAGAGGGAGCTGCTGGCCGCTCTCCAGGAGACCAAGCAGGAGATGACGCAGCTGGTGGACGCAATGGCGCGGCTCAACAACCGGCAGCAGGAGTCCATCATCCCCGAGAGCCGGTGGGACGCTGTGATTGGCTCGCTGTGCCGCCGCCGGGTGTCCAACGACGACGACATCCCCGAGGAGGAGAGCCCGTGCAACGGCGGCGGCATCGCCAACGGCGCCTGAGTCATGGATGACGGCTCATGAGAACGCAGATGATCATATCAATGACTCATATATCGCATACTTGGTCATACAGGCTTCATAAGTTTCAGGCAATCGCAATTTCGTCTCATAAAATGTCAGGTGTCCATCTAACAGAATGATCCAACGCATGATCAGAGGGATTGAAATGCTAGTACAATAATAAGCACAACTCTGTATACATATGCAATTTATCTGTTCA

>HmiamiaGad-1_MT657938.1

GTTTTGAAAGACAATTTTGTGACGAGTGAAAAAAAATTCAATAGACATGTCTGTTGAAAACGTAGTACCAATCAACCGTTTACTGGATGTATCCAGTGTAAAAAGCAGCGCCCTACTGCTGCTGGACGAAGCTCAAGAGTCCAAGGTGCTGAAATCAAAAGATAGAGTTTGCGGTAACACCTCTCTCTCCGCCAAGCAGGTATTATTTGAAAATCAACCGGAAGAAAAGGGAGAAGAAGAAATTAAGCGCCGATCAGTTCTTCGTAAAATGAAGAGCTGTTCCAGCAAATATGATGATGTGTTTGGCACGAGAAATGATTTACGTCGACAATTGAGCCAGGCTAATCAGATGCTTGAAGAAACTAACATTGCAAGAAGACGATTGGACAAGTTAAATATTGATTTCAGCAAGAAATATGCAAAAGAATTGTTACCAAATGAGGCAAATCCGCCAACAAATACTGTTGATTTTCTGAGAGAGGTGATTGAAGAACTCTTTGAGTATGTAAGGCAATCACATGATCGCAATGAACCAGTGCTGGAATTCCACCACCCTATGGAATTGAAAGGGAATTTAGATTTAGAGGTTCCTGAAGGCCCCGAGACTTTGGCGCAGATTTTGAATGACTGTCGTGAGGCTTTGAAATATGCAGTGAAATCAGGTCATCCAAGATTTTTTAATCAGCTTTCGAGTGGGATGGATGTTGTATCTCTTGCTGGCGAATGGTTGACATCAACTGTTAATTCAAACATGTTTACGTATGAAGTATCGCCGGTGTTTGTTCTCATGGAACGAATTGTTTTGAAAAGAATGCGGGAAATCATCGGTTGGGCAGATGGTGAAGGAGATGGCATTTTTGCTCCAGGTGGGACGATCGCTAACTTATATGCTGTTCTCGCTGCACGTCATAAATATTATCCTGAGATCAAAAGTAAAGGAAGCCTGGCTCAAAGTCAGCTGTGCATGTTTACATCGAGGCATGCTCATTATTCGCTGAAGAGTGCTGCGCATATAGTTGGTATTGGCACTGACAATTGCATTACAGTGGAAACCGACGGAATCGGAAAAATGCGTCCTGATGATTTGAAAGCTAAAATTAGCAAAGCTAAGGACGAGGGAAAAGTACCATTCCTGGTGTCATGTACAATGGGAACAACCGTAGTTGGAGCATTTGATCCTATTACGGAGATTGCTGATATTTGCGAAGAACACAACCTGTGGCTTCATGCTGACGCTGCTTGGGGTGGTGGTGCATTAATGAGCAATAAATGGAAACATCTTTTGGATGGCATAAATAGAGCTGATAGCGTTACTTGGAATCCTCACAAAATGATGGGTGCAAGTTTACAATGTTCTGCCATCTTAGTCAAAGAAGATGGAATTCTTGAGAGTTGCAACGCCATGCATGCGTCTTACTTATTTCAAAGAGACAAGCACTATGATATAAGCTATGATACCGGAGACAAAGCAATTCAATGTGGAAGGCATGTCGACATCTTCAAACTATGGCTAATGTGGCGTGCAAAGGGTAGAGTTGGCTTTGAATACCATATGAACCATATGATGGATTTGAAGGCGCATTTGATTAAACTTATCAAAGAAACCGAAGGCTTTGAATTAGTATTTGAAAACCCTGAATACGTCAATGTCTGTTTCTGGTATATTCCACCTTGTCTCCGAAAAAACGCCAAAGGTCCGCAACGTGATTTCGTTGTTAACAAAGTCGCTCCATTTATTAAAGCGCAGATGATGGACAAAGGAGACTTGATGGTGAGCTACCAGCCACTGGATCAATACCCAAATTTCTTTCGGATGGTGATATCAAACCAAGCGGCAACGATGGATGACATTCAATTCGTAATTACTCGTATTGAAGAATTGGGAAAGACGTTCCGTCTCGACTAAATCAATCAAATATTCATGTAGTGGACATTGTGCGCACGTTGTCTTAGTTTATAGAATTACTCAAATAACCTGATTACATGTGCACTGTGTAATTTAGTCTTAAAGTGACTAGTTTGTAATCAGGCGTAGTTTGTATTCCAATTGCAATTAAAAATAAAATAAATAATTGTGCAATGAAAGGAATTTATGATGGTGCTACACGAAAAAGTGAGTCTATTTTGTTAATATGGAAAATGTTTAATGGAAAACATATGTTAAAATAGAAGTAGTTATGAATTTTT

>HmiamiaTpH-1_98024900

ATTGAAACACGTAAGAGTTCAAATGATGCTAATGAAGTAGAAGTGGTATGCCAAGTAGAAGGCATAAAAGAATCTGTAACCGCTGCATCCAGTAAGATAAAGTTATCATCTTTGGTTGATACCAATTCTGAAAAAGCCTTATCTAGTAATGATAATGAGGATTATCAACATGTTTGGTTTCCCAGTAAAATTTCAGAACTGGACAAGGTAGCTGACCGCATTATTGAAGTTGAAAATGTCATGGAAGAGGATTGTGAAGGATGTTCGTATCCTGAATACATGAAAAGACGTGCAATGTTTGCCAATATTGCTCTCCATTACAAGCAAGGAACACCAATTCCATTAATCGACTACCTCGATTCAGAAGTAAACACTTGGAGAGTAGTTTATAAGAAACTTCAGAGTCTCTATGCAAAATATGCATGCAAGGAGTATCTGACTAATGTCCGATTATTATCCCAGCATTGTGGATACAATGAGGACAATATTCCTCAATTGCAGCACGTCAGCCAATTTCTTAAAGAGAGAAGTGGATTCACACTACGTCCTGTTGCAGGTTATTTATCAGCTCGTGATTTTTTGTCGGGATTGGCATTCAGAGTTTTTAATTGCACTCAATACATACGTCCTCCTTCAAAACCATTTTACACACGAGAACCCGATTGTGTCCATGAACTTTTGGGACACATGCCCCTTTTGGCTGATAGAATGTTTGCAGAATTTTCACAGGAGATTGGCCTGGCTAGTCTTGGTGCTTCAGATGATGAAGTTCAGAAATTAGCAAGTCTGTATTTCTTCACAGTCGAATTCGGACTCTGTCGTCAAGAAGGCAAAATAAAAGCGTATGGAGCTGGTCTACTATCGTCTGCAGAAGAATTTGAGATGGCAGTGACAACTCCAGAGAAGCAACGTGAATTTAATTTGAAACAAATCATGGAAATGGAATGTAAGGTGACTACCCATCAAGACTACTACTACATCACGAATTCATTCGACGAAGCCATTCGAAAGCTGCGGGAGTTCACGACTAGTATGAAACGGCCATTCGGCGTGAGATATGATCCGTATACTCAAAGCATTCAGAC

>CmacropygaSix3/6_Cmac.rna.tri.8155.1

ATCACAAAGAAATTCAACTAAGCGCTTCTTTGGTCTAAATCTAAATCTAAATACATTATAAATAGACTTCTTGATTTTCAGAAGAGCAGCCGCTAAAAATTCTGCTTTCTCTTCTTTTATTATCCACACCTTTTAAATTTCAACACTCCTTGTGATTTATTGGAAAATTTCACTTTTGAACTTGCTCATTTATTTAAACAGCTCTCCTGTTTTTCAGTTTGCTTCTTGTTTGTTTATCTCCACCATTCCAACAAACAAAACTAAACATAAATATCAAAATTTTGTCGTATTGAGCATTTCATGTTTCTTAGTTGTCAGTTCTTAGTTGATAATTCAAATTCTGTCGAAGTTAGTTAAAAATTAGACAAAATGCATACAGACCCCAAGTTAGGGCTTTTGCTGTCCCAAGCTCATGGGACCCCAGCAGCTTTTCCGCCTTCAGCAGCCGCGCTTTTCGCCGCCAACCCGATGATAGCGAACCACCCTCAACTCCTGCACCAACTCTCGGCACAACTGGCCGCAGCCCAGATGAACAACCCCGCAGGACCGGGACATGTATCTGGGCCGATGCACCCAGGCGCACCCTTGCCGCACCCAGCTTTGATGGGATTCAATCCTGAACAAGTTGCCCAAGTTTGCGATACACTAGAGGAAAGCGGAGATTTTGACCGACTCTCGCGGTTTTTGTGGTCGTTGCCTCCCCACTTGCTGGAAAGCACGATGAAAAATGAATCGATCCTGAAAGCGCGGGCAACTGTTTACTTTCACAATGGACAGTTCAGAGATCTTTACGTGTTGCTGGAAAACAACCGCTTCAAAAAAGACTATCACCCCAAACTGCAAGCTATGTGGTTGGAAGCCCATTATCAAGAAGCCGAAAGATTGCGAGGGCGACCTCTAGGACCCGTTGACAAGTACCGAGTTCGCAAGAAGTATCCCCTCCCCAGAACAATCTGGGATGGTGAGCAGAAAACGCACTGTTTCAAGGAGAGAACTCGTGGGTTGCTGAGAGAATATTACTTGACGGACCCCTACCCGAACCCTAACAAAAAGAAAGAGCTCGCCCAGCTGACGGGTTTGACGCCCACACAAGTTGGCAACTGGTTCAAGAACAGGAGACAAAGAGATCGCGCAGCAGCTGCCAAAAATAGAGGAACAGCACTGAACAATTTCAACTCTTCCGACAACGAGGGTTCATACGACAAAATAAGATCACTTGAAATTGACATCAATGACGACGAAGACGATGACGACATCGACCTTGACGTCAGCATATCCGATTCTGACGACGAGGACAGGGAAAACTCCTCCGACTTGAAGAAATCACCCTCGACAACCCTAACCCCTGACTCGACAAGTTCCAAAACCAAACTTGACGGCTCCTCAAATAAAGTTTGCAAACCAGAACCGTGCGATTTGAGTGGCGCCGAGCATACAACCGATTCGAGCTCGGCTTTGAAATTAAAGTCGGATAACTACAGCCCCGCAGCATCGACAACGTCCGAAGTCAGCCCGTCAAACTCAACAAAAAGTGGCTCATCTGTTTTTCCTTCGCCCACCCAACCGAATGGGGGTAACCCACTTTCTAGTTATTCCCACGCCGCGTTTTCAGCTCAGAATGAAAGTTTGAAACGGGAATTGTTTTCACTTTACACAGCTCAGCTTGGATCCCAACATCCGACAGGGATCCCGCAGACGTTCCCAGGACTCCACACATCTGCTAGTCCGATTCATTTGCCTAACCTCCCACCTTCGAGTCTCAGTTCAGCTGCAGTTGCCCACCAGCAACGTCTTTTAGTACCTCCAGCTCAGCTATTTCTGCAAGCACAATTGGCAGCTGCCGGACGTTTGAATCCTTCTCATCCGGGATCTAACCCACATTCCTTTCTAAACTCGACCAACGCGCATTCCTTGCTAAGAACAAACCAACTTTTAATAAGTTCAGCTAGTTTGAGTTCGTCTCAGTCAAAATCGAGCTCGAATAACTCGATTACTGCAGTTAGTACAACAACATCAATGACAACTTCTCCTGTAAGTTCAACAGTTGTGAACCCTACAACTACAACTAGCCCCACCAAACCCACAGTGTTCTCCCCCGTTCGTCTGTGCAATATAAAGGACTCCAAATAAATCAATTCTCGACATTCACACCGAACCAGAATCTCAACGAATCACACAGAGCAAATTTCTGGATGCTGAAAACTTTATAAAGAAGCTATTTTAGTACGGACTGATAATGCGTCTATATTTATTACCTACCGAAATTGTTGCAACTTTCGAGTTATATTGTTTTCCCCTTTAAAATTGTTACACGAATTTGGTGCCAAAAAATTGTACATAGCAATAACTTTTATTTGTACCCTTGTTGATTTGCTACCTAGATTTTAGTTGAATTAATTATCAAAGCCAAAGCTTAACTTCATGTTCATTTCACTCATAATTAAATGTTCACACCTTAAAAAAAAAAAAAAAAAA

>CmacropygaTH_Cmac.rna.tri.24852.1

TTTTTATTTAGTTCACTTAAAACAGATGTGAAGAAATTATCACAAACACATAAAATTTAAGGACTTTACTATCGTGTTGGATCTGATACAAGATCTTTGAGCAGTGCTATCTCAACAATAATCGTTATTCGAGCAGTTACAGTAGTAGATATTACTACAATGGCAGGAACAATAAACCCGAACTGCATCAAATCGCCTAGCTCGAAAATCGACCAAGTTTGTACTGCAGAAAACATACTGGAGTTGCCTGAACAAGGAGACACTGTTGGGCTAAAAAGAACGATGGCAGACGGTTCCTCCTGTCATAGGGTTGACAATTCGGGCTCTGAGTCTGCTGTGATTGGCCAAGCAAGTGATACGCAGTCACCAGAGAACAAGAAGAACCAGATTACATCTGTGGGGGCGGAGCAGGTGGAAGGAGAGGTGGAAGGGGTGGAACCAGTGTCACCCACCACCAGGTGGACTCAGATGAGATTGACATGGAAACACAAAGGGGACACCTCCACCTGGAGCACCTTCGACTTCAGGGCATCTTTCGATGGAACTAAGCGCGAAAGTTTAGTGGAAGACTCCAAGGCGGAGTTGGCGGCCAATAAGGAGAAAGAGAAAGTGGAATTGGCTCCTGGAGAGGAATCCACAACTCCGAAGCAGTTCAGAATTGAGATACGCAAATGTTCCTTGGCTCATTTGTCTTCGGTGGCTGCTATATTACACACAGAAAAAGCACAGCTTCTGGAGATAAGGACAGCATCAGATGACGAGTCACTGGTTTTGGATGAGCCAAAGAGCAGCCCCTCCTCTAACTCACTCACGGCTACTATCATAGTGGTCACAAAAAGACAGTTGTCAAAAGTTGTACAGTTGAGCAAAAATGTCACAGTAAAGGAAGAAATATGGTACCCTAGATGCGTTGATGATCTGAACAACTGCAAGAACGTTCTCTCGTCTTATGAACCCAATCTGGATCGAGATCACCCGGGATTCAATGATGTCAACTACAGAAACAGACGCGCAGAAATTGCAATGCTGGCTCAGAAATACAGACATGGAACCGATCCACCTCGGGTGGAATACACGGAAGTGGAGACTGCAACTTGGAAGGAGGCATTCGACAACCTCTCCCAGTTGTACACAACACACGCATGTATGGAATACCTCAAGTACTACAATCAACTCAAAGCTGACAACGTCATCCGAAACGACACCGTCCCTCAACTAGAAGATCTCTCCAAATATCTGCACGCTGCCACTGGGTTTACGCTATGTCCGGTGTCTGGCCTTGTTTCTGCCAGAGACTTCCTGGCTTGTCTGGCATTCAAGGTGTTCCCCTGCACTCAGTACATAAGACATCACGATGCACCCATGCACTCTCCTGAACCAGACCTCATCCATGAAGTATTGGGTCACGTGGTGATGTTCATGGATCAAAAGCTGGCCGACTTTTCCCAGAGAATAGGAAAGGCATCTTTGGGAGCACCTGATGAGTTCATTGTCAAACTGGCAACTCTGTACTGGTTTACAGTGGAGTTTGGTCTGGTCAGAGAAGGCGGTCAAGTGAAGGCATACGGAGCAGGTCTCCTATCTTCATACGGAGAATTAGAATACGCACTATCTCCTGAGCCTGATCACGTGACATTTGATACCTCAGTTGTGACAGTGCAACCATATGATGACTACAACTACCAGCAGGTCTACTTCATTGTTGACAGCTTCCAACACATGCTACACAACTTCGAGTCGTTCCTTCTTGCGTCATCAAACGAGAGCCGATTGCAACTGAAATTGGAGTGAATTTGCGCTGTGATGATTTGATATCATAGACGCTGAATTAGCATGTTAAATAACTGACTAATATTCTGTAATCTATCAATTGTACTCGAAACGGAGCGAACCCACGTTGAGGTGCCAGACCGAATGTCGATATGTCCAGACCGAAATGA

>CmacropygaPH_Cmac.rna.tri.2590.1

ATCTTGCCCTCTAAGCCGTGCTATCTATCATCTTAAACTAGAATTCGATTCATTTACTCAGAATTTGGATTTAGGTTTAATAGTTCATTTTTCAAATCATATTTTTGATTAATTCACTTGAAATTCAAAAGATTCAAAATCTATTCTTTTATATCAAATAGCCTAAAATAGTATTCAAACGTCTTAGAAATATTTCTGTCAATTGAAAAATATTTGAGCCATAATGGTGGTCGGAAATAAAAACAGACCTTCTGGCAGTGAATCTGGAAGCAATACTAGCATAATTTTTGGAATCAAAGAGAAAGTGGGCGCACTTGCAATTGCGCTTAAACCATTTGAAGCCAATGGAATTACGTTGAACCACATCGAATCGCGACCTTCGAAGAGATCCGAGGCAACTTACGAGTTCCTCATTACGACAGACAGCCAAGTCAATGACAACGAAGTGAAAGCGGCTCTGGGCGAGTTGACAGACAATGCGAGTTATGTACAGGTTCTGAGCAGAAGTGAGAGATCCCAGGAGTCAGTACCTTGGTTCCCGCGTAAGAAGAAGGACCTGGACCAGTTCGCTCATAGGATTCTGAGTTATGGATCTGAGCTTGATTCTGATCATCCAGGCTTCACGGACATAACGTACACAAAACGGAGGAAGATGTTTGCGGACATTGCCTTCAACTACCGTCATGGGACTCCAATTCCAAGGGTGGAGTATACAGCTGAAGAAATCAAGACATGGTCTGTCGTGCTGAACAGTCTCACAACTCTATTTAAGACTCACGCCTGTGCAGAGTTCAATTACATATTTCCTCTTCTGGTCACGAACTGTGACTACATTCCGGACAACATCCCCCAACTGCAGGACGTGTCTGAATTCCTCAAGAGTTGCACTGGCTTCTCAATCCGACCAGTGGCAGGACTATTATCGTCACGTGACTTCCTGGCTGGACTTGCATTTCGGGTGTTCCACTCGACGCAGTACATTCGGCACCCTTCCAAACCTCTATACACACCCGAGCCAGATGTTTGTCACGAACTGATAGGTCATGTGCCTTTGCTGTGTGACCCAACTTTCGCCAGATTTTCGCAGGAGATCGGAATTGCCTCCTTGGGAGCATCAGATGAGTGGATAGAGAGACTGGCCACCCTCTACTGGTTCACTGTTGAGTTTGGACTGTGCAAACAGGATGGCAAATTGAGAGCATTCGGGGCGGGGTTGCTGAGTAGTTTTGGGGAGCTACAGTATGCGTTGAGTGACAAGCCCGCAATCAAAGAATTCGACCCAGAAGTGACTTCAGTGACAAAGTACCCAATCACAGAATACCAGCCAACTTATTTCTACACACAGAGTTTTGAAGATGCACAAGAGAAACTGAAACAATTCGCTGCCAAAATCGCCAAGCCGTTCACTCTCCATTACGACGCATTCACGGAGTCCATTGACATTCTGGAAACAAAAGAGCAGCTAAGCCAACTTGCTGGACTGATAAAAGCGCAAGTGTCGACACTGCATTGTGCACTTGAGAGATTGGGATCGAGCGAATATTACGCAATTAACACTGGTAATCTTCAACAGTGATAGGAAGAACAAAATAGCAAGATGCGATTACCATTTGCAACATAAACCGGTTGCGAGTAGAACATTCATACTGTAGCTATATTTAATGACAGTTTTACTGAAAATTATTTAAACGTATCTCGATTTTTAGTCTTGTAGTTAAAAATCTCAGGATTCAAAAAAAAAAAAAAAAAAA

>TtransversaChAT_KY809754.1

CTGGACAACCTGTGTAGTCAGTTGAAAAGAATCACCAAGATGGCTGACAATTGCTACTCACATCCTATGGGAATACTGACAACCTTAGATAGGGATAAATGGGCTACAGCAAGACAAAGGTTACAAAGAGATAACACCAGTAAATCGTCATTGGATCTTATCGAGAAGTGTATATTTGTGCTCTGCCTTGATCGACATATACCCATATCATTTAACCACCAAAAGAGTATTGATCAAACAGATATGAATATACGAGATGTCAATTCTTTAGCTCTACAGATGTTACATGGGCAGGGTAGTAGGATTAACAGTGCCAACCGCTGGTATGACAAAACAATGCAGTTTATAGTGTGTGAAGATGGGGCGTGTGGATTGAACTATGAACATTCTCCCTCAGAAGGAATTGCTGTTGTGCAGTTAGTAGAACATCTGCTAACATACATGGAAGAAGTTCGAGTGAAGAAACTACAAAGAGTTCAGTCCCTATGTGAGATGCCCTACCCTAATAAACTACCGTGGAATGTGGATGAGGCGACGGTAGAAGATATAGAATATGCAAAGAGTCGTATAGATAAAGCAATAAGCGAGCTAGATTTTTATATGATAAAATTTGATGACTTTGGCAAAGAATTCCCAAAGAGGCAGAACATGAGTCCAGATTCCTTTATTCAGTTGGCATTGCAACTGACATACTACAAGGTTTACAGACGTTTGGTTTCTACTTATGAAAGTGCTTCCACAAGAAGATTTAGAGAGGGTAGAGTTGACAACATAAGGGCATGCTCTCTAGAAGCTCTTACGTGGGCAAAAGCTATGGTTGGCGAAGTTGAGGCGACGCCTGAAGAAAAAATTACCTTATTTAGAAAAGCAATAGAGCATCAGACAGAACAGCTTATCTCTACTATTCTAGGCCACGGTATGGACTGTCATTTGCTTGGCCTAAAGGAGCTTGCCACTGATATTGATCAGAACGTACCTGAAATCTTCACTGATGAATCTTATAGAATAACCAATCACTTTACCTTATCCACAAGTCAGGTGCCCACATCTACAGAATCCTTCATGTGTTATGGTCCAGTGGTACCAGATGGTTATGGTGTATGTTACAACCCCCATCCTAACTATATTGAATTCTGTATTAGTTCCTTCAAGGACTGCCAGGAAACAAAGTCGGATAACTTTGGATCCATCCTAGAATCAACCTTACAACAAATGCACGATCTCTGCTGTCAGACCAGTGATTTGATTGGACGATATGATGGTCGGGACGTTGGTGTCAACGGTGAGATAAGAAATGATCAAACTAGTGATGCCCCAAATAGAAGTCCTAGAGGCAAACTAAAAAGACAGCCTAACCTGGCTAATCATGTCTGA

>TtransversaVAchT_KY809753.1

ACCATGCCAAAAATCCTGGGAATAGGTTTTGATGCGTCAGAAATTATCCCGGCCATGAAGAAAAGGTTAAATGAACAACGACATCAAAAGCGGTTAATAATGGTGATCGTGTGCACGGCCCTTCTCCTGGACAATATGCTGTACATGGTAATCGTCCCTATCATACCACACTATCTCAAGAGTAAAGGTAGATGGTACACTCCTGCACCTCTTGGTACTGAATACACCAACATGACCACAGTAACACCATATGTCAATACAACAGTTGCTGAGGAGGCAACGGATATGACCAGTACGGTATCACTCAACACAACCACCAGAGAGCCATTGTTCTTTGCCAAGTACAATGCAGAAGACACAGGTGTACTGTTTGCATCAAAGGCCATAGTTCAGCTAATGATCAACCCATTGACAGGAGCCCTCATTGATAGGATAGGGTATGATATACCTTTGATGATTGGTCTGGGGGTCATTTTCTTTTCCACCATGATATTTGCCTTTGGAGAAAGTTATGCTGTGCTGTTCTTTGCAAGGGGTCTACAAGGAGTTGGGTCAGCATTTGCCGATACCAGTGGTCTAGCTATGATTGCTGATAGGTTCCACGAAGAGGCCGAAAGAAGTCAGGCTCTAGGTATTGCATTAGCTTTCATATCATTTGGGTGCTTGGTGGCTCCACCTTTTGGTGGGGTTCTTTATCAGTTTGCTGGAAAGGAGGTGCCATTTATAATCCTGGCAATGGTAGCTTTGGTTGATGGATTTCTATTATGGGGTATTGTTGGACCAATACGGGAAGAAAGGAGACTAAAGAAGGCCAATTCTGAAGTACCTTTAAATGGAACACCAATATGGCGTCTCCTGATGGATCCATATATAGCTATTACTGCTGGAGCACTGGCAATGTCGAATGTCTCATTGGCATTTCTTGAGCCTACTCTGGCCAACTGGATGGAGTCTACAATGGGCAGTAAAGAATGGCAGACGGGTCTGGTTTGGCTACCAGCTTTCATTCCTCATGTATTGGGAGTGGTTCTCACTGTTAAACTTGCAAAGGCCTACCCTAAGCACCAATGGCTGTTAGCTTTCATTGGATTGATGATGGAGGGCCTAATGTGCTTGATAATACCATTCTCTGGCGAATTTGGCGTTGTGATAATACCAATCATGGGTATATGCTTTGGTATAGCCTTGGTGGACACAGCTCTACTACCCACTCTAGGATACCTAGTAGATGTACGCCATGTGAGTGTATATGGCTCTGTGTATGCCATAGCAGATATATCATACTCCATGGCCTACGCCTTTGGACCTATCATAGCCGGCTCTGTCGTCGCAGCCATTGGATTCCTATGGTTAAACATAATAATATGCCTCACTAATGTGATTTATGCACCTTTACTGATCATACTTAAAGTAATATATAGATATAAGCCAGTGCAGGAGGAGTGTGATGTCCTAGTCAGCTCCGGGTTGTCACAGCAACAAGATTATAAGACATATATGGTTACCACCAAAGACGGAGAGAAGATGAAACAGGTTGAAGAAAAGTTGATGAACCATCTTGAATATTCAAATTCCAAAGAAGATATAACTAGGTCATCGTCATCATCTGACCTGGATAATACCACGCAAAGCAATAAAAAAACTGAAGTTCATCATGACAATGAGAAACTTGTAGAGGCAGTTAATCCGCTTTATAATCCAAATAATTATACACATCAACATGTAGGTAGGGGAGGTAAAGCCAAACGGGATAATAGTTCATCGGGGTCGTCAGATGATTCTGAATGGTAG

>TtransversaTH_Ttra.rna.tri.19142.1

﻿CGGCGGCCTGCAATGGAGAAGAAAGAGTCTTATCGACGATGCAAAGTTTGAAACGATTACCAACGTGGAATTTGAAAAACGAGAACGACATCTAAGTCAAAATGGATCGATCTCTGAGGATGACGTGTTTGAAACCAGCACTAGTGATGACATAGATCTATACGCGTTAGTAATAACATTAAGGGACGGAATTGTTACCCTCGGAAGGATACTAAAAATTTGTGAGAATTCCAAAGTTGCCATCCACCATGTAGAGTCTAGGAGCCAAAAAGATAGTGGACAAATTCAGGTGTTCCTTAGAGTAGAGTCAACTAAAGAGCATGTAGCTCATTTAGTGAAAACTCTGAGGCAAGGGGTCGCCGTTACTGATGTTATTGTGACTAATGACCATGATTCACAACGGAGAGATATCTGGATTCCGATACACATTTCAGACCTTGATAAATGTAACCATATAATTACAAAGTTTGAGCCAGAGCTAGACAGTGATCACCCAGGCTTTCATGACAAAGAGTACAGAAAAAGACGTCAACAGACGGCTAATCTGGCGTTTGAATACAGGCATGGAAAAACCATTGTCGACACTACATACACCAAAGAAGAAACGGAGACTTGGGGAGAATCGTATAGGATGCTAAAGAATCTGTTTCCAACTCATGCTTGTAAAGAGCACATTGACATTTTTGAGAAGCTTGAAAATGCGGGAATATACAGTGAACATCACATTCCGCAGCTTGAAGATGTGTCTACTTTCTTAAAACGATCGTCAGGATTTCAACTCAGGCCAGTTTCTGGTCTTTTGAGCGCAAGAGACTTTCTTGCAAGCCTTGCTTTCCGTGTATTCCAGTGCACCCAATACGTGAGACACCATTCCATGCCTATGCATTCTCCGGAACCGGACTGCATACATGAGCTGTTAGG

>TtransversaTpH_KY809752.1

GAAAATCAAGTTTCTTCTATTTCAGTTGGAATGCCGACTTGTTCGTTTGACAAAAATGGAAAAGAAATCAATAAGGTGCCATGGTTTCCCAGAAAAATATCAGAGATAGACAACACTGCCAACCGTGTCTTGATGTATGGCACAGAACTGGATGCAGATCATCCGGGATTCAAAGACAATGAGTATCGTAAAAGAAGAAAACACTTCACCGACATAGCTATGAACTATCGACATGGCCAACCTATACCTCACATTGATTATACAGAAGAAGAAGTCAAGACATGGGGGACAGTGTATCGTGAATTGAATAAGCTCTATCCCAAGTTTGCTTGTCGAGAACATATAAAAAATCTACCCTTACTAAGTCAGCACTGTGGATATAGAGAAGATAATGTTCCACAACTTCAAGATGTATCTGATTTCCTGAAAGAAAGAACAGGATTCCAACTAAGACCAGTTGCAGGATATCTTTCACCACGTGATTTTCTAGCAGGTCTCGCATTCAGAGTTTTTCACTGCACTCAATACATTCGACATGGATCAAATCCATTATACACACCAGAACCGGATTGCTGCCATGAATTGCTGGGCCATATGGCACTCCTTGCTGAACCAAGCTTTGCACAGTTTTCTCAGGAAATTGGACTGGCCTCTCTTGGGGCCACTGACAATGATATTGAAAAGCTAGCTACGTGCTACTTTTTCACGGTGGAATTTGGGTTGTGTAAGCAAGATGGGGACATGAAGGTTTATGGTGCAGGGCTTCTATCTTCTATAGCTGAGCTAAAGTATGCTGTCAGCGATAGGTCAGAAACAAAACCATTTGATCCAATTTCAACTAGTAAGGTTGAATGTCTTATAACAACATTCCAGCAACAATATTTTTACACAGACAGCTTCGAACAAGCTAAAGAGAAAATGCGATCATTTGCAAGTACAATCAAACGTCCATTTGCTGTAAGATACAACCCGTACACACAGAGTGTAGAAGTTTTAGACAATGCTCGGCAAATAGCTACAGTGGTAAATGAATTGAAAGGAGATCTATGTATAATAAGTGATGCTGTCAAGAAACTTGCCATCAAGGAATAA

>PharmeriSix3/6_MN431430.1

ATGAGTGCGGTCACTAACTTCTCAATGATGCAGCGCCTGTTGGCACCAGGCATGATGTTAGGGGCTGCCTACCCCATGTTCCCGTGCCTACCTACGCTGAACTTCTCCGTTGAACAGATAGCTCAAGTGTGCGAAACTCTGGAAGAGAGTGGGGACATTGAACGTTTGGGACGATTTCTGTGGTCATTGCCCGTCAATCCAACAGCATGCGAGGCCCTGAATAAACACGAGTCGGTGTTGCGGGCGAGGTCTCTAGTAGCCTTTCATACTGGTAATTTCAGGGACTTGTATCATATTTTGGAAAATCACAAATTCTCAAAAGACTCTCACGGTAAATTGCAAGCTATGTGGTTGGAGGCCCACTACCAGGAAGCTGAGAAACTACGCGGTCGGCCTTTAGGTCCCGTGGATAAATACCGTGTTCGCAAGAAGTTTCCCCTGCCAAGGACGATTTGGGACGGAGAACAAAAAACGCACTGTTTCAAAGAACGAACACGAAATCTTTTGCGGGAATGGTATCTGCAAGACCCATATCCCAACCCTACCAAAAAGCGTGAGTTAGCAGAGGGCACGGGACTGACGCCGACACAAGTAGGAAATTGGTTCAAAAACAGGAGGCAAAGAGATAGAGCCGCCGCCGCAAAAAATAGAATGTTACAAAAGCACAGAGCTGAACAGAAACGAAAGTGTATTGACGGAGATAATAACAATATGATAGATTCCTCGCCAGACTCCAAATTGATGGATTGTAAAGACGATGATCTCGACAGTAGTTTGGGTTCACCCATGAGTTCTTACGGCGACGAACCACTGTCCCCTGGCTCACCATG

>PharmeriTH_comp140338

﻿CCTTTTTGTCTCACGAGAATCATCGGTAACTTGTATTCGTTTGACGCTATATTCACAAAACTACTTTCATATTTCAAACATGATTTCTTCGTCTGATTCCGAGACCGCGGCGAGGCGACTCGCTTTCCAAAAAAGCTACAGTTTAGAGCATGGTAATTCATGGAAGCGACGATCTCTGATTGATGATGCTAAGTTTGATACCGTTACCAATGCTGAATTTGAGAAACAGGAACGCCGCCTTAGTCACGCCGACTCCCTGTCTGAGGATGAGGTGTTTCCATCTGAGACGAATGGTGAAATAAGCCGACTTCCTTCAGAAGGAGAAAGCGACACCCCGCGACAAGTAGGTGTCGCGTTCACCATGAAAGAGGGAAACGTCTCACTTGCTAGAGCATTGAAGACAGTTGAGAACTCGCATTTCACGGTTCACCACATCGAGTCGAGGAAGTCGAGTGTGATAGGTGCCCACATGGACGTCATCGTCATTGGCGAGAGTACGCGAGACCACGTGCTTACTTTATTGAAAACACTCAAACAGAGTTCTAACATTACTAATGTTAAAGTTCTCAATGAGAAGGACCCAGGCCTAGATATATGGTTTCCAACACACATATCTGAGTTGAACCTCTGCAACCACTTGGTTACGAAATTCGAGCCTGATTTAGACGACGGACATCCGGGATTCACAGACAAGGGATACCGAGCAAGAAGAAAGATTATAGCTGATGTCGCCTTCGAATACAAGCAGGGCGATCCTATACCCAGAGTTCATTACTCGGACGACGAAATAAAGACATGGGGCATAGTTTACAAGCAGTTGGTTGGATTGTTTCCGACTCACGCGTGTAGTCAACACATTGAAGTCTTCAAACTGCTCGAGAAGGAGTGTGGATACAGCCCAGACAACATTCCACAGCTGGAGGATATATCGAACTTCCTTAAAAGGAAATCTGGTTTCTGTTTACGGCCTGCAGCGGGTCTGTTGTCTGCCCGTGATTTCTTGGCTAGTTTGGCTTACCGCGTATTCCAGTGTACACAGTACATCAGACATCCATCGTCGCCTTACCATTCGCCCGAGCCGGACTGCGTCCACGAGTTATTAGGCCATGTCCCTCTACTGGCTGATCAGAATTTTGCTCAGTTCTCCCAAGAGATTGGTCTTGCTTCCTTGGGTGCGTCTGATGCTGACATTGAAAGATTCGCCACGCTTTATTGGTTCACTGTTGAGTTCGGATTGATCAAACAAGGCGGCCATATCAAGGCGTGTGGTGCTGGCCTACTGTCGTCGTATGGGGAACTCATTCACGCTCTGTCGGACACTCCTGAGAGGAAGCCATTCGATCCATACACTACTGCTCTACAGGAGTATCAGGATGCTGAATACCAACCTATATATTTCGTCGCCGAATCGTTTGATGATCTGAAGCAGAAAGTCAGGCTTTATGCTTCCCGCATCAAACGCCCTTTCGAAGTTCGCTACGATCCGTTTACTCAAACGGTCCAAATACTGGACAACAAAACAGCAATACAAGATATTACCAACGTCCTGAAGGCTGAACTTGACCATTTGAACACCGCACTTTGCAAACTAGACAACCCAGCATCTAATTTTATTTTGGAAAAACAGCTAGTGAAGAAAGCTATTAATAAATGTTGCTCATAAATAACTTGGACTTCATCTGCGACCACTTGTTGTGTAGGGATGTTACATTAAATCCCACCACTCAGATTAACGCCAACTCCCTCACGGAATATATGTTGCAACCCACGTCTGATCGTCACATGCTGATGTTGCTCGTCGTGTGGTACTCGCCTGAATATGGTAGTACGATTACAAACTGAAGATTACCGGCAAAGAACGATAAACTGGTCACTCTCAAAAGACTAGACACACCGAAACATCCCTTGACATGGCCGCTGTTTCGAGTGTGAGCGACACTGGTGTGATGAGCATCTCTTGGAGCAGAAAGTTAACAGACGTACATAGGTAGCAATGCAACTCAAACACATGTAGATAGTATTGATCTGTTACAACGGTCAGGGATTGGACCAGCTCAAGTTTTTGACGATGTTACATTATACAGATTATTAGATGAATGATAATATATCAACTCCACATGTAGTTTTCATGTGCAGGCATGGATGACGTAATACGTGCATTGACGTGGAGAAATATGTATATTCCGCTTTGTTATTCCAACTAATTCAAGAATGAGATACAGTGATCTCCACATACTTAACATGCTGCGCTGATTAGGCGCTAGAGTAGTTAGCTGAGGTTGGTAGCGATCACACCCATGCATGTATAGCAGCTTATAAATATAACCACCCTGTTACGATACACAATTGATGTGAACCCTTTGTGCATGTGTACCCAATGTCAGTCGTCATGAATAGTGCTTTCAAATAAATAAACGAATATTAAATGCCTCCAGTTTTATTTGTTGGGCCCAGATTCCATGGAATGAAAATACGGGCTGCCAAAACGCACGACTCTTTAGCAGTTGATTTATTGACAAAAATGTTTTTCCTAAAATGTCATATATATAGATAATAGAGGCGGTGGATCGGTCA

>PharmeriSyn_comp164939

﻿TGAATATTGATCAGGGAACTCATCTGAAGGCAAGACTGCTGTCATGTAGGTTGTAAGCCTGTCACGTCGCTAACGGGATATTTTAAATCCGCCTTTTTAACAACACCTGTGTTGATAGAACTGAGCCACAGGGCTGAGGTGACAAAGGGGTCCTTAGAACCCAACCCAGCCACTGGACTGGCTGTTTCAAAGGTTAACAGCTGATTCAACCTAGTGTCATGGAATATTTATTCCGAGAGCGGAGGGATGCAGACAATGCCGGAGCTGCCCCTGCTCCTGGGAACCCTGTCCCTCGCGGAGCGGAACCCCAAGTGGCCCCAGCAGGCAGCACAGCCCCCTCAGACATTGGTGGCGATACCACGACAATGGGACTGGATGTAACAACAGCCGCAAAAAATATGGGAGACAAAATAGCAGACAAATTTATGGAAGAGCTTCATAAACTACCATTGCCGGTATGGGCGGTCATTACAATTGCTGTTGTGGTACTTCTTCTTATACTGGTCATCTGCGTATGTATATGTAAGAAATGTTGCTGCAAGAAGCGGAAAAAGAAGGATGGAAAGAAAGGAGGAAAAGGCGTGGTCGACTTAAAGAGCGTCCAGCTACTGGGAAATTCTTATAAGGAAGGGGTTCAGCCTGACTTAGAGGAACTTGAGGTCAACATGGAAGACAACGAAGATGCAGAGAGCAAGAAGTCGGAGGTCAACCTTGGAAAACTGCAGTTTAAACTGGATTACGACTTTCAAAAAGGAGAGTTGGCTGTAACAGTAATACAAGCAGCGGACCTCCCTGGTATGGACATGTCTGGCACGTCCGATCCATACGTCAAAGTCTACATTATGCCGGACAAGAAGAAAAAGTTCGAAACCAAGGTCCATCGCAAAACCCTCAACCCGGTCTTCAATGAAGCCTTTACTTTTAAGGTTCCGTATGCAGAGATGGGCGGAAAAACGCTGACATTTGCGATATATGATTTCGATCGATTCTCCAAACACGACCAAATCGGTCAAGTGCTGATACCCTTGAATTCTATAGACTTGGGTCAAGTCGTAGAAGAATGGAGAGATTTAACGAGTCCTGATGATGAGGAAAAGGAAAACAAACTGGGTGACATTTGTTTTTCCCTACGGTATGTACCCACGGCTGGTAAGCTGACTGTGGTCATTCTGGAGGCAAAGAACTTGAAGAAGATGGATGTCGGTGGCTTGTCAGATCCTTACGTTAAACTTTCTCTGATGCTTAACGGAAAGCGTATAAAGAAGAAGAAGACGACAGTCAAGAAATGTACTCTCAACCCCTACTATAACGAGTCATTCACATTCGAAGTCCCATTTGAACAAATCCAGAAAGTTCAGATGTACATCACAGTGGTCGATCATGATCGCATTGGTTCTTCAGAACCCATTGGACGAGTCATATTGGGCTGCAATGCTTCAGGAACAGAGTTGCGACATTGGAGCGACATGTTGGCCAATCCAAGGAGGCCAATTGCCCAGTGGCATACACTTCAGGAATTACCAGAGAAGAGTTAAAAAGAGACTCCAACAATGTCAAGATATGGACAGTTTGCCTCATATTGTAAACTCTGAATGAAGGTTACACACGAGGAAGTCATGTTTAAATTGAAACTAAGTATCAGTCAGCCATTTGTTAGCAAATTAAAATAGTATTTCATTTAGGATTGACATAACATTATAAGGGCATCAAATGTTGACTGCTCTGAATAAGTGTGGCGCTTATCTTGGTTTTGCAAAATCATGATTCCTTTATAATTGGATCGTTGATGTTTTATGACAAATACCACACAGTCTGTCTTGGTCGTCAAATATCTATTTACATTCCTGACCTGCTAATTAGCTCTATCGAGTTGGTTGCTTACAAACCATTTTTTCTGCTTGCTATATTGAGGCTTATTACATCAAATTGATGGATGGTCAAACAAGGGCGTGGACTCTTAAAAGGAGTGGTGTGTTGTGAAGAAGTAGCCACCAACAACTTGGGTCATTCCTAGTGGTGCCATCTGGTGTAACCCATTGTGCACTGTAGCCCCCACTTGTTATACAAACTGTTACTATCCATCTGAAGATGTGACACGAAAAGCTGGTGAGAATATTTCTCCAAGGAAGTATGTTCTGCTTAGGAATTGTCCTAAACTAGGATGTCAATATTATATAAAGTGGTAGAAGCACAGCATTTCACGTCACGAATCATTTGGAATGATGCACACTTTTGAGACGGGTCAGCCCAGCCTACAACACCAAGTTTTTTTAAGCACGTTCTTGTACCACCCTAGCACCATCTCATTGTATGACTAGGCCCACGCTGCTTTGTGTTACCACGGTGATCAGAATTTTTTGTTAGCGTTGACCATTCTCAACCACAGCAATTTCATGTGGCAAAGTGCAATACACATTGTATTATAGTTAATATTGTAAGGTGATAATAAAATTAAACGATAAGAGGCTATATATATATATATTATATATATTTATTTACATCCACATTGTATTTCATAAGAAAACCTCGTGTTTGAGAATGTATTTGAAAATGAAAGCAGAATGTTATCCAATGAGAAGCCAGAATAGAACGAAATACTTTATAAATTCCAAAGTGCATGAGTGATGAAACAATTATGGACGGATAGAATTTTATGACTGTTGTCACAAGGTATTTGTCAGCCATTTTTAAAACAATTGTTCATGAAATTATGGGCGTATCTTAAGTTAGCAGCCATTTAAGTCAAGATGAGTACGCATGTTTTGAAATCGTGTCTTTCTGTTAAAGTACTTACATGCACTTGCACATAAAAGCACTCGTTGTCACCAATTCAGTTTTCTGTTATAAATGTTTTTTCTACATTATTTTGCTATTGTTTTGCATAGCAAACATCTATTCCTTTCTTGAATCTCTAGTTTGTGAAGAGTTTTGTTGCTGGACAAGTACAAGGAATGAAATGTTTATTCTACTTAAACCTTTTGTTTGAAGATATTATAACAAAGGATGACTTTGGATTAAAATCTAGAAAATCTTCATTCAGCTACATTCCTTCTTTATTTCCTTACCAAACATTCATTATTTAAATCTGTGTTGGCCCGCAGATTTGAGTTCCTCTCTTCTGTTTCCGTTAAGATTTTGGTGCATTTATGACGACGTTCTGAACAAATGAGCTCATGCGCTGTCCTTGTATAAGTCGATTACCAATCTTAGAAATTGCGGCTTAAGATTTAAAGTTTTGATTTGTGAGATATGCTACAAGCTTCAAATATTCATCTTGACAGGCAGTCAAGATGATGTGTTGAGTAGGCTTTACTGAGCTTGCTTAACTCGCCGTCTGACAGCTAAATCATAAGGTTTCAAAGTTGTTTGTTCTTAAAGTTTATTTTTCTTTATTTTTTCATCAAATTTTCTGTAGAAAGGACAACAGACATGCTGCAGGCACTAACAAAAATGTCATTAATGTCTTCAGAGTTCATAAACGTGACATTTCAGGTTAACCAAACAACACAACCATTTATGTTAGCAATATGTCAAAACCTGATGCTTAAAATGCCAAATGCTGTTGTTGCATCGGGACTAGACAGTTTTGTAGCCCATGTCTTATTTATTGTGATAGCCTCTTAATTACAGTATCAGGCCAATTATTGTGAATTTTCATTATTTTTTAGAGTTTAAAGTTTGTTTGGAGCAGGGTTACAAAATGTCTGACTTCTGTCCATCATCAGAAATTGACATGTACTGTTAGGAGACTTGAATAAACACCAGTATTTTTGCTGGAAATCAGTTTTAAATGGGTCACCATGGTTTTGAGTTTTAACAAGCCATTGTGTTGAACACAGCTCAAGGGCCATGCCTCTTGTTTAGAAATAAGTTATGAGATTGGTAATGATGGTTGGAATTTTCAGTAAACATTTTGTGTCCCTGCAGATGGCGCTGTGATAAGGTAAAACATTTTATTCTTCTGCTTGGTGAGAGATTGAAGGGCTCAATTGTCGCAAACAATAGGATTATATTTATCGCTTTGGACATTTGGGTTGTCAAATTACATATAACATATATATCTATGTAGCTAGTTTTCTTGCGTTTCAGCATATGTATCCTTCAGTTTATACAAATCGGAAACGGTAGTTTTTTAATGTTTTTGCATCAACCTATCATGATAATGGTTAGTGCAACTATGTTTTCAAAAAAAACTTACCCGTGTTTAGTTTTTTAGCAATTTGTGAATAATAAATTGTTAAATAAAAGAACTTGTTATTGACAGTTTCAAATACAAACTGATGGAATATATACTTAACTCTTTGGAATCTTGACGTGCATTTATCACAAGTGTAGCTTTGGTGTTTAAGGTATCAGGAAGCCTATCGAAGCAACTGCTTTTCGTCTTTTTAGGCAGCATCAGTTACAAATACTGTCGTATGCAATTTCCCCTTGTAGGATCCATTAATCGTAATATCCCCCTGGGGGAAAATGTCTCATTTCCAAAACAATGATTTATTTTGTTTGTGAGTGGTTTTAACGATGATGATAAATAGTAGCGTGTTTATGTATAGGTGAAATAATGGAGGTATCGTTTAATCAACTTATATAAAATATAGGTCACAATCACCGTAATACTTTGTAGTTGTAGCATAAGGATCTCTCACCCTAAACTCGATGTGTGGTAGGTAAAGTTTAGTCATGTGTAAACAGGGTTTAATTGTTCTGCAATTCTGACAAACTTGCCTATCCTCCTTAGAATACGAGGAGTGGAGAATGAATTGACAATGAACTAATACCCGTTTATTCCTAACTAAACGATACATGTATGAAATATAGAAAAAAATCAGTGAGGAACAGCATTTCGGTTTCTGTTGGAAAGACTTGATGTTCACCTTTCATTGTGCATGTCTAGTTTTAACTTTCAAAAAAAACTTGGAATGCACCTTTGTCACTGAGAATTTGATTATCAAATAATGAGAGATATGTTATGAAGATATGACATACCATGTGTTATAGGAGGAACAACGTTTTGAAAAAACACTAATGAGAATATCCTATTCCAGTGAGTAGTGAATGTGTGAACATCAATGTTTTCCTGACAAAAAGGTTACTTGAACATTCACTGGAGGTGGATGCAAAACATGGAATGTGAAAATGTATGTTGTGGTGTATAAAATTATAAAAAATAAAAATAGTGTTGTTTTATTTTAAAAAAAAAA

>PharmeriGata_MN431425.1

ATGGCACACACTGATTCGAACTGGCTACACTCATCCTTCACCATACCAAAGCTTCACAAGCCCTCCCCCGACACAGACGACCCAAGCCACCTTCTCCCTAAGGAAGACGTCGAGAACTTTTTCAATCATTTAGAAGAACCACTACCAACTAGACTTCCAAGTATGTTCCAGAGCAGCGTTCCAAGTTCTTTGCCGACCTACGACAACGCCCATGGCGGTTACATGCCGACGGCGAGTCCAGTCTACGTACCCACCACAAGGACTATGTTACCGGTCCAGTATATGAATGGGTCGGCGCAAGCTGTCCCACAGAGTAACAATTCCATGTGGTCGATGCCGGCTGATCCCGCGTACAGTGCTGGCGGTTCCAATGGACGATTTGCGTTCCCTCCCACGCCCAGTCCCCCTATCGCGTCACCAACCGGCCGAACGGACGCGTACGGTGCCATGCCCAGAGGAGCGGGACTGAGCCCCTACCCAAGCTATCCCATAAGTGATTCAATGTCAGCATGGAACGCGTATAATAACCAGATGGGAATGCAGCAGGGAGGTCTACGGAGGGCGACACTACCCCCTATGCAAGAGGGTGATTTCTTTGGCGAAGGCCGTGAATGCGTGAACTGTGGCGCTATCTCTACCCCTCTGTGGAGGCGAGATGGGACCGGTCATTACCTGTGTAACGCCTGCGGACTCTATCACAAAATGAATGGACTCAACCGACCTCTTATCAAACCGCAGAGAAGACTCTCCGCCTCACGCCGCGTCGGCTTATCATGCGCCAACTGTCACACATCCACCACGACGCTATGGAGACGTAACAACGAGGGTGAACCTGTTTGTAATGCATGCGGTCTCTACTACAAACTCCATGGGGTGAATCGACCACTGGCAATGAAGAAAGATGGCATACAAACAAGGAAGCGTAAACCGAAGACAATGGTTAAGGAGAAGGCCATCAAGTCTGAAAATCCAGATCCAAAGCCACAGGGCATGAACACTTTGCACGTAAGCCAACATATACAGCAGAATATCAACTCGAGTGTCATGTCGAGCGCATCTTTACACGGCATGATGTACGCAAGTCAACCCCACTCTCTACCTCACATGTCTGAACACAGCAGCACTCCTTTATCGTCGGGATCGTCGAACGCCTTTCCTACACCTTCTCCTCCCAAAGCTATTCCGGTTTCCAATGGTAGCGATAACGGCAACGTGAACAACCTCAGCGACCATGCGTCGTTAACTACCGTCTCCGTAGGGGCTAGTTA

>OfusiformisSix3/6_KR232531

ATGTTCGTTTGTGCCGATCCAGCTCGGACTTTGGCCGCAGTATCAATGGCAACGAAACAACCGATGAAGGTACCAACAATTACGGCGGTACCAAGCCCGGCAAATATGTTCATGGCTTTGCCAATGTTAAATTTCACACCACAACAAGTTGCACAAGTTTGCGAAACTTTGGAAGAAAGTGGCGATATTGAACGCCTTGGACGTTTTCTATGGTCGTTACCCGTTAACCCGGGCGCCCTGGAAGCCCTCAACAAATGCGAATCAGTACTAAGGGCCAGAGCCCTTGTAGCCTTCCATACGGGAAATTTCAGAGACCTTTATCACATCTTGGAGTCGCACCGATTTACAAAGGAATCCCATGCCAAACTGCAGGCTATGTGGCTCGAGGCTCATTACCAGGAAGCCGAGAAACTAAGGGGAAGACCCCTCGGTCCAGTGGACAAGTACCGTGTCAGGAAGAAGTTCCCATTACCAAGGACTATTTGGGACGGGGAACAAAAAACACATTGTTTTAAAGAAAGGACTAGGGGACTTTTAAGGGAATGGTATCTTCAAGATCCATATCCCAATCCTACAAAAAAGCGGGAGCTAGCACAGGCCACTGGTCTAACCCCAACACAAGTCGGCAACTGGTTCAAAAATAGACGACAAAGAGACAGAGCAGCAGCAGCCAAAAATAGATTACATCTAAACAGAAGTCACTCATCATCCAGTATGGATGGGGTCGGAGATCACGACAGAGACAGTAAATCCCCGCCCATGTCCCCTCTATCGATGACGTCAGACGAAGATGACCTATGA

>OfusiformisGata4/5/6a_KR232537

ATGGACAGTGACACCAACTGGTCGGCAGCCACTAGCTCGCACGCTCAATCAGAGATTCAGCCAGCTCGATACGAAAGACCAGGGTCCTCACCACGGGACCAACTTGTTATGCAGGAGAGACCTACTGCACAGAACGCATTGTATCCACTACCCAAGGAAGAAGTTCAGTGTTTCTTTGATCAGCTTAGTGAACCAAATCAGCCCATCCCCGCAAGCACCCTGGCTACCCTAGAAGACCGCAGTATGTTCCAGCAGCCAATGACGGTGGCTAGTCACGCCCCGCCTACCTACCACCACGAAGGTAGCAGCTATTTGCATTCAGCAGCAACCAACCCTGTCTACGTACCAACAACACGGGCTACATTGGGATCCATGCTACCTATGCAATATATGAGCAATGGAACTGGACAAGGATCGCCACAGGGTTGGCCAACACAGGACAATGGCTATTCAACTGCCAGTACCCATCCTTCCATGTCACCTAGATTTACGTTCCCTCCAACCCCAAGTCCCCCTGTCAGTTCTCCAAGCAGTCGCACTGACCCCAGTTACAGTGCACTTACACGGCCTACAGGAATCAGCCCCTATGGTTACCCTGACATCTCTGCCTGGAGCAGCTACAACAACATGGCGTTGTCACCGCAGCAAGGTCTTCCAAGACGCCCCAGTGCAGACCCTGTTGTAGACCCATACGCCAGCAAACACTTGGAGTGTGTCAGATGTCGGAGCGTCTTGTCATCAGCGTGGCCCCAAGAAGGAATGGAGTACATCCAATGTACTATGTGCGGACTCTACCAGCGCCTCAATGGCTTCAACCACGCCATGGCCAAGACCGGCCCCATCAGGAGCTCTAGGCTGTCTGCATCAAGAAGAGTCGGACTTTCCTGTGCAAACTGCCATACTTCACAAACCACATTATGGAGGCGGAACAATGAGGGAGAACCTGTATGCAATGCATGTGGCCTCTATTATAAACTACATGGGGTCAATAGGCCATTGGCAATGAAGAAGGAAGGAATCCAAACGAGAAAGAGGAAACCAAAGAACATGGGAAAAGTCAGAAGCCCAATGAAGTCAGAACCGTCTTCAGATTTGAAGACCTCCGTTAGCTCACCATCAATGGGATCTCTGCACCAGGTCCATAATTCCCAGCAGAGTGCTGCATCAGCCATGTCCGTCATCCATAACACCGGCAACCACGGTCTCTCCGTTAACAACATGATGCTACCAGGCAATACTAACATAAGCACTCCAGATCATAGCCCTAATATAGGCAGTCCAGGTGCTGGTCTCAATCCGGCATCTCAAACTCAAAGTGTCTTCCCTACTCCCTCGCCTCCTAAAGCAGTCCCCGTCAAAATGGAGCCTGGGACTATTATGACACCCTCGCATCATGAAACTACCACCCTAAGCTCAGTCTCTGTGGGAGGAAATTGA

>ScalifornicumSyn_SCA31175

ATGGCGGATGGGTTTCTACTGCGACGCTTACTGGAAGCAGCGACAGGGGCAGCTGATGTCAGCGATGACACGGCTACTGGAGGGGGAGACACAACAGATGGAGGTGGTGGGGGCGGTGGTGGTCTTGATTTGAACCGAGGAATAACAAACATAGGTAACAAGTTATATGACAAGCTGAAGGAACTACCATTGCCCATGTGGGCCATCATAGCTATCGGCATTGTGGCAGGATTACTCCTACTTTGCTGTTGTATATGTATATGTAAGAAATGCATCTGTAAGAAGAAAAAAAAGAAGGAAGGAAAGAAGGGTTTGAAGGGTGCTGTCGATTTGAAGAGTGTGCAAATGCTTGGAGCTAGTTATAAGGAAAAGGTCCAGCCGGATGTGGAAGAGCTAGATGGTGCTGGAGGAGAAGATCAGGAAGACACGGATTCTGTCAAGTCGGAACTAAAACTGGGAAAGTTACAGTTTTCATTGGACTATGACTTCCAGGAAGGAAAGCTAACTGTTGGCGTGATCCAGGCAGCAGACCTTCCAGGCATGGACTTTTCAGGTACTTCGGATCCCTACGTCAAAGTTTATCTTCTACCTGACAAGAAGAAGAAATATGAAACTAAAGTACACAGAAAGACACTTAACCCAGTCTTCAATGAATCATTTACATTTAAGGTTCCTTACAGCGATGTTGGCGGCAAGATCCTGACCTTTGCGATCTATGACTTTGATCGTTTCTCACGACATGACATCATTGGTGAAGTCAAAGTACCGCTCAGTTCTGTGGATTTGGGTCGTGTTATTGAAGAATGGCGTGATCTACAAAGTGCTGAAATACCTGGTGGAGAGGGCAAGTCTGAGCTTGGCGATGTTTGTTTCTCTCTTCGATATGTTCCCACTGCCGGTAAATTAACAGTTGTGGTTTTGGAAGCCAAGAACCTCAAGAAGATGGACGTTGGTGGACTATCAGATCCATATGTGAAGCTCTCTGTGTACATGGGTGGCAAGAGGATGAAGAAGAAGAAGACAAGCATAAAGAAGAGAACTTTGAATCCATATTACAATGAATCATTTGTCTTTGAAGTGCCCTTTGAGCAGATCCAGAAAGTAACGCTGGTTGTGACTGTGGTAGACTATGACAGAATGGGAAGCAGTGAACCAATTGGTAAAGTTGTACTGGGCTGCAATGCATCTGGTGCTGGTCTTCGTCACTGGAGTGACATGTTAGCATCGCCACGACGACCAATCGCTCAATGGCACACATTGGTTGAACCTGATACATAA

>ScalifornicumSix3_KX845335.1

ATGTTCCAGCTACCGACTCTGAACTTCAGCCCTCAGCAAGTTGCGAGCGTATGTGAAACGCTAGAAGAAAGTGGCGATATAGAACGGCTAGCCCGATTTCTGTGGTCGCTACCAGTCGCCCCGGGAACATGCGAGGCACTCAACAAGAACGAGAGTGTCCTACGCGCCCGGGCGGTTGTAGCGTTCCACCAAGGTAACTTTCGAGAACTATATAGTATATTGGAAAACCATAAGTATTCTAAGGAATCACACGCCAAATTACAAGCAATGTGGCTCGAAGCACATTACCAAGAAGCTGAAAAATTACGTGGTCGGCCGTTGGGTCCAGTCGATAAGTACCGTGTACGGAAGAAGTTTCCGCTACCGAGGACAATATGGGACGGTGAACAGAAAACGCACTGCTTCAAGGAACGAACGCGGAGTTTACTACGTGAATGGTACCTTCAAGATCCATACCCTAACCCAACTAAGAAGAGAGAACTAGCTGGAGCAACTGGCCTAACACCAACTCAAGTCGGAAATTGGTTTAAGAACAGGCGTCAACGAGACAGAGCAGCAGCGGCCAAAAACAGGCTTCAACATCAAGTACCACAATCAACGTCACCGAATCCTATAACTATAGATGGTGCCACCACTTTGCCAGGACAGCAGCCGAAACCGCGGCCGCTCGATCCAGCGCACGCACTACTCGAAGCCACCCAGAACAAGACACAGTCGCAAACGCACGTATAG


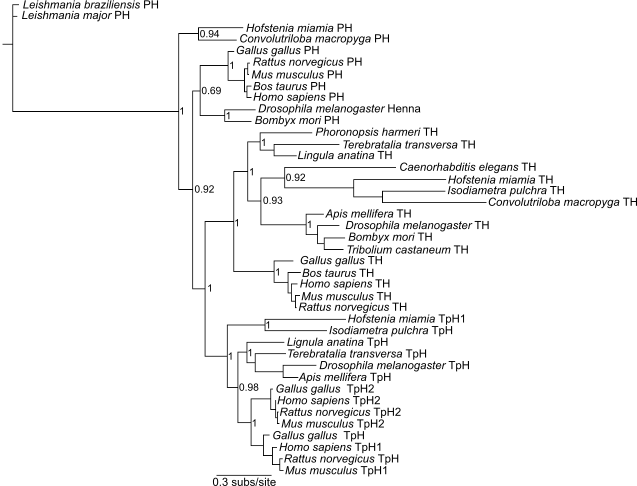


Aromatic amino acid hydroxylase gene tree (PhyML, default settings, aBayes branch support for most internal branches shown, Guindon et al., 2010) showing the relationship between previously analysed genes and genes used as cell markers in in situ hybridization. Leishmania PH were included as outgroups based on previous work (Siltberg-Liberles et al., 2008).
