# Supplementary material for: Peripheral and central employment of acid-sensing ion channels during early bilaterian evolution
Source: eLife. 2023 Feb 23;12:e81613. doi: 10.7554/eLife.81613 (PMC9949801; doi:10.7554/eLife.81613)
Supplement: Supplementary file 3. [file elife-81613-supp3.docx]

**Supplementary File 3**

Modified pSP64 vector and inserts used for *Xenopus laevis* expression and electrophysiology

Sp6, HindIII, 5'UTR, SalI, XbaI, BamHI, Myc + TAA stop codon, 3'UTR, poly(A), EcoRI

**Modified pSP64 Vector**

ATTTAGGTGACACTATAGAATACAAGCTTGCTTGTTCTTTTTGCAGAAGCTCAGAATAAACGCTCAACTTTGGCGTCGACTCTAGAGGATCCGAGCAGAAGCTCATCAGTGAGGAAGATCTCTAAGGTTACCACTAAACCAGCCTCAAGAACACCCGAATGGAGTCTCTAAGCTACATAATACCAACTTACACTTTACAAAATGTTGTCCCCCAAAATGTAGCCATTCGTATCTGCTCCTAATAAAAAGAAAGTTTCTTCACATTCTAAAAAAAAAAAAAAAAAAAAAAAAAAAAAACGAATTCGTAATCATGTCATAGCTGTTTCCTGTGTGAAATTGTTATCCGCTCACAATTCCACACAACATACGAGCCGGAAGCATAAAGTGTAAAGCCTGGGGTGCCTAATGAGTGAGCTAACTCACATTAATTGCGTTGCGCTCACTGCCCGCTTTCCAGTCGGGAAACCTGTCGTGCCAGCTGCATTAATGAATCGGCCAACGCGCGGGGAGAGGCGGTTTGCGTATTGGGCGCTCTTCCGCTTCCTCGCTCACTGACTCGCTGCGCTCGGTCGTTCGGCTGCGGCGAGCGGTATCAGCTCACTCAAAGGCGGTAATACGGTTATCCACAGAATCAGGGGATAACGCAGGAAAGAACATGTGAGCAAAAGGCCAGCAAAAGGCCAGGAACCGTAAAAAGGCCGCGTTGCTGGCGTTTTTCCATAGGCTCCGCCCCCCTGACGAGCATCACAAAAATCGACGCTCAAGTCAGAGGTGGCGAAACCCGACAGGACTATAAAGATACCAGGCGTTTCCCCCTGGAAGCTCCCTCGTGCGCTCTCCTGTTCCGACCCTGCCGCTTACCGGATACCTGTCCGCCTTTCTCCCTTCGGGAAGCGTGGCGCTTTCTCATAGCTCACGCTGTAGGTATCTCAGTTCGGTGTAGGTCGTTCGCTCCAAGCTGGGCTGTGTGCACGAACCCCCCGTTCAGCCCGACCGCTGCGCCTTATCCGGTAACTATCGTCTTGAGTCCAACCCGGTAAGACACGACTTATCGCCACTGGCAGCAGCCACTGGTAACAGGATTAGCAGAGCGAGGTATGTAGGCGGTGCTACAGAGTTCTTGAAGTGGTGGCCTAACTACGGCTACACTAGAAGAACAGTATTTGGTATCTGCGCTCTGCTGAAGCCAGTTACCTTCGGAAAAAGAGTTGGTAGCTCTTGATCCGGCAAACAAACCACCGCTGGTAGCGGTGGTTTTTTTGTTTGCAAGCAGCAGATTACGCGCAGAAAAAAAGGATCTCAAGAAGATCCTTTGATCTTTTCTACGGGGTCTGACGCTCAGTGGAACGAAAACTCACGTTAAGGGATTTTGGTCATGAGATTATCAAAAAGGATCTTCACCTAGATCCTTTTAAATTAAAAATGAAGTTTTAAATCAATCTAAAGTATATATGAGTAAACTTGGTCTGACAGTTACCAATGCTTAATCAGTGAGGCACCTATCTCAGCGATCTGTCTATTTCGTTCATCCATAGTTGCCTGACTCCCCGTCGTGTAGATAACTACGATACGGGAGGGCTTACCATCTGGCCCCAGTGCTGCAATGATACCGCGAGACCCACGCTCACCGGCTCCAGATTTATCAGCAATAAACCAGCCAGCCGGAAGGGCCGAGCGCAGAAGTGGTCCTGCAACTTTATCCGCCTCCATCCAGTCTATTAATTGTTGCCGGGAAGCTAGAGTAAGTAGTTCGCCAGTTAATAGTTTGCGCAACGTTGTTGCCATTGCTACAGGCATCGTGGTGTCACGCTCGTCGTTTGGTATGGCTTCATTCAGCTCCGGTTCCCAACGATCAAGGCGAGTTACATGATCCCCCATGTTGTGCAAAAAAGCGGTTAGCTCCTTCGGTCCTCCGATCGTTGTCAGAAGTAAGTTGGCCGCAGTGTTATCACTCATGGTTATGGCAGCACTGCATAATTCTCTTACTGTCATGCCATCCGTAAGATGCTTTTCTGTGACTGGTGAGTACTCAACCAAGTCATTCTGAGAATAGTGTATGCGGCGACCGAGTTGCTCTTGCCCGGCGTCAATACGGGATAATACCGCGCCACATAGCAGAACTTTAAAAGTGCTCATCATTGGAAAACGTTCTTCGGGGCGAAAACTCTCAAGGATCTTACCGCTGTTGAGATCCAGTTCGATGTAACCCACTCGTGCACCCAACTGATCTTCAGCATCTTTTACTTTCACCAGCGTTTCTGGGTGAGCAAAAACAGGAAGGCAAAATGCCGCAAAAAAGGGAATAAGGGCGACACGGAAATGTTGAATACTCATACTCTTCCTTTTTCAATATTATTGAAGCATTTATCAGGGTTATTGTCTCATGAGCGGATACATATTTGAATGTATTTAGAAAAATAAACAAATAGGGGTTCCGCGCACATTTCCCCGAAAAGTGCCACCTGACGTCTAAGAAACCATTATTATCATGACATTAACCTATAAAAATAGGCGTATCACGAGGCCCTTTCGTCTCGCGCGTTTCGGTGATGACGGTGAAAACCTCTGACACATGCAGCTCCCGGAGACGGTCACAGCTTGTCTGTAAGCGGATGCCGGGAGCAGACAAGCCCGTCAGGGCGCGTCAGCGGGTGTTGGCGGGTGTCGGGGCTGGCTTAACTATGCGGCATCAGAGCAGATTGTACTGAGAGTGCACCATTCGACGCTCTCCCTTATGCGACTCCTGCATTAGGAAGCAGCCCAGTAGTAGGTTGAGGCCGTTGAGCACCGCCGCCGCAAGGAATGGTGCATGCAAGGAGATGGCGCCCAACAGTCCCCCGGCCACGGGGCCTGCCACCATACCCACGCCGAAACAAGCGCTCATGAGCCCGAAGTGGCGAGCCCGATCTTCCCCATCGGTGATGTCGGCGATATAGGCGCCAGCAACCGCACCTGTGGCGCCGGTGATGCCGGCCACGATGCGTCCGGCGTAGAGGATCTGGCTAGCGATGACCCTGCTGATTGGTTCGCTGACCATTTCCGGGTGCGGGACGGCGTTACCAGAAACTCAGAAGGTTCGTCCAACCAAACCGACTCTGACGGCAGTTTACGAGAGAGATGATAGGGTCTGCTTCAGTAAGCCAGATGCTACACAATTAGGCTTGTACATATTGTCGTTAGAACGCGGCTACAATTAATACATAACCTTATGTATCATACACATACG

**ASIC inserts for expression in *Xenopus laevis* oocytes**

>IpulchraASIC_Ipul.rna.tri.12910.1

GTCGACATGTTACCAAGCAATCAATGGGCCATCACTGTGAATGCGACGCCAGCCCACCGCGTTGGGTCCGAGTATGATCCGCAGTACAACCCGCAGACCGCACGGGGGCCGGAAACCCAGGTGGTGCGGTATGCGGCGCCGGTGCGGACCGTGCTGGTGGACAGCTACTGCCAGACCAGCGCTCCGCCCTCCGCCAGCTGTCTCCCCATTGCGGAGCGGTACCAGTGCGCCATGGCCAGTGTCCAGTACAGCGAGGACGAACACTCCATAGGAATCAAGACATTCCTATCCGAGGAAACCACCATGCACGGCATCAAATATGTGGTGCTGCCGGGTGCGCGGATCACGATCAAGCGGGTGCTGTGGACGCTGGTGTTCGCCCTCTTCTGGGTCTACTTCATCTCGGTCTCCGTCAAGAAGGTCCAGTACTACTACTCCTTCCAGCACATCACCCAGGTAGACGAGGAGTCCAAGGAACTCAACTTCCCGGCCATCACGATATGCAACCTGAACCCGATCCGGCTGTCGGCGTTCACCAAGAGCGACCTGTACCAGGCGGGCCGCATGTACCAGATCCTGTCGGCCAACCTCACCCTGAAGGAGGACCTGTTCTCCTCGGGCGACGAGACCGCGGTCGCCCTCCGCTCCAAGACCGACTTCACCGAGCAGGACAAGCGCGTCCCCTTCGACCTCGAGGAGTTCCAGCTGCGTGCCGGCCACAAGATGAAGGACATGCTCAAGGCGTGCAAGTGGCGCGACCAGCAGTGCACCGAGAAGGACTTCCAGCCCGTGCTGACGGAAATGGGGCTGTGCTATCAGTTCAACAGCAACAAGTCGCTCGACCCGGAGACGGGCGAGTACGCGTTCCGCAAGGTGTACAAGGGCGGCGCCGGCAACGGACTCAAGCTACTCCTTGTCACCCAGGAGCAGGACTACCTTCCGCCCAACATCGAGGACTTCGACTCGACGCTGGAGACGGGGTTCAAAATCGAGGTGCACGACCCGGACGTGCCGCCGCTGGTGCACCAGCTGGGCATCGGGCTGGCGCCCGGGCTGCAGACGTTCGTGGTGCTGCAGAAGGAGGAGGTGAACTACCTGTCCGAGCCCTGGGGCTCCTGCATCGACCCCATGGAGCCGGGCAAGGTCACCAACTTCACCAAGTACTCGGACCCCGCGTGCCGCATCGAGTGCGAGACCGAGCACATCGTGCAGGAGTGCGAGTGCCGCATGCCCTTCATGCCCATTTTGCCGGCGTACGAGAACTCGAAGACGGTGTGTTCACCCGCGCAGATCATCAAGTGTGCGCGGTCCGCCCTGGCGCGGATAGAGGACGGAGAGGCGTGCGGGCCGGAGACGTGCATCAAGAGCTGCCGCATCTCCCGCTACACCAGCTCCCTCTCCTCCGTCAAGTTCCCCAGCTTCCTCGGGGCCATCAAGCTGCAGGAGGAGTTCCCCGACACCTTCCCCACCAAGAAGGACGTCACCCCCGCCAATGAGTCATCCGCGGCCGCCGCCTCCGCTCCCGTCACCGGCAAATGGAAAGAGACCATAGACGGAGCTGCGGCCCAGAAGAAATGGGAGCAGGCGCTGGTCGTGTCCATATACTTCCAGTCGCTGAGCTTGCAGAAGATCGAGCAGGTCGAGGCGTATGACTTCCCGGCCATGCTCGGCGACATCGGAGGTCTCATGGGGCTGTTTATCGGCGCCTCCGCCATGACGCTGTTCGAGTTTCTCTTCTTCTTCTGTGACTCTCTCATCCTCCGGCGCAAGAGCACCAAGCAGCCGCCGCCGGACACCTCGAGCCAGAGCTCGCCCTCCAGCGGCCAGAGGAAGGTTGTCCACGCCGAGATGGACAACGGCAAGTTCTCCATGCCGCCCGGCGCCGTCGATGGCTCCAAATTCGTCATCAACTTCAACCCGGACTACAATCCGGGCATTACACGAGTGCTTCCGCAATTCACTGCTCATCCTCTGCACAACAGCTTCCAGCAGCAACAACAGCCGCAGCAGCAGCCGCGCGGCTACTCACCTCTGATGAAGCGGGCCGCCGGCAACTTCGGAGCAACCGACGGCGGCGATGAGTCATTCGGCCAGCAGCCCGCGCCCGCCCAGCAGCCGCAGTTCAACACTCGAATGTACCAGCTGCCCAACAACCAGCCACAGGAGCGCTACTGCGGATCC

>HmiamiaASIC_98006429

GTCGACATGAATAGGCCAATGAAATACGTCGCCACTCAACCCGTTGATCAACCCCCTCAATGCACATTCATTGATTCATATGCCGATCATGATCGAAAATCTCTCCTCGATTTTGCCGATGAAACAACAATGCATGGAATGCGTTATGTGGTCTTGCCGGGCGCGAAAATTACCATCAAACGCGTGCTATGGACTTTTGTTTTTGCGCTTTTTTGGGTGTACTTTATTTCAGTTAGTATTAAAAAGGTCCAATCTTATAGGAAATACCAGCATATCACCCAAGTTGACGAAGTATCCAATTCCCTTCCTTTTCCCACTATCACAATTTGTAATTTAAACCCAGTACGGATATTTTCTTTCACAAAAAATGATCTTTATCATGCCGGTCAGATGTATGAACTACTAGATGAGAACATGACGTTGATCAAAGGACTCTCCAAGAATGTTAGTAAATTTCTTTCAAAACTGGCCAACTTCACAAATTTCACACCGACGAGCTTCGATCTGGGGGAGTTTCAGCGCCGTGCAGGACATAAAATTGAAGACATGCTAATAAAGTGCAAATGGAGAGACGAAAAGTGTTCAGCAAAAGACTTCACACCCATATTGACCGAGATGGGCCAGTGTTATCAGTTTAACAAAAACCCCATCACAAATGATAGTGGTATTCCAATTTACAGAAATGTTTCAAAGGGTGGTCAAGGCAATGGCCTTAAACTCCTTCTTGTCACGGAAGAAGAACAATACTTGCCTCCTAACATTGATAAATTCGATGTGAGTCTTGAATCAGGATTCAAAGCTTTGGTTCACGACAACGACATTCCTCCTTTAGTTCAGCAGTTAGGCATCGGTCTAGCTTCGGGTTTACAGACATTCGTTGTACTTCAGAAAGCAGAGGTTAAATACTTGGGCCAGCCATGGGGCGTCTGCTTGAAATCAGAAAATTCCCCTGACAAGATTTGGAGCCGCTACAGCGATTCATCTTGTAGAATAACATGCGAAACGCAACATATTGTAAAGAAATGCAAATGTAGATTTCCATTTATGCCAATCTTAAAAACTGATCCTAATTCAAATACCATCTGCACAACAAAGCAAATCAAGGACTGTGGCACAAGGGAACTCGAAAACATCGAGGACGGTTCGGCGTGCGGACCCGATTTCTGCATCAAACCTTGTGAAATTACTCGCTACAGAAGTTCTTTGTCATCGGTAAAATTTCCCAGCGCTCAAGGAGAAATCAAACTAAGCGACGCACTGAAGAAGCACACAGAGGGAAAAGGACGTAATTACACATCAGGTTCAGAAAAACTATCAGATCGCGCTCTTGTCGTTTCCATCTACTTCCAATCACTCAGTCTGGAGAAAATTGAACAAGTTGAAGCATATGACTTTGCTGCCATGCTTGGTGACATCGGAGGTCTGATGGGTCTTTTCATCGGAGCAAGCGCAATGACATTATTTGAGTTCTTCTTCTTCTTTTGGGACTTTATTCTTCTTAGAAAAAAACCTAGCAACCCAGAATCCAATAACCGCCAGCCTCAAATGGCCAACGACCGGGACTGCATGCCACCGGGCGCCGTGGATGCCTCCAAATTCGTTCTTCCATTGCATAATGAATACAACCCACAGTTAATCCATGTCTCCGCCCTTCAACATCAAAACTCTGTTCAGCTGCCAATTCCAAGAATAGAGCAATACAACAAATTTTCACCCCTCGTCAAACGCAGCGTCATGCCAGTCCATCCCTACTCAGTAAATTTCTTCGAAGAGAAGAGTTCGAAACTGCTTATATCCAACCCCTTTCAAGAGAAGTACTGCGGATCC

>CmacropygaASIC_Cmac.rna.tri.3223.1

GTCGACATGCATGGAGCAAGATATGTGGTGCTGCCAGGTGCAAGGATAACGATAAAGCGAGTGTTGTGGACACTGGTGTTTGCCCTCTTCTGGGTCTACTTCATCAGTGTGAGTGTGAAGAAAGTGCAGTTCTACTACTCCTACCAACACATCACACAGCTAGACGAGGAGTCACAGGCCTTAGACTTTCCTGCCATCACGATCTGTAACTTAAACCCAATTCGCCTGAGTGCATTCACTGCCTCCGACCTCTACAACGCTGGCAAAATGTACGACATTCTGGATGACGACTCCAAACTGAAAGAGGACCTTTTTTCAGAAGACAACCCAAAGACAAAACTCCTCATGAGGACTCTGGCTTCAATGACAGATGATGACAAGAAGAAAGACTTCAACCTGGAAGAGTTCCAAATGCGAGCAGGTCACAAGATGAAAGACATGCTGAAAGCGTGCAAGTGGAGAGACATCAAGTGCACTTCGGACGATTTCACCCCGGTTCTGACCGAGATGGGGCTGTGCTACCAGTACAACGTGAACAAAACATTCGACGATGAAACAGGTGACTACCAATTTAAGAAAGTGTACAAAGGAGGTGCTGGAAATGGTCTCAAAATGCTTCTCATTACCCAAGAAGACGAGTATTTGCCACCACAGATTGAGAGCTTCGAGACCTCCCTTGAGACTGGCTTCAAGATAGAGATCCATGATCCAGACGTGCCCCCTCTGGTCCACCAGCTGGGCATTGGCATTGCCCCTGGGCTTCAGACATTTGTGGTTCTGCAAAAGGAGCAGGTGACGTACCTGACTAAGCCGTGGGGTGAGTGTGTGCAAAAGTACGAGAAGACTATCAACTTCACAAAGTACTCGGATCCAGCATGTAGGATAGAGTGCGAGACCCAGCATATTGTGGACGAGTGCTCCTGCCGATTACCCTTCATGCCAATTCTGAGCAACAAGCCCAAAAGTAGCATTGTGTGTTCTCCTCAACTGTATAAGGAATGTGCGAGAGAGAAGTTGAACAAAATAGAAGACGGGGAGGCTTGTGGCCCAGAGACCTGCATCAAAGCCTGCGAGATTTCAAGGTACAAAAGCAGCATGTCATCAGTGAAGTTCCCCTCTTACCTGGGTGCAATCAAACTGAGCGAAGAATACCCAGGCGTGTTCACAAAGGACAGCACCAACTCTGGAGGTGGGGGTGACTCGGGTGGCGACGGAGGGGGCGATTCTCGAAGTGGTGGAGACGGAGGTTCGGATCGGTCATTTGACGGAGGGTCTGTTAAAGATGATGTGAAAGCTAAGGCTACTCAGTCTTATTGGAAAGAGGTTGAAAACAAAATATCAAAGCAAGCCTTGGTCGTGTCTATCTACTTCCAGTCTCTGAGTTTGCAGACTATTGTCCAGGTCGGAGCATATGACTTTGCAGCCATGCTTGGTGACATTGGTGGTCTGATGGGTCTATTCATCGGAGCTTCTGCCATGACACTTTTCGAGTTCTTCTTCTTCTTCTGCGACTCCCTCATCCTGCGTAGGAAGGGTGCCGGCAACAGGAACAGCAATGCAGGAGGGGGATCCGATGATGAGAAATCAGAAATGATAGGGGACAATTTGTTGCGAGGCCACGATGGTCAACCCCTTAAGAGTGTGCTGTTGGCTGCCCCTGGGGCCGTGGATGGGTCCAAGTTCGTGTTCAATGTCAACCACAACAACGACCACAACGCCGCCGCTGCCGCCGACTACAATGCTGCCATGTCCAGATTACTTCCACAGTTTAGCGCCCATCCTTTGCACAACTCATTTCAACAGCAACAACAGCAACAGCAACTGTTAAACCAACAGCGAGGCTACTCACCCCTGATGAAAAGGGCTAACTACCCCTCGTCAGGTGCATTCGGCCAACCGCCAAACCAGAACCAACCCAACTCAAATGATGTCGGTAGTCTCTTGTCCCCCCACGATCCCTCCAGCTACCCTGCGTCTCCAGTACCCCCCGCGAATGGTGGAATAGGGCTTCCGGTGACCTCATTTCCCGGAGCTCAGCAACAACAGAATATTCCACAGAACATGCTCGTCACTGCACCCTATCAACAGGGGGGTTTGCCCGGAGCGGGGGGCAAAATATTCCTGGCCAACAGTCTTCCTCAAGAGCGATATTGCTCTAGAGGATCC

>TtransversaASIC_AB1973

GTCGACATGTCTTCTACAACACACGAACCAACAGACTCGGAGATGAGGCACAGGACTCCTGCAAATAATACACCAGGAGTTCTATCACATAATCAGCTTGAACTTAAAACAAAGGAAAGAATAGAAACATTCGGTGAAGATTGTGATTTCCATGGCATCAAAAGAATATTACGACATGAATATCACTTATGGAGAAGGATTACATGGCTCATGTTTTTTGTCTTTGGTTTAACATTTTTGACATATCAAATCACAGAGTCAGTCTTGACTTACCTGAAGTATGAACATGTTACTAAAGTGGATATGATGTACTCCAATACAATGGAATTTCCTGCCGTTACCATATGCAATCTTAACCAATTTAAAGTCTCTGCATTAACAGCTCTGGATTTGCATCACTTCGGACAAAAGCTAGGGATTTTCCTTAATGGCACATACACCCTTACACATCCCGACCAGTACAACAGTACATGGGTACAGTGGGTGAATGGAATCAACTGGACTGAAATAGCCAGTGGTCCTGATGACTTTGATGTGGAAGAGTTCTTTAACAGAACAGGTCATCAGAAGGAACAAATGATTTTGTTATGTCTTTGGAGAGGGGCAGCCTACAACCACTCTGATTTCCAAATGACACACACCCATCTAGGAAATTGTTTTACATTTAACCACGGAAAAGATGATGTTAACTTTCACACAAGAAATGCTGGAACTGAAGCAGGTCTAAAGTTGTACTTAAATGTTGAACAAGATGAATATCTAGAAGGTGAAGACTCTGCTGATGCTGGATTCAAACTGTTAGTTCACGATCAAAAAGATCCCCCATTTGTGAAAGAGTTGGGCTTTGGGGTTGCTCCTGGATATCATTATTTTGTGTCTTTACAGAAACAAAAGATTAAAAATCTGTCTAAGCCATGGGGTAATTGCCAGTCAGGACAACTGGAATACTATGACCACTACACAATTCCAGGCTGTCGGATAGAATGTGAACTGAAAACAGTCAAAGAAACATGTGGATGTCGTCTTATCGAGATGCCAGGAAATGATACAGTGTGTCACGGCAATGCATACATGGGATGTGCATATCCTGCATTAGAGAGAGTGGAACACTCGGACAATTGTGTTTGTCAAAATCCATGTGAATTTACACACTATAAACATGCCACAACCTTTGTGGAGCTAAGAGAAAATACAGTGGACAGGATTCAAAAGAAAAATATTCAGTATACAAAAGAAAAATTGAAGTCCGACCTGGTAATACTGAGTGTATACTTTGAGAGACTCAATTATGAAGAATATGAACAACTGCCAGCCTACACTCTGGTTGATCTATTCAGTGCCATTGGTGGTAATATGGGTCTCTTTATTGGAGCAAGTGCCTTGACAGTTCTCCATTTGATGGAGTTCATTGGGTCAGAGTTAACTCTTTGGATGTGGAACAGAAAAAACAAGAAACAAAACCAAGTAATGACAACAGTCACTCCCATGAAACTACCAATACAGGATGGATCC

>Lanatina_ASIC_g20471.1

AAGCTTCCTTGGCAAGCATGCAGAAAAAGACCTCGTGGATAGAGACTGCCAATCGGTTAGTCGGCAACACCAAAAGACAGAAGCAGTCAGAATCCACAGAATCAAAAGCTAGCAAAGAAAGCGGCTCCGTCAGCGAAGGCCTGGAACAATTTGGAGAAGACACCGACTTCCACGGACTGAAGCGTGTCTTCAGGAGAGACTATACACTGTTACGCAGAACCTTTTGGTTGTTTTTCTTTCTGGCGGGTCTTACGGGTTTTATCGCCAACACAGTTGACAGGCTGAGCTATTACTTGCAGTATCCACACTCGTCTGAGCTGGACGTGATGTACGATATGGAGTTAGAGTTTCCAGCCGTAACCATATGCAATATGAACTCCTATAGGCTATCCGCTTTGACAGACGTTGATATATTACATTTTGGTGAAAAACTACACATTTTAGACGAAAACCGCCGTCTTATACACCCGGAATATTATAACCAAAGCTGGGTGGATTGGGTGCACGGTATTAATTGGACAGAGTTAGCTGCCAACGATGACGAAGAAAATTTTGACGTGCTTGAATTTATACGTCGAACCGGCCACCAGCTGGAAGACATGATACTTCTGTGTTCGTGGAAAGGGCAACACTGTGGCCCGGAAAACTTTACCTCGGTTTTCACTCATTTTGGCATCTGTTATACTTTCAATGCTGACCACTCTTATAAATACGTATCACGAAAGGCGGGGGCGGGGAACGGGTTGAAGTTATATATCAACATCGAAGAAGAGGAGTATTTGACTTCGGATGTCCTAGCAGGGCAAGATGCTGGACTGAAGATGGTTATTCACGCTCAGGAAGAGCCGCCGTTTGTAAAGGAGTTGGGATTTGGAGTGCTACCGGGGGACCACCACTTTATAGCCATACAGAAGAAATACGTTCACAACTTGGTTCCTCCATGGGGAAATTGCTACGACGGGAAACTCAAGTATTACTCCCATTATAGCGTCCCTGCCTGTAGGATAGAGTGTGAGACAGATACCATCGTAAAAGAGTGTGGCTGCAAGCTGGCTGAAATGCCAGGCAACACATCCGTATGTCTAGGACATATGTACATGGGATGTGCCTATCCAGCCTTAGAGGAAGTGGAGCACTCAGACCTGTGCTCCTGTCAGAATCCATGTGATATGACCACCTATAGGCGAGACATCTCTTCCGTAAGGCTTCGAGATACTACCTTAGATATCATAGCCGAAAATAACCCGCAAGTTAAACGGGAAACACTAAGAGATAACCTACTGGTGTTGAACATCTATTACGAAGAGCTGTGCTATGAGACCATCAGACAAATCAAAGCATATTCCATACCAGCATTACTTAGCGACATAGGAGGCCAGATGGGTTTGTTTATAGGAGCTAGTGTTCTGACTCTGCTGCATGTGATCGAGGCCGTTGGGGCTGTTGTTGGTGGAAAGTTCCTCAAAACAACGAAGCAAGGGCGTGTCAGTACAACTCGGGTCCAAAGTTTGAAGGGATCCGAGCAGAAGCTCATCAGTGAGGAAGATCTCTAA

>PharmeriASIC_comp166140

GTCGACATGGAAGAGCCGGAGACAGAACCTCTCACACAGAACGGACTGAACCAACGTTTGGGAAAGAAAGCGGTCCAGGACTGTATTATAATTGACTTGGTCAACAATGAAATACCTGAGCACGAGCGAAAGGAAACGTTGAAGACTCGTTTTGAAAACTTTGGAGAAGACACCGACGTCCACGGATTAAAATACATACTACGCGAGGACAGTCCAACGTATAAAAAAGTTATATGGATGATGTTGCTTGTAATTAGCCTTGGCTACATGACATTTGAAATTTATGGAAGGTTTTCGTGGTATTTTACTTACCCACATGTCACCAAGGTCGATGTTGTGTTTCGCAATAACATGGAATTTCCTGCATTCACCATTTGTAATATGAACAAATTCCGCGCGTCGGCCATGACAGATGTGGATATATTAAACATGGGAAAAATACTAGGAATTGTGAATGACAATATGAAACTGCACCACGGTGATCATTATAACGAAACATTCGTGAACTGGGTAAACAGCATGAACTGGACACGCGTCCGAGAGAAAAACAAAACATTTCACCTGGAAGAATTTTCTAGACGAGTTGGGCATCAGGCCAAAGACATGATTGTCTACTGCCGATGGAAGGATGAGTTATGCGGACCTGACAATTTTACTCACAGCTTCACGCATCTTGGAAACTGCTATACGTTTAACGACAATCAAAAGTTTGCGGCCCGAAAGGCGGGTGCCGGCAATGGACTAAAGTTATATCTTGACGTTGAAGAATTTGACTATCTTGAAACTGCAGATGCCGCAGACGATGGCCTGAAGGTCATAGTTCACAGTCAAAAAGAACCGCCTTTTATAAGAGAACTTGGATTTGGACTTATGCCGGCACAACACCATTACATTGGTATAAGGAAAGCCGAGATAATCAATTTACCAAAACCATATGGGACCTGTGCAGAAGATTTTCCAATGCGAATGTTTGAGCATTATACAATACCTGGATGTAGAATCCAGTGTGAAACGGAACACGTTGTACAGGCTTGTGGATGTCGTCTGCCAGAAATGCCGGTGTCATCTGACGCACCAATATGTTCGCCTCTTCAGTACGACGACTGCGCACTGGCAGAATTAATACGTGTCAGCGAGTCAGACGACTGTGTATGCCAAAGCCCGTGCCACCTCGATGACTTCCGGCTAACACATTCCAGTGTTAAACTACGCCCAGAAACAATAGAAAAGCTTCAGACCTTGCACGCACACGTTCCGAAACATCTCAAAGCGCATAATATTGTTGTGATGGACGTCTTCTTCGAGGCGTTGAGTCTAGAACTTATAGAGCAAAAAGTGGCGTACCCGTGGCCAAGCTTACTTGGCGACATTGGCGGGCAGATGGGCTTATTCATTGGCGCCAGCGTCCTGACAATCCTGCATGCTGTCGAGTTTTTCACCGATGAAATCGCTAAGAGTTGTAAAAAGAAAGCAGACAAGACGGACCAAGTGAACCCAGATGAAACTAGGGCGTCCATGGGTCCCAACGAATCTGCCGTGGTCGTCAAGGAAGCTGTGATAGGATCC

>OfusiformisASIC_48178.5.p1

GTCGACATGACTGAAACAAAGGAAAAACAAACTGATCCTGTCATTGAGCATTCATTTTCAACAAGACTTAAGGAATTTGGTGAAGATGTTGAATTTCATGGAGTGAGACATCTGTTTAGAGATAATTCAATTGTGACAAAAGTGACATGGATTTTACTGTTTCTGTGTGGATGCAGTTGGCTGACCTACCAGATACATGATCGAATTATATATTATTATAAATATCCGCATATAACCAAAATTGATAAACTTTATGTTCCTTCATTAGATTTCCCAACAATCACAATTTGCAACATCAATACATTCAGAAGGCACAAATTAAATGAGTATGACCTCCTTCACTATGGAACAAGCCTGAATATTCTTGATGAGAACCGGACCCTTCTTCACCCAGAGCACTATGATAAAGCCTTTGATGACTGGGTTTACAGTATCAATTGGACAGATGTGGAGATCCATGACCGTTCCATCAATCACAGCATGGAGGAGATGTATGAAAGAGCAGGACACCAGATAGAGGATATGCTCATCTACTGCAAATGGAAACAACAGGAGTGTTCTGTTGCTAACTTTACCTTGATTAACACTCACTATGGGCGATGCTATCAATTCAATTCTGGCAAGGATGGTGTCAAACATCAGTCATTTAAAGGAGGTAAAGCAAATGGTTTGAAACTTTATCTAAATGTAGAGGAACTGGAGTACCTAAACTACATGGAAGCTTCAGACCTTGGCTTTAAAATTCTGGCACATGACCAGGATGAACCACCACTTATACAGGAATTGGGATTTGGAGTGACTACTGGCAACCACTACTTTATTGCATTGGAAACAGAAAGGGTGACAAGCTTACCAGACCCTTATGGCAACTGCGAAGAGGACCACAAACTTGACCATTATGATCATTACAGCATTCCTGCGTGCAGAATTGAATGCGAGACATTAATAGTTGAGGAGAAATGTTCATGTAGGCTTGTTGAGATGCATGGTGATCATGGTATCCGTGTTTGTACTGCTGAGGAATATCATGACTGTGCTCTGCCAACATTAGAGTCGATTACTGAGAGTGACACATGTGTATGTCAGAACCCATGTGAGCTGACCCAGTTTCAACACTCTATATCCTCTGTAAAGCTTCGTGAGAGTGCAGTTGAGATGATACATGGCCACACTTCAAGCAACATAAATCTGACTGAGTTTAAGTCAGTGGAGTTCATTGAGAAAAACCTTCTTGTTGTGAACCTCTTCTTTGATTCATTGAATTACCAGTACATTGAACAGACGGTTGCATATCCTGGTGTGTCACTATTAAGTGATGTTGGAGGTCAGATGGGATTGTGCATTGGGGCCAGCATTCTGACAGTTTTACATTTGGTTCAGTTTGCAATTGGGGAAATCATTAAGAAGTTTCAGAAGAGAGAGAATAAAGAAGTCAACACCAATGTTATTCATGTGGCATCTGCAAATCCTGTAGATGATAAACTTGGATCC

>Scalifornicum_ASIC_c469942.i1

GTCGACATGGAGCTACGGGACAACGGTTACGGGATCGGTCGGTCCCGTAAAATTAACAACAATGCTTACTCCGAATCTCTATCGTTTAACATCGCTAGTCGCTCCTTGTCAACGGACGGTTCGAACTTAGAAAGCAATTCCCGCTCGGCGTCTTTACGGTGGGTGCATTGGGCATCGCAGGTGTCTGATATACACGGAGTTAAACACATTGTCAGCGAATCTTCACGTTTTCGTAAGCTGCTTTGGACGCTCTTTGTGTTAACTTCATTAGGCGTGCTTCTTTTCCAGTTTTGTGAGTCAGCATGGAACTATGCTCAATTCTACCACATCACTAAACTTGACGTTGAATATCTACCTCATATGCCATTTCCCGCCGTTACTGTTTGCAATTTCAACAAATACAGAAGATCGGCGATCACACCTACAGATATGGTCCACATTGGTCAACCATTAGGTCTGGTGGACACGGAGAGAAATTTAAACAATCCAGAGTTATTCAGTGATGAGTTTATGGACAAATGGAACAATACTGATTGGGAAGTAGAAATGAAAAAGCCGTTCGACTTCACGGAGTTCACACGCAGAGCAGGACATCATTTTGACGAGACCATCCTCGAGGCTCTTTGGAATGGTCATTCATGTACCCCAGAAGACTTCAGAGACTTTCTAACTCACTATGGCAACTGCTTCATCTTCAATCAATACGAAGAAGATGAAGACCAATACCACTCAATGAGAGCTGGAACAGGTAATGGTTTGAGAATTGTGCTTGATGTCCACTCCCATGAGCATATACCGACGACAGACCTGGAGGATTCATTTATTAATGTTGGGTTTAAATTGATGATTCATTCCCCACACGAACCACCGTATCTTAAACAACTAGGATTTGCTGTTGGACCGGGAAATCATTATTTTCTAGCACTTAAAAGACAAGAGATAATTCGATTATCAACTCCATACACTAGCAAAGTATGTGAAAAGTCTTCAGAAGGAACCAAACACTTCCATGAATACTCAATGTCAGCATGTCGAATCGAATGTGAGACTTCATTGCTCGTTCAAGAGTGTGGATGCAAACTTGTTGAGCAGCCTGGCAACGCGTCAGTGTGTTCACCCCAGCAGGTCGCACTGTGTGCTCACGAGACATTAGATGAATACATTGAAGGACACATAGAATTTGACTGTCCCTGTGATATACCTTGCAAAAGTGAGTTGTACCCAGTTGATGTCTCTAACAGTGGTTTGAAGAGGGATATCACCGGTAAATCAGTTGGATTGTCCAACTACTCAATAGAATACATCAAAAATAATATTGTCATGCTGACGATATTTTATGAAGAACTAAACTTTGAGACGATCGAACAGCTTCCAGAAATGTCTATCGTAGACTTGCTTGGTCAGCTTGGTGGCAACATGGGGCTGTTTCTTGGTGCTAGTATACTTACCATTTTCCAAATTTTCGAATATATCTTTGATGAGTTCAAATTTTGTATATCGCTTGGGGCTGATCGCAACCAACAGAAGAGAAAGAACAAGAAGAAAATTTATGAATGTGATGACAAAAGCCTTCAAGCACCATTGTCCATACCATCACAGCAAGCAGGCATAATACGTGGATTTAGAAATACGACTGTGGGATCC
